# Cytotoxic Natural Products from the Jurassic Relict *Osmunda regalis* L.

**DOI:** 10.3390/molecules29174247

**Published:** 2024-09-07

**Authors:** Andrea Estefania Carpinteyro Diaz, Lars Herfindal, Bjarte Holmelid, Cato Brede, Heidi Lie Andersen, Anni Vedeler, Torgils Fossen

**Affiliations:** 1 Department of Chemistry and Centre for Pharmacy, University of Bergen, N-5007 Bergen, Norway; andrea.diaz@uib.no (A.E.C.D.); bjarte.holmelid@uib.no (B.H.); 2 Department of Clinical Science and Centre for Pharmacy, University of Bergen, N-5009 Bergen, Norway; lars.herfindal@uib.no; 3 Department of Medical Biochemistry, Stavanger University Hospital, N-4011 Stavanger, Norway; cato.brede@uis.no; 4 University Gardens, University of Bergen, Allégt. 41, N-5007 Bergen, Norway; heidi.andersen@uib.no; 5 Department of Biomedicine, University of Bergen, N-5009 Bergen, Norway; anni.vedeler@uib.no

**Keywords:** *Osmunda regalis* L., Osmundaceae, fern leaf, cytotoxicity, lactones, aromatic compounds, 2D NMR

## Abstract

The Jurassic relict Royal fern, *Osmunda regalis* L., is widely distributed across temperate zones in the Northern and Southern hemispheres. Even though this species has been utilised for centuries as a medicinal plant, its phytochemical composition mainly remains unknown. As part of our ongoing research to identify potential lead compounds for future anticancer drugs, 17 natural products were characterised from the aerial parts of *Osmunda regalis* L. Fifteen of these compounds were identified in this species for the first time, including the six previously undescribed compounds kaempferol 3-*O*-(2’’-*O*-(2’’’-α-rhamnopyranosyl)-*β*-glucopyranosyl)-*β*-glucopyranoside, quercetin 3-*O*-(2’’-*O*-(2’’’-*α*-rhamnopyranosyl)-*β*-glucopyranosyl)-*β*-glucopyranoside, kaempferol 3-*O*-(2’’-*O*-(2’’’-α-rhamnopyranosyl-6’’’-*O*-(*E*)-caffeoyl-)-*β*-glucopyranosyl)-*β*-glucopyranoside, 3-methoxy-5-hydroxy-4-olide, 4-hydroxy-3-(3’-hydroxy-4’-(hydroxyethyl)-oxotetrafuranone-5-methyl tetrahydropyranone, and 4-*O*-(5-hydroxy-4-oxohexanoyl) osmundalactone. The molecular structures were determined by combining several 1D and 2D NMR experiments, circular dichroism spectroscopy, and HRMS. Determination of cytotoxicity against AML MOLM-13, H9c2, and NRK cell lines showed that two isolated lactones exhibited significant cytotoxic activity.

## 1. Introduction

**Figure 1:**
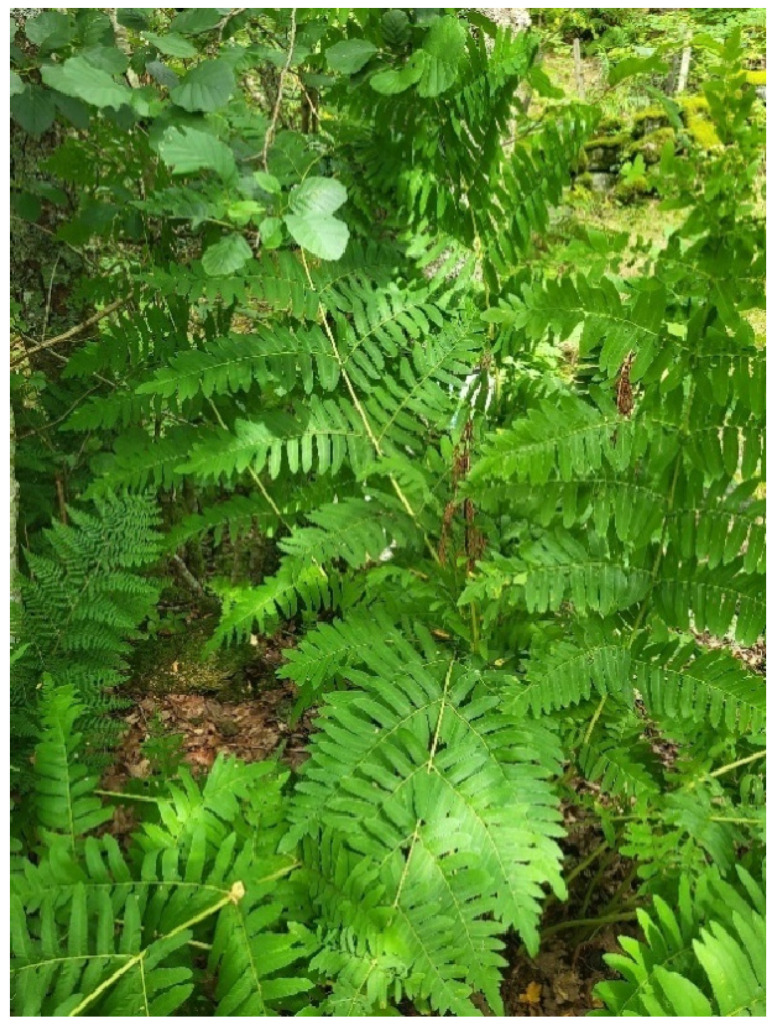
Fern leaves of *Osmunda regalis* L. Photo: Heidi Lie Andersen.

The Osmundaceae family comprises several living genera. This family is classified as a basal group of leptosporangiate ferns, which have undergone little morphological and anatomical change since the Mesozoic time, and, therefore, some authors consider them as a “primitive” species [1,2,3]. *Osmunda regalis* L. (Figure 1), also known as royal fern, is a species with widespread occurrence in the temperate zones of the Northern and Southern hemispheres, widely distributed throughout Europe, Southern Africa, America, and New Zealand. It is deciduous or evergreen in warmer regions, a terrestrial fern, typically growing to a height of 1 to 2 m. The frond is bi-pinnate, meaning that each leaf is twice divided into pinnae and smaller pinnules. The fern is often found in moist woods, swamps, lakes, or stream banks [4]. In some countries such as Spain, Italy, and Poland, *O. regalis* is a threatened plant species with an ecological impact in grazing for wild animals [5,6].

The royal fern has been widely used for its medicinal properties in traditional medicine for several centuries. Hieronymus Brunschwig, a German surgeon, alchemist, and botanist, wrote about the plant’s potential use in treating cancer, fistulas, and fractures in his book *Kleines Destillierbuch* [7,8]. Moreover, in some regions of Northern Spain, such as Galicia, Asturias, and Cantabria, the plant has been traditionally used to treat bone fractures and alleviate muscular pain [9]. Even though *O. regalis* has been utilised as a medicinal plant for more than five centuries, only scarce information about its chemical constituents is available in the current literature. Only a few compounds have been identified, including sitosterol, which has been found in different parts of the plant [10,11], as well as the steroid Ecdysterone (20-hydroxyecdysone) [12]. Several relatively nonpolar compounds such as saturated fatty acids [13,14], alkanediols, ketoaldehydes, methyl palmitate, and other fatty acid esters [15,16] have been reported from this species. Anthocyanins based on the aglycones pelargonidin and cyanidin have also been detected, where the cyanidin derivative is indicated to be an acylated anthocyanin [17]. Further phenolic compounds, such as tannins and gallotannins [18], as well as a multitude of common phenolic acids, including caffeic, *p*-coumaric, *p*-hydroxybenzoic, cinnamic, and vanillic acids, have previously been identified from this species [19,20]. Only a few specific compounds of this species, including osmundalactone [21] and osmundalin, where the latter compound is the glucoside of osmundalactone [15], have hitherto been reported in the current literature.

This paper describes the characterisation of 17 natural products from the aerial parts of *Osmunda regalis*. Fifteen of these compounds, including six undescribed compounds, are identified in this species for the first time. As part of our ongoing research to identify new lead compounds for future anticancer drugs, the cytotoxicity of the isolated compounds towards acute monocyte leukaemia (MOLM13), rat cardiomyoblasts (H9c2), and kidney epithelial cells (NRK) were examined. The theoretical framework for this study is a fundament of the work presented in the doctoral thesis of Carpinteyro Diaz [22]

## 2. Results and Discussion 

A methanolic extract of 2.1 kg of leaves from *Osmunda regalis* was concentrated under reduced pressure and subjected to a liquid–liquid extraction (LLE) with petroleum ether followed by ethyl acetate. The components of the aqueous and ethyl acetate extracts were further separated by gradient XAD-7 adsorption chromatography, Sephadex LH-20 gel filtration chromatography, and preparative HPLC.

**Figure 2:**
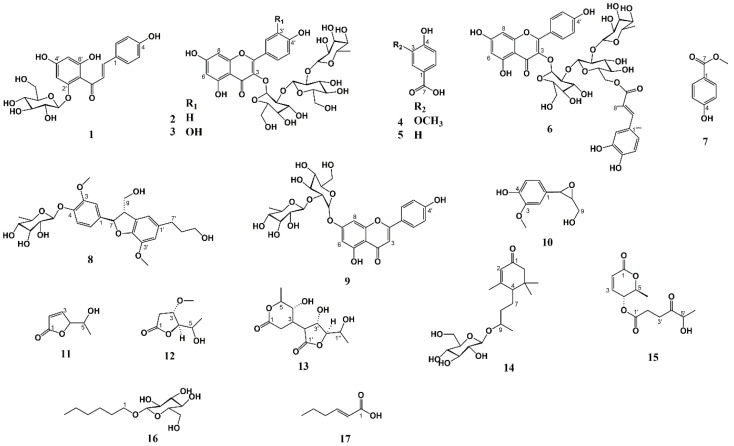
Molecular structures of compounds 1–17 characterised from *O. regalis* (L).

The eleven known compounds chalconaringenin 2’-*O*-*β*-glucopyranoside (**1**), vanillic acid (**4**), *p*-hydroxy-benzoic acid (**5**), *p*-hydroxy-benzoic acid methyl ester (**7**), dihydrodehydrodiconiferyl alcohol 4-*O*-α-rhamnopyranoside (**8**), apigenin 7-(2’’-*O*-α-rhamopyranosyl-*β*-glucopyranoside) (**9**), epoxyconiferyl alcohol (**10**), 5-hydroxy-2-hexen-4-olide (**11**), blumenol C 9-*O*-*β*-glucopyranoside (**14**), n-hexyl-*O*-*β*-glucopyranoside (**16**), and 2-hexenoic acid (**17**) (Figure 2 and Tables S1–S9) were identified from the fern leaves of *Osmunda regalis* L. The identifications were based on a combination of several 1D and 2D NMR spectroscopic techniques. These compounds were detected in this plant species for the first time, except for vanillic (**4**) and *p*-hydroxy-benzoic acids (**5**). 

The UV spectrum of compound **2** recorded online during HPLC analysis exhibited λ_max_ values at 346 and 266 nm (Figure S63), which matches a flavonol derivative [23]. The aromatic region of the 1D ^1^H NMR spectrum of **2** (Figure S1) showed a 4H AA’XX’ system at δ 8.05 (‘d’ 8.9 Hz, H-2’,6’) and δ 6.93 (‘d’ 8.9 Hz, H-3’,5’), which is consistent with a *p*-substituted B-ring, and a 2H AX system at δ 6.42 (d 2.0 Hz, H-8) and δ 6.18 (d 2.0 Hz, H-6), which is consistent with a kaempferol derivative. The sugar regions of the 1D ^1^H and 1D ^13^C CAPT spectra of **2** showed the presence of three sugar units. All ^1^H and ^13^C resonances of these glycosyl substituents were wholly assigned by the 1D ^1^H selective TOCSY spectra of each glycosyl substituent, in addition to the 2D ^1^H-^1^H COSY, the 2D ^1^H-^13^C HSQC, the 2D ^1^H-^13^C HSQC-TOCSY, and the 2D ^1^H-^13^C H2BC spectra of **2**. The sugar units were identified to be two *β*-glucopyranose units and an α-rhamnopyranose unit, respectively, by the observed ^1^H coupling constants in the 1D ^1^H and the 1D ^1^H selective TOCSY spectra, in addition to the 17 ^13^C chemical shift values belonging to these sugar units observed in the 1D ^13^C CAPT spectrum of **2**. The anomeric coupling constants revealed the *β*-configurations of the anomeric carbons of the glucosyl substituents and the α-configuration of the anomeric carbon of the rhamnosyl substituent (Table 1 and Table 2).

Assignments of the ^13^C resonances belonging to the aglycone and the inter-residual connections were determined by the 2D ^1^H-^13^C HMBC experiment. The cross-peak at δ 5.71/132.9 (H-1’’/C-3) confirmed the linkage between the glucopyranosyl unit and the aglycone at the 3-hydroxyl. The downfield chemical shift of C-2’’ (δ 77.8) of this glucosyl unit indicated the presence of a sugar substituent at this position. The cross-peaks at δ 4.97/77.8 (H-1’’’/C-2’’) and δ 3.70/100.2 (H-2’’/C-1’’’) confirmed the linkage between the inner glucosyl substituent and the terminal glucosyl substituent to be at the 2’’-position. Cross-peaks at δ 5.05/77.0 (H-1’’’’/C-2’’’) and δ 3.23/100.3 (H-2’’’/C-1’’’’) proved the linkage between the terminal glucosyl unit and the rhamnosyl unit to be at the 2’’’-position. Thus, compound **2** was identified to be the previously undescribed compound kaempferol 3-*O*-(2’’-*O*-(2’’’-α-rhamnopyranosyl)-*β*-glucopyranosyl)-*β*-glucopyranoside. A sodium-added molecular ion [M+Na]^+^ at *m*/*z* 779.1999 corresponding to C_33_H_40_O_20_Na^+^ (calculated: *m*/*z* 779.2005; Δ = −0.73 ppm) observed in the HRMS of compound **2** confirmed this identification (Figure 2 and Figure S53).

Compound **3** exhibited a UV λ_max_ absorption at 352 and 256 nm (Figure S64), which resembles a flavonol derivative [23]. The aromatic region of the 1D ^1^H NMR spectrum of **3** (Figure S13) showed a 3H ABX system at δ 7.49 (d 2.3 Hz, H-2’,6’) and δ 6.89 (d 8.5 Hz, H-3’,5’), which is consistent with an *m*- and *p*-substituted B-ring, and a 2H AX system at δ 6.38 (d 2.1 Hz, H-8) and δ 6.17 (d 2.1 Hz, H-6), which accords with a quercetin aglycone. The 1D ^1^H and 1D ^13^C CAPT spectrum of **3** indicated the existence of three sugar units in the sugar regions. The ^1^H and ^13^C resonances of these glycosyl substituents were entirely determined by the 1D ^1^H selective TOCSY spectra of each glycosyl substituent, in addition to the 2D ^1^H-^1^H COSY, the 2D ^1^H-^13^C HSQC, the 2D ^1^H-^13^C HSQC-TOCSY, and the 2D ^1^H-^13^C H2BC spectra of **3**. The sugar units were identified to be two *β*-glucopyranose units and an α-rhamnopyranose unit, respectively, by the observed ^1^H coupling constants in the 1D ^1^H and the 1D ^1^H selective TOCSY spectra, in addition to the 17 ^13^C chemical shift values belonging to these sugar units observed in the 1D ^13^C CAPT spectrum of **3**. The anomeric coupling constants revealed the *β*-configurations of the anomeric carbons of the glucosyl substituents and the α-configuration of the anomeric carbon of the rhamnosyl substituent (Table 1 and Table 2).

Assignments of the ^13^C resonances belonging to the aglycone and the inter-residual connections were determined by the 2D ^1^H-^13^C HMBC experiment. The cross-peak at δ 5.70/133.2 (H-1’’/C-3) confirmed the linkage between the glucopyranosyl unit and the aglycone at the 3-hydroxyl. The downfield chemical shift of C-2’’ (δ 77.20) of this glucosyl unit indicated the presence of a sugar substituent at this position. Cross-peaks at δ 4.94/77.20 (H-1’’’/C-2’’) and δ 3.73/100.4 (H-2’’/C-1’’’) confirmed the linkage between the inner glucosyl substituent and the terminal glucosyl substituent to be at the 2’’-position. The cross-peaks at δ 5.06/76.9 (H-1’’’’/C-2’’’) and δ 3.23/100.3 (H-2’’’/C-1’’’’) ratified the linkage between the terminal glucosyl unit and the rhamnosyl unit to be at the 2’’’-position. Thus, compound **3** was identified to be the previously undescribed compound quercetin 3-*O*-(2’’-*O*-(2’’’-α-rhamnopyranosyl)-*β*-glucopyranosyl)-*β*-glucopyranoside. A sodium-added molecular ion [M+Na]^+^ at *m*/*z* 795.1951 corresponding to C_33_H_40_O_21_Na^+^ (calculated: *m*/*z* 795.1954; Δ = −0.37 ppm) observed in the HRMS of compound **3** confirmed this identification (Figure 2 and Figure S54).

The UV spectrum of compound **6** recorded online during HPLC analysis exhibited UV-absorption maxima at 330 and 268 nm, with notably strong absorption at 330 nm, which matches a flavonol derivative acylated with a cinnamic acid (Figure S65) [23]. The aromatic region of the 1D ^1^H NMR spectrum of **6** (Figure S23) showed a 4H AA’XX’ system at δ 7.98 (‘d’ 8.8 Hz, H-2’,6’) and δ 6.83 (‘d’ 8.9 Hz, H-3’,5’) in accord with a *p*-substituted B-ring and a 2H AX system at δ 6.37 (d 2.1 Hz, H-8) and δ 6.16 (d 2.1 Hz, H-6), which accords with a kaempferol derivative. The sugar regions of the 1D and 2D NMR spectra of **6** showed the presence of three sugar units (Figures S23–S28). All ^1^H and ^13^C resonances of these glycosyl substituents were assigned by the 1D ^1^H spectrum of **6**, in addition to the combined information from the 2D ^1^H-^1^H COSY, the 2D ^1^H-^13^C HMBC, the 2D ^1^H-^13^C HSQC, the 2D ^1^H-^13^C HSQC-TOCSY, and the 2D ^1^H-^13^C H2BC spectra of **6**. The sugar units were identified to be two *β*-glucopyranose units and an α-rhamnopyranose unit, respectively, by the observed ^1^H coupling constants in the 1D ^1^H NMR spectrum, in addition to the 17 ^13^C chemical shift values belonging to these sugar units observed in the 2D ^1^H-^13^C HSQC and H2BC spectra of **6**. The anomeric coupling constants revealed *β*-configurations of the anomeric carbons of the glucosyl substituents and α-configuration of the anomeric carbon of the rhamnosyl substituent (Table 1 and Table 2).

Assignments of the ^13^C resonances belonging to the aglycone and the inter-residual connections were determined by the 2D ^1^H-^13^C HMBC experiment. At δ 5.71/133.2 (H-1’’/C-3), this cross-peak confirmed the linkage between the glucopyranosyl unit and the aglycone at the 3-hydroxyl. The downfield chemical shift of C-2’’ (δ 78.2) of this glucosyl unit indicated the presence of a sugar substituent at this position. Cross-peaks at δ 5.02/78.2 (H-1’’’/C-2’’) and δ 3.66/100.4 (H-2’’/C-1’’’) confirmed the linkage between the inner glucosyl substituent and the terminal glucosyl substituent to be at the 2’’-position. The cross-peaks at δ 5.05/77.1 (H-1’’’’/C-2’’’) and δ 3.69/100.4 (H-2’’’/C-1’’’’) established the linkage between the terminal glucosyl unit and the rhamnosyl unit to be at the 2’’’-position. The acyl moiety was identified as *E*-caffeic acid by the 3H AMX system cross-peaks at δ 6.95 d 2.2 Hz (H-2’’’’’), δ 6.84 dd 8.2; 2.2 Hz (H-6’’’’’), and δ 6.66 d 8.2 Hz (H-5’’’’’) and an 2H AX system at δ 7.36 (H-7’’’’’) and δ 6.16 (H-8’’’’’). A coupling constant of 15.8 Hz between H-7’’’’’ and H-8’’’’’ showed the *E*-configuration (Table 1 and Table 2). The downfield chemical shift of C-6’’’ (δ 63.4) of the glucose unit indicates the linkage between this sugar unit and the caffeoyl group. The cross-peaks at δ 4.30/166.5 (H-6A’’’/C-9’’’’’) and δ 4.19/166.5 (H-6B’’’/C-9’’’’’) confirmed the linkage between the terminal glucosyl substituent and the caffeoyl unit to be at the 6’’’-position. Thus, compound **6** was identified to be the previously undescribed compound kaempferol 3-*O*-(2’’-*O*-(2’’’-*O*-α-rhamnopyranosyl-6’’’-*O*-(*E*)-caffeoyl-)-*β*-glucopyranosyl)-*β*-glucopyranoside (Figure 2). A sodium-added molecular ion [M+Na]^+^ at *m*/*z* 941.2318 corresponding to C_42_H_46_O_23_Na^+^ (calculated: *m*/*z* 941.2322; Δ = −0.46 ppm) observed in the HRMS of compound **6** (Figure S55) confirmed this identification. 

The aromatic region of the 1D ^1^H NMR spectrum of **8** showed the presence of a 3H ABX system at δ 6.99 d 2.1 Hz (H-2), 7.04 d 8.3 Hz (H-5), and 6.85 dd 8.3; 2.1 Hz (H-6) and a 2H AB system at δ 6.68 d 1.8 Hz (H-2’) and δ 6.67 dd 1.8, 0.8 Hz (H-6’), which is consistent with dihydrodehydrodiconiferyl alcohol, where the aromatic rings are connected through a C3 unit showing a 4H spin system at δ 5.46 d 6.5 Hz (H-7), δ 3.42 dddd 7.4, 6.5, 5.3, and 0.8 Hz (H-8), δ 3.71 dd 10.8, 5.3 Hz (H-9A), and δ 3.60 dd 10.8, 7.4 Hz (H-9B). A further 4H spin system of a C3 substituent belonging to dihydrodehydrodiconiferyl was observed at δ 2.52 (H-9’), δ 1.68 (H-8’), and δ 3.40 d 6.5 Hz (H-7’). Two 3H signals belonging to methoxy groups were observed at δ 3.73 (3-OCH_3_) and 3.76 (3’-OCH_3_). The 20 ^13^C resonances belonging to dihydrodehydrodiconiferyl alcohol aglycone observed in the 1D ^13^C CAPT spectrum of **8** were assigned by the 2D ^1^H-^13^C HMBC spectrum and the 2D ^1^H-^13^C HSQC spectrum of **8**. The glycosyl unit of **8** was identified as rhamnose by the six ^1^H signals observed in the 1D ^1^H spectrum belonging to this unit and the corresponding six ^13^C signals belonging to this unit observed in the ^13^C CAPT spectrum of **8**, which were assigned by the 2D ^1^H-^13^C HSQC spectrum, the 2D ^1^H-^13^C H2BC spectrum, and the 2D ^1^H-^1^H COSY spectrum of **8**. A cross-peak at δ 5.23/144.9 (H-1’’/C-4) observed in the 2D ^1^H-^13^C HMBC spectrum of **8** confirmed that the rhamnopyranosyl unit was attached to dihydrodehydrodiconiferyl alcohol aglycone at the 4-position. Thus, compound **8** was identified as dihydrodehydrodiconiferyl alcohol 4-*O*-α-rhamnopyranoside, which NMR data can be seen in Table 3. The stereochemistry of this compound was established by circular dichroism (CD) spectroscopy. The CD spectrum of **8** (Figure S59) showed a negative Cotton effect at [θ]_279_, indicating that the configurations of C-7 and C-8 are *R* and *S*, respectively [24,25]. Hence, compound **8** was identified as (7*R*,8*S*)-dihydrodehydrodiconiferyl alcohol 4-*O*-α-rhamnopyranoside (Figure 2). Dihydrodehydrodiconiferyl alcohol 4-*O*-α-rhamnopyranoside has previously been reported from *Pedicularis torta* [26]; however, only incomplete NMR data exist for this compound in the current literature. 

The UV spectrum of compound **12**, which exhibited λ_max_ at 274 and 212 nm (Figure S66), combined with its short HPLC retention time, may be indicative of a polar lactone [27,28], a compound type frequently reported from ferns [29]. The 1D ^1^H NMR spectrum (Figure S29) of **12** showed an 8H spin system at δ 4.22 dd 4.2, 1.3 Hz (H-4), δ 4.07 dt 6.8, 4.3 Hz (H-3), δ 3.74 dd 6.5, 4.2 Hz (H-5), δ 2.80 dd 18.2, 6.8 Hz (H-2A), δ 2.39 dd 18.2, 1.3 Hz (H-2B), and δ 1.12 d 6.5 Hz (H-6), in addition to a methoxy group at δ 3.23 (3-OCH_3_). The 2D ^1^H-^13^C HMBC spectrum (Figure S31) assigned the seven ^13^C signals observed in the 1D ^13^C CAPT spectrum **12**, the 2D ^1^H-^13^C HSQC spectrum, and the 2D ^1^H-^13^C H2BC spectrum (Figure S30) **12**. The 7H spin system observed in the 1D ^1^H NMR spectrum of **12** were assigned by the 2D ^1^H-^13^C HSQC spectrum, the 2D ^1^H-^13^C HSQC-TOCSY spectrum, the 2D ^1^H-^13^C H2BC spectrum, and the 2D ^1^H-^1^H COSY spectrum of **12** (Figures S32–S36). The cross-peaks at δ 2.80/76.0 (H-2A/C-3), δ 2.39/76.0 (H-2B/C-3), δ 4.07/35.0 (H-3/C-2), δ 4.07/88.1 (H-3/C-4), δ 4.22/76.0 (H-4/C-3), δ 4.22/65.6 (H-4/C-5), δ 3.74/88.1 (H-5/C-4), δ 3.74/19.0 (H-5/C-6), and δ 1.12/65.6 (H-6/C-5) observed in the 2D ^1^H-^13^C H2BC spectrum of **12** were particularly useful for the assignments of these signals. The cross-peaks established the position of the lactone carbonyl carbon C-1 at δ 2.80/175.9 (H-2A/C-1), δ 2.39/175.9 (H-2B/C-1), δ 4.07/175.9 (H-3/C1), and δ 4.22/175.9 (H-4/C-1) observed in the 2D ^1^H-^13^C HMBC spectrum of **12**. A cross-peak at δ 3.23/76.0 (3-OCH_3_/C-3) observed in the 2D ^1^H-^13^C HMBC spectrum of **12** confirmed that the methoxy group was attached to the 3-position. Thus, **12** was identified as 3-methoxy-5-hydroxy-4-olide. The stereochemistry of **12** was established by circular dichroism (CD) spectroscopy. The CD spectrum of **12** (Figure S60) showed a positive Cotton effect at [θ]_250_, which indicates *R* and *S* configurations of C-4 and C-5, respectively, whereas the positive Cotton effect at [θ]_208_ indicated an *S* configuration of C-3 [15,21,30]. Thus, **12** was identified as the previously undescribed natural product (3*S*,5*S*,4*R*)-methoxy-hydroxy-olide (Figure 2 and Table 4). A molecular ion [M+H]^+^ at *m*/*z* 161.0809 corresponding to C_7_H_12_O_4_H^+^ (calculated: *m*/*z* 161.0808; Δ = −0.1 ppm) observed in the HRMS (Figure S56) of compound **12** confirmed this identification (Figure 2).

The UV spectrum of **13** (Figure S67) showed a λ_max_ at 296 nm, 266 nm, 220 nm, and 198 nm that corresponds to a saturated γ,δ-dilactone [27]. The 1D ^1^H NMR spectrum (Figure S37) of compound **13** showed the presence of two CH_3_ groups at δ 1.29 d 6.3 Hz (5-CH_3_) and δ 1.13 d 6.6 Hz (H-2’’), one CH_2_ group at δ 2.82 dd 15.2, 11.5 Hz (H-2A) and δ 2.66 dd 15.2, 6.6 Hz (H-2B), in addition to seven CH groups at δ 2.91 dddd 11.5, 8.0, 6.6, and 1.4 Hz (H-3), δ 4.04 dd 8.7, 8.0 Hz (H-4), δ 4.39 dd 8.7, 6.3 Hz (H-5), δ 3.21 dd 5.3, 1.4 Hz (H-2’), δ 4.82 d 5.3 Hz (H-3’), δ 4.28 d 5.3 Hz (H-4’), and δ 3.78 dd 6.6, 3.7 Hz (H-1’’). The corresponding ^13^C signals were observed at δ 18.19 (5-CH_3_), δ 19.02 (C-2’’), δ 32.73 (C-2), δ 38.23 (C-3), δ 80.22 (C-4), δ 73.19 (C-5), δ 51.87 (C-2’), δ 78.56 (C-3’), δ 86.97 (C-4’), and δ 65.51 (C-1’’) in the 1D ^13^CAPT NMR spectrum of compound **13** (Figure S38), in addition to two ester carbonyls at δ 171.25 (C-1) and δ 176.85 (C-1’), which describe the presence of two different lactone rings attached through a C-C bond (Figure 2). The 2D ^1^H-^13^C HSQC-TOCSY spectrum of compound **13** revealed that the 15 hydrogens observed in the 1D ^1^H NMR spectrum all belonged to the same spin system. These twelve ^13^C signals observed in the 1D ^13^C CAPT spectrum of **13** were assigned by the 2D ^1^H-^13^C HMBC spectrum, the 2D ^1^H-^13^C HSQC spectrum, and the 2D ^1^H-^13^C H2BC spectrum of **13**. The ^1^H spin systems observed in the 1D ^1^H NMR spectrum of **13** were assigned by the 2D ^1^H-^13^C HSQC spectrum, the 2D ^1^H-^13^C HSQC-TOCSY spectrum, the 2D ^1^H-^13^C H2BC spectrum, and the 2D ^1^H-^1^H COSY spectrum (Figures S40–S42). The cross-peaks at δ 2.82/38.2 (H-2A/C-3), δ 2.66/38.2 (H-2B/C-3), δ 2.91/32.7 (H-3/C-2), δ 2.91/80.2 (H-3/C-4), δ 4.04/73.2 (H-4/C-5), δ 4.04/38.2 (H-4/C-3), δ 4.39/80.2 (H-5/C-4), δ 4.39/18.2 (H-5/C-6), and δ 1.29/73.2 (H-6/C-5) observed in the 2D ^1^H-^13^C H2BC spectrum of **13** belonging to the δ-lactone ring and the cross-peaks at δ 3.21/78.6 (H-2’/C-3’), δ 4.82/51.9 (H-3’/C-2’), δ 4.28/65.5 (H-4’/C-1’’), δ 3.78/87.0 (H-1’’/C-4’), δ 3.78/19.0 (H-1’’/C-2’’), and δ 1.13/65.5 (H-2’’/C-1’’) observed in the 2D ^1^H-^13^C H2BC spectrum of **13** belonging to the γ-lactone ring were particularly useful for complete assignment of the non-quaternary carbons of **13**. The ester carbonyls of the γ-lactone ring and the δ-lactone ring were assigned by the observed cross-peaks in the 2D ^1^H-^13^C HMBC spectrum (Figure S39) of **13**. The cross-peaks at δ 4.04/51.9 (H-4/C-2’), δ 2.82/51.9 (H-2A/C-2’), δ 2.66/51.9 (H-2B/C-2’), δ 2.91/51.9 (H-3/C-2’), δ 2.91/176.9 (H-3/C-1’), δ 2.91/78.6 (H-3/C-3’), δ 3.21/32.7 (H-2’/C-2), δ 3.21/38.2 (H-2’/C-3), and δ 3.21/80.2 (H-2’/C-4) observed in the 2D ^1^H-^13^C HMBC spectrum of **13** confirmed the linkage between the γ-lactone ring and the δ-lactone ring from C-3 to C-2’. This was further confirmed by the observation of the cross-peak at δ 2.91/3.21 (H-3/H-2’) observed in the 2D ^1^H-^1^H COSY spectrum of **13** (Figure S43). The cross-peaks at δ 1.13/87.0 (H-2’’/C-4’), δ 4.28/19.02 (H-4’/C-2’’), and δ 3.78/87.0 (H-1’’/C-4’) confirmed that the 1’’-hydroxyethyl substituent was attached to C-4’. Thus, compound **13** was identified as the previously undescribed bilactone 4-hydroxy-3-(3’-hydroxy-4’-(hydroxyethyl)-oxotetrafuranone-5-methyl tetrahydropyranone. Completely assigned chemical shift values of this previously undescribed bilactone are shown in Table 5. The CD spectrum of compound **13** (Figure S61) shows a negative Cotton effect ([θ]_220_) and a positive Cotton effect ([θ]_255_) in accordance with (4*R*,5*S*,3’*S*,1’’*S*,4’*R*) configuration [15,21,30,31]. Thus, compound **13** was identified as (4*R*,5*S*,3’*S*,1’’*S*,4’*R*)-4-hydroxy-3-(3’-hydroxy-4’-(1-hydroxyethyl)-oxotetrahydrofuran-5-methyltetrahydropyranone (Figure 2). A molecular ion [M+H]^+^ at *m*/*z* 275.1124 corresponding to C_12_H_18_O_7_H^+^ (calculated: *m*/*z* 275.1125; Δ = 0.5 ppm) observed in the HRMS (Figure S57) of compound **13** confirmed this identification.

Compound **15** showed a UV spectrum of a saturated δ-lactone with λ_max_ at 296, 266, and 220 nm (Figure S68) [27]. The 1D ^1^H NMR spectrum (Figure S45) of **15** showed a 9H spin system at δ 5.30 ddd 6.6, 3.4, 1.5 Hz (H-4), δ 6.87 dd 9.9, 3.4 Hz (H-3), δ 4.60 p 6.6 Hz (H-5), δ 6.11 dd 9.9, 1.5 Hz (H-2), and δ 1.30 d 6.6 Hz (H-6), acylated with a 5’-hydroxy-oxohexanoyl group displaying signals at δ 2.54 (H-2’), 2.86 (H-3’), δ 4.04 q 6.9 Hz (H-5’), and δ 1.16 d 6.9 Hz (H-6’), respectively. The 12 ^13^C signals observed in the 1D ^13^C CAPT spectrum **15** (Figure S46) were assigned by the 2D ^1^H-^13^C HMBC spectrum, the 2D ^1^H-^13^C HSQC spectrum, and the 2D ^1^H-^13^C H2BC spectrum **15** (Fig. 47-50). The 9H spin system observed in the 1D ^1^H NMR spectrum of **15** were assigned by the 2D ^1^H-^13^C HSQC spectrum, the 2D ^1^H-^13^C HSQC-TOCSY spectrum, the 2D ^1^H-^13^C H2BC spectrum, and the 2D ^1^H-^1^H COSY spectrum (Figure S51) of **15**. The cross-peaks at δ 6.11/67.3 (H-2/C-4), δ 6.87/122.3 (H-3/C-2), δ 6.11/143.8 (H-2/C-3), δ 5.30/76.0 (H-4/C-5), δ 4.60/67.3 (H-5/C-4), δ 4.60/17.9 (H-5/C-6), and δ 1.30/76.0 (H-6/C-5). The cross-peaks of the 5’-hydroxy-oxohexanoyl at δ 2.54/32.1 (H-2’/C-3’), δ 2.86/27.3 (H-3’/C-2’), δ 4.04/19.7 (H-5’/C-6’), and δ 4.04/19.7 (H-5’/C-6’) observed in the 2D ^1^H-^13^C H2BC spectrum of **15** (Figure S50) were particularly useful for the assignments of these signals. The cross-peaks established the position of the lactone carbonyl carbon C-1 at δ 6.11/161.9 (H-2/C-1), δ 6.87/161.9 (H-3/C-1), and δ 4.60/161.9 (H-5/C-1) observed in the 2D ^1^H-^13^C HMBC spectrum of **15**. A cross-peak at δ 2.54/171.7 (H-2’/C-1’), δ 2.86/171.7 (H-3’/C-1’), and δ 5.30/171.7 (H-4/C-1’) observed in the 2D ^1^H-^13^C HMBC spectrum of **15** confirmed that the hydroxy oxohexanoyl group is attached in the 4-position. Thus, **15** was identified as 4-*O*-(5-hydroxy-4-oxohexanoyl) osmundalactone. The complete chemical shift values of compound **15** are presented in Table 6 (Figures S45–S52). The CD spectrum of compound **15** (Figure S62) shows a negative band at ([θ]_218_) and a positive band at ([θ]_260_) that corresponds to a 4*R*, 5*S* configuration, respectively [15,21]. A further negative band was observed at ([θ]_295_), which could be ascribed to the *R*-configuration of C-5’ [32]. Thus, compound **15** was identified to be the previously undescribed natural product 4*R*-*O*-(5*S*-hydroxy-4-(5’*R*-hydroxy4-oxohexanoyl) osmundalactone. The sodium-added molecular ion [M+Na]^+^ at *m*/*z* 279.0841 corresponding to C_12_H_16_O_6_Na^+^ (calculated: *m*/*z* 279.0839; Δ = 0.63 ppm) observed in the HRMS (Figure S58) of compound **15** confirmed this identification. Compound **15** represents the first complete assignment of NMR signals of the osmundalactone core structure. The ^1^H NMR chemical shift values are in good agreement with the previously published incompletely assigned ^1^H NMR data of osmundalactone [21], with the exception of the chemical shift value of H-4, which is experiencing a considerable downfield shift because of the effect of acylation of the 4-hydroxyl with 5’R-hydroxy-4-oxohexanoyl [33]. 

The previously reported beneficial properties of *Osmunda regalis* as a traditional medicinal plant encouraged studies to determine the potential cytotoxic activity against leukaemia cells of natural products isolated from this plant source. The cytotoxicity of compounds **2**, **3**, **6**, **8**, **10**, **11**, **12**, **13**, **14**, and **15**, which include the six previously undescribed natural products, were tested towards the acute myeloid leukaemia cell line MOLM13 and compared with a DMSO control which was the solvent used to dilute the pure compounds. Only compounds **11** and **15**, which are γ- and δ-lactones, respectively, exhibited significant cytotoxic activity against the MOLM13 cells, with EC_50_ values of 46.2 ± 0.07 µM (**11**) and 111.9 ± 0.07 µM (**15**) after 72 hours of incubation. These two compounds were further tested towards normal cell lines of rat kidney epithelial (NRK) cells and rat cardiomyoblasts H9c2 cells to reveal if the cytotoxic activity was selective towards cancer cells. However, the cytotoxic activity of 5-hydroxy-2-hexen-4-olide lactone (**11**) did not differ between the cell lines tested, and the EC_50_ values were in all instances similar: 46.2 μM for MOML13, 48.7 μM for H9c2, and 49.6 μM for NRK (Table 7). The 4-*O*-(5-hydroxy-4-oxohexanoyl) osmundalactone (**15**) was only cytotoxic towards the heart and AML cell lines, exhibiting similar EC_50_ values for these cell lines (102.7 and 111.9 μM, respectively). We did not detect reduced metabolic activity in the NRK cells at the highest concentration tested (200 μM), indicating lower cytotoxicity of compound **15** towards this cell line. Monolactones such as γ- and δ-lactones have previously been reported to induce DNA damage in the blood and the central nervous systems of rats [34] and to exhibit pro-apoptotic activity against various cancer cells through inhibition of NF-κB [35,36]. The fact that γ-lactones have been reported to be more cytotoxic against cancer cells than δ-lactones [37] may explain the observed difference in cytotoxic potency between compound **15** and **11**.

## 3. Materials and Methods

### 3.1. Plant Material

Fresh *Osmunda regalis* L. (Osmundaceae) plant material was provided from the Bergen Botanical Garden of the University of Bergen, Norway, in the autumn season (collection date 09/2020, accession number 1996.700). Prior to extraction, the plant material was stored at −25 °C. The water content of the plant material was determined to be 81.9% of the weight.

### 3.2. Extraction of Compound and Partitions with Organic Solvents

Fern leaves from *O. regalis* were removed from their pinnae (2.1 kg) and were extracted with 22 L methanol (Methanol HPLC, Sigma-Aldrich, Saint Louise, MO, USA) for 72 h at room temperature. The extraction yield was 8.6% of the wet weight. Considering that the water content was 81.9%, the dry weight extraction yield was 40.8%. Then, this methanolic extract was filtered through glass wool and concentrated by a rotary evaporator under reduced pressure. The resulting concentrated aqueous extract was partitioned (three times) with petroleum ether (Petroleum ether—ACS reagent, Sigma-Aldrich, Saint Louise, MO, USA) to a final volume of 1.8 L and followed by partition (three times) with ethyl acetate (Ethyl Acetate—ACS reagent ≥ 99.5%, Sigma-Aldrich, Saint Louise, MO, USA) for a final volume of 2.6 L. The residual aqueous phase and the ethyl acetate phase were concentrated by rotavapor to a volume of 300 mL each. 

### 3.3. Amberlite XAD-7 Column Chromatography

The concentrated residual aqueous partition (300 mL) was applied to an Amberlite XAD-7 column (column dimensions 50 × 1000 mm, containing 500g Amberlite® XAD-7, 20–60 mesh, Sigma-Aldrich, Saint Louise, MO, USA). The mobile phase gradient consisted of 4.5 L distilled water, followed by 1 L 50:50 distilled water–methanol, and 8 L Methanol (Methanol HPLC, Sigma-Aldrich, Saint Louise, MO, USA). The flow rate during this separation was 5 mL/min. This gradient gave a total of 14 fractions with volumes of 1 L, which were analysed individually by analytical HPLC. The same procedure was performed with the ethyl acetate phase, obtaining ten fractions, which were also analysed individually by analytical HPLC. 

### 3.4. Sephadex LH-20 Column Chromatography

The fractions from the water phase, which were treated by Amberlite XAD-7 column chromatography, were concentrated to a volume of 20 mL and further separated individually on a Sephadex LH-20 column (column dimensions 50 × 1000 mm, containing 500 g of Sephadex® LH-20, Sigma-Aldrich, Saint Louise, MO, USA) using a gradient of super distilled water and methanol containing 0.1% TFA (Trifluoroacetic acid—for HPLC, ≥99.0%, Sigma-Aldrich, Saint Louise, MO, USA). Water phase (WP) fractions 9 and 8 were added to the column separately. The gradient consisted of 2.5 L Water–Methanol–TFA 80:20:0.1 *v*/*v*/*v*, followed by 2.5 L Water–Methanol–TFA 50:50:0.1 *v*/*v*/*v*; 2.5 L Water–Methanol–TFA 30:70:0.1 *v*/*v*/*v*, and, finally, 2.5 L Methanol–TFA 100:0.1 *v*/*v*. The flow rate during this separation was 5 mL/min, and 46 (from WP fraction 9) and 63 (from WP fraction 8) fractions were obtained for each sample; the collected fractions had a 90 mL volume each. These samples were analysed individually by analytical HPLC.

The same procedure was followed for one ethyl acetate phase combine fraction (EA3-5) and from the ethyl acetate phase fraction 6 (EA6). This separation method gave 54 fractions from EA3-5 and 25 fractions from EA6 with a final volume of 90 mL each.

### 3.5. Preparative HPLC

Individual pure compounds of the fractions from Sephadex LH-20 column chromatography were isolated by preparative HPLC (Thermo Scientific preparative HPLC equipped with a Dionex Ultimate 3000 variable wavelength detector) equipped with a C18 Ascentis column (column dimensions 250 × 20 mm; 5 µm, spherical particles). A gradient of two solvents was used for elution consisting of mobile phase A (super distilled water–TFA 99.9:0.1; *v*/*v*) and mobile phase B (Acetonitrile–TFA 99.9:0.1; *v*/*v*) (Acetonitrile—for HPLC, gradient grade, ≥99.9%, Sigma-Aldrich, Saint Louise, MO, USA). The elution profile consisted of isocratic elution with A–B (90:10 *v*/*v*) for 4 min, followed by a linear gradient from A–B (90:10 *v*/*v*) to A–B (70:30 *v*/*v*) for the next 10 min, isocratic elution with A–B (70:30 *v*/*v*) for the next 20 min, followed by linear gradient from A–B (70:30 *v*/*v*) to A–B (60:40 *v*/*v*) for the next 10 min, followed by isocratic elution with A–B (60:40 *v*/*v*) for the next 20 min.

The flow rate was 15 mL/min. Portions of 750 µL were manually injected into the HPLC column and were manually collected based on peaks appearing in the online chromatogram recorded at 280 nm. Analytical HPLC analysed fractions from preparative HPLC separation. Following this strategy, 0.8 mg of compound **1** from WP9, 5.0 mg of compound **2** from WP8, 2.5 mg of compound **3** from WP8, 5.0 mg of compound **4** from EA3-5, 9.3 mg of compound **5** from EA3-5, 8.7 mg of compound **6** from EA3-5, 9.9 mg of compound **7** from EA3-5, 13.8 mg of compound **8** from EA6, 5.2 mg of compound **9** from EA6, 10.5 mg of compound **10** from EA6, 24.3 mg of compound **11** from EA3-5, 18.5 mg of compound **12** from EA3-5, 8.6 mg of compound **13** from EA3-5, 9.5 mg of compound **14** from EA3-5, 17.1 mg of compound **15** from EA3-5, 2.7 mg of compound **16** from EA3-5, and 8.7 mg of compound **17** from EA3-5 were isolated.

### 3.6. Analytical HPLC

The HPLC instrument (Agilent Technologies 1260 Infinity II) has a multidiode array detector with an absorbance interval from 210–600 nm, an autoinjector, and a 250 × 4.6 mm 5 μm SUPELCO analytical Ascentis^®^ C18 column. This instrument was used to analyse the individual and combined samples following the method previously published by Nguyen et al. [38]. Two solvents were used for elution, which were mobile phase A (water–TFA 99.9:0.1; *v*/*v*) and mobile phase B (acetonitrile–TFA 99.9:0.1; *v*/*v*), with a flow rate of 1 mL/min; and aliquots of 20 µL were injected. 

The elution profile began with initial 90% A and 10% B conditions. A gradient elution followed this for 10 minutes at 14% B, then an isocratic elution from 10 to 14 min. The subsequent gradient conditions were as follows: 16% B at 18 min, 18% B at 22 min, 23% B at 26 min, 28% B at 31 min, and 40% B at 32 min. This was followed by an isocratic elution from 32 to 40 minutes, gradient elution from 40 to 43 min at 10% B, and a final isocratic elution from 43 to 46 min at 10% B.

### 3.7. Spectrometry

Compounds **2**, **3**, **6,** and **15**’s mass spectra were recorded using an HRMS JEOL AccuTOF™ JMS T100LC instrument fitted with an electrospray ion source operated in positive mode at a resolving power of approximately 6000 FWHM. The spectrum was recorded over the mass range of 50–2000 *m*/*z*. The samples were analysed as solutions in methanol and introduced to the ESI spray chamber by weakly acidified (0.01% HCOOH) acetonitrile (Acetonitrile—for HPLC, gradient grade, ≥99.9%, Sigma-Aldrich, Saint Louise, MO, USA) used as a spray reagent. Ultra-performance liquid chromatography coupled with high-resolution mass spectrometry (UPLC-HRMS) was also used for the exact mass determination of compounds **12** and **13**. An iClass UPLC (Waters) equipped with a C18 BEH column (1.7 μm, 2.1 × 50 mm, Waters) was used for introducing the samples to the mass spectrometer. A gradient of A) 0.2% formic acid and B) acetonitrile was used as follows (% B in A): 1 (isocratic for 0.5 min), from 1 to 90 (2 min). The mass spectrometer (timsTOF, Bruker) was used in ESI+-mode with an ionisation at 2 kV and with a full scan 100–2000 Da, with resolution R=50 000 (FWHM) at 1000 Da. Exactness was at RMS < 1 ppm.

UV–Vis absorption spectra were recorded online during the analytical HPLC analysis over the 210-600 nm wavelength range in steps of 2 nm.

Circular Dichroism (CD) spectra were recorded at 20 °C with a nitrogen atmosphere in a Jasco J-810 spectropolarimeter equipped with a Peltier temperature control unit. This instrument was used to analyse compounds **8**, **12**, **13,** and **15** (18 mM, 25 mM, 12 mM, and 23 mM, respectively) solubilised in 100% methanol. The spectra obtained were the average of six scans, and a buffer scan with 100% methanol (Methanol—for HPLC, ≥99.9%, Sigma-Aldrich, Saint Louise, MO, USA) was subtracted from the spectra. The spectra were scanned from 185 to 400 nm. A 1 mm path-length cell was used during this analysis.

NMR samples were prepared by dissolving the isolated compound in deuterated dimethylsulfoxide (DMSO-D_6_; 99,96 atom % D, Sigma-Aldrich, Saint Louis, MO, USA). The 1D ^1^H, 1D ^1^H selective TOCSY, 1D ^13^CAPT and the 2D ^1^H-^13^C HMBC, the 2D ^1^H-^13^C HSQC, the 2D ^1^H-^13^C HSQC-TOCSY, the 2D ^1^H-^13^C H2BC, the 2D ^1^H-^1^H COSY, and 2D ^1^H-^1^H ROESY NMR experiments were obtained on a Bruker BioSpin AVANCE III HD 850 MHz equipped with a ^1^H, ^13^C, ^15^N triple resonance cryogenic probe at 298 K.

### 3.8. Cytotoxicity

The pure compounds were diluted to create stock solutions with a final concentration of 20 mM in DMSO (Dimethyl Sulfoxide—for molecular biology, Sigma-Aldrich, Saint Louise, MO, USA). Normal rat kidney epithelial cells (NRK, ATCC no.: CRL-6509) and rat cardiomyoblasts (H9c2, ATCC no.: CRL-1446) were grown in DMEM medium enriched with 10% foetal bovine serum (FBS, Invitrogen, Carlsbad, CA, USA). Once the cells achieved 80% confluence, they were gently detached using trypsin treatment (0.33 mg/mL trypsin for 5 min at 37 °C), centrifuged (160× *g*, 4 min), and then reseeded in fresh medium to reach 25% confluence.

The AML cell line MOLM13 (DSMZ no.: ACC554, [39]) was maintained in RPMI-1640 medium supplemented with 10% FBS and 8 mM L-glutamine (Sigma Life Science, Dorset, UK). The cells were kept in suspension cultures with a density of 150,000 to 700,000 cells/mL. 

The verified cell lines were purchased from the respective suppliers, expanded, and then stored under liquid nitrogen upon usage.

All media were further supplemented with 1 IU/mL penicillin and 1 mg/mL streptomycin (both from Cambrex, Liège, Belgium) and incubated in a humidified environment (37 °C, 5% CO_2_). For the cytotoxicity experiments with compounds 11 to 15, the NRK and H9c2 cells were seeded in 96-well tissue culture plates (4000 cells/well, 0.1 mL) and left overnight to attach before treatments. The MOLM13 cells were seeded in 96-well tissue culture plates at 20,000 cells/well in 0.1 mL on the day of the experiment. 

Compounds dissolved in DMSO were added to the cells, and the plates were incubated for 72 hours before adding the tetrazolium salt WST-1 according to the manufacturer’s instructions (Roche Diagnostics GmbH, Mannheim, Germany). The plates were further incubated for two hours before the signal was recorded at 450 nm with reference at 620 nm. For black subtraction, medium and plant compounds were added to WST-1. This procedure was performed after 24 and 72 h.

After recording WST-1, the cells were next fixed with 2% buffered formaldehyde (pH 7.4) with 0.01 mg/mL of the DNA-specific fluorescent dye, Hoechst 333342. As previously described, the presence of dead (apoptotic or necrotic) cells was verified by differential interference contrast and fluorescence microscopy [40,41]. 

EC_50_ values were determined by a four-parameter regression analysis as described by Viktorsson et al. [42], using SigmaPlot ver. 14.0 (Systat Software Inc., San Jose, CA, USA).

The cell lines were routinely tested for the presence of mycoplasma every six to eight weeks, using MycoAlert™ (Lonza Rockland, Inc., Rockland, ME, USA). No mycoplasma infection was detected during this study.

## 4. Conclusions

The identification of 17 natural products from the aerial parts of *Osmunda regalis* L., a “primitive” fern species, demonstrates that this plant is a valuable source of previously unknown natural products. During the identification process, the complete structure of three previously unidentified flavonoids; kaempferol 3-*O*-(2’’-*O*-(2’’’-α-rhamnopyranosyl)-*β*-glucopyranosyl)-*β*-glucopyranoside, quercetin 3-*O*-(2’’-*O*-(2’’’-α-rhamnopyranosyl)-*β*-glucopyranosyl)-*β*-glucopyranoside, and kaempferol 3-*O*-(2’’-*O*-(2’’’-α-rhamnopyranosyl-6’’’-*O*-(*E*)-caffeoyl-)-*β*-glucopyranosyl)-*β*-glucopyranoside; and three previously undescribed lactones; 3-methoxy-5-hydroxy-4-olide, 4-hydroxy-3-(3’-hydroxy-4’-(hydroxyethyl)-oxotetrafuranone-5-methyl tetrahydropyranone, and the 4-*O*-(5-hydroxy-4-oxohexanoyl) osmundalactone; were determined. The cytotoxic activity of the mono lactones and bilactones in this work confirms that γ-lactones are more cytotoxic against cancer cells than δ-lactones and even bilactones that did not present cytotoxicity against these cell lines correlate well with what could be expected for a unique species that represents one of the last intact survivors of a Jurassic ecosystem.

## Figures and Tables

**Table 1:** ^1^H chemical shift values (ppm) and coupling constants (Hz) of kaempferol 3-O-(2’’-O-(2’’’-α-rhamnopyranosyl)-β-glucopyranosyl)-β-glucopyranoside (compound **2**), quercetin 3-O-(2’’-O-(2’’’-α-rhamnopyranosyl)-β-glucopyranosyl)-β-glucopyranoside (compound **3**), and kaempferol 3-O-(2’’-O-(2’’’-α-rhamnopyranosyl-6’’’-O-(E)-caffeoyl-)-β-glucopyranosyl)-β-glucopyranoside (compound **6**) in DMSO-D_6_ at 298 K.

	Compound 2 δ ^1^H	Compound 3 δ ^1^H	Compound 6 δ ^1^H
2			
3			
4			
5			
6	6.18 *d* 2.0	6.17 *d* 2.1	6.16 *d* 2.1
7			
8	6.42 *d* 2.0	6.38 *d* 2.1	6.37 *d* 2.1
9			
10			
1’			
2’	8.05 ‘*d*’ 8.9	7.49 *d* 2.3	7.98 ‘*d*’ 8.8
3’	6.93 ‘*d*’ 8.9		6.83 ‘*d*’ 8.8
4’			
5’	6.93 ‘*d*’ 8.9	6.89 *d* 8.5	6.83 ‘*d*’ 8.8
6’	8.05 ‘*d*’ 8.9	7.66 *d* 8.5	7.98 ‘*d*’ 8.8
5-OH	12.76 *s*	12.78 *s*	12.74 *s*
7-OH	10.83 *s*	10.81 *s*	10.78 *s*
3’-OH		9.20 *s*	9.02 *s*
4’-OH	10.13 *s*	9.63 *s*	9.52 *s*
3-*O*-*β*-glc			
1’’	5.71 *d* 7.41	5.70 *d* 7.3	5.71 *d* 7.3
2’’	3.70 *dd* 8.5, 7.4	3.73	3.66 *dd* 8.5, 7.3
3’’	3.49 *t* 8.5	3.49	3.50 *t* 8.5
4’’	3.05 *dd* 9.8, 8.5	3.08	3.04 *m*
5’’	3.03 *ddd* 9.8, 5.7, 2.0	3.04	3.04 *m*
6A’’	3.50 *m*	3.51	3.49 *dd* 11.8, 1.9
6B’’	3.19 *dd* 11.3. 5.7	3.20	3.19 *m*
2’’-*O*-*β*-glc			
1’’’	4.97 *d* 7.8	4.94 *d* 7.8	5.02 *d* 7.7
2’’’	3.23 *dd* 9.0, 7.8	3.23	3.27 *dd* 9.1, 7.7
3’’’	3.29 *t* 9.0	3.29	3.31 *t* 9.1
4’’’	3.11 *dd* 9.8, 9.0	3.11	3.22 *dd* 9.8, 9.1
5’’’	3.07 *ddd* 9.8, 5.4, 2.4	3.06	3.35 *ddd* 9.8, 4.8, 2.2
6A’’’	3.67 *dd* 11.9, 2.4	3.64	4.30 *dd* 11.8, 2.2
6B’’’	3.48 *dd* 11.9, 5.4	3.47	4.19 *dd* 11.8, 4.8
2’’’-*O*-α-rha			
1’’’’	5.05 *d* 1.8	5.06 *d* 1.7	5.05 *d* 1.7
2’’’’	3.69 *dd* 3.4, 1.8	3.69	3.69 *dd* 3.4, 1.7
3’’’’	3.53 *dd* 9.3, 3.4	3.54	3.53 *dd* 9.4, 3.4
4’’’’	3.18 *t* 9.3	3.18	3.19 *t* 9.4
5’’’’	4.00 *dd* 9.3, 6.2	3.99	3.98 *dd* 9.4, 6.2
6’’’’	1.17 *d* 6.2	1.17 *d* 6.2	1.17 *d* 6.2
6’’’-*O*-(*E*)-caffeoyl			
1’’’’’			
2’’’’’			6.95 *d* 2.2
3’’’’’			
4’’’’’			
5’’’’’			6.66 *d* 8.2
6’’’’’			6.84 *dd* 8.2, 2.2
7’’’’’			7.36 *d* 15.8
8’’’’			6.16 *d* 15.8
9’’’’’			

**Table 2:** ^13^C chemical shift values (ppm) of kaempferol 3-O-(2’’-O-(2’’’-α-rhamnopyranosyl)-β-glucopyranosyl)-β-glucopyranoside (compound **2**), quercetin 3-O-(2’’-O-(2’’’-α-rhamnopyranosyl)-β-glucopyranosyl)-β-glucopyranoside (compound **3**), and kaempferol 3-O-(2’’-O-(2’’’-α-rhamnopyranosyl-6’’’-O-(E)-caffeoyl-)-β-glucopyranosyl)-β-glucopyranoside (compound **6**) in DMSO-D_6_ at 298 K.

	Compound 2 δ ^13^C	Compound 3 6 δ ^13^C	Compound 6 δ ^13^C
2	155.66	155.71	155.7
3	132.98	133.15	133.2
4	177.55	177.52	177.6
5	161.38	161.40	161.4
6	98.61	98.58	98.6
7	164.04	164.02	164.1
8	93.57	93.38	93.6
9	156.30	156.24	156.3
10	104.01	103.99	104.1
1’	121.12	121.36	121.1
2’	130.90	115.61	130.7
3’	115.38	144.87	115.2
4’	159.94	148.48	160.0
5’	115.38	115.58	115.2
6’	130.90	122.34	130.7
5-OH			
7-OH			
3’-OH			
4’-OH			
3-*O*-*β*-glc			
1’’	98.19	98.26	98.1
2’’	77.83	77.20	78.2
3’’	77.51	77.44	77.3
4’’	70.34	77.30	70.3
5’’	77.35	77.37	77.5
6A’’	60.77	60.89	60.7
6B’’			
2’’-*O*-*β*-glc			
1’’’	100.19	100.35	100.3
2’’’	77.03	76.94	77.1
3’’’	77.82	77.80	77.6
4’’’	70.46	70.42	70.0
5’’’	76.74	76.57	73.7
6A’’’	61.33	61.26	63.4
6B’’’			
2’’’-*O*-α-rha			
1’’’’	100.29	100.25	100.4
2’’’’	70.58	70.55	70.6
3’’’’	70.64	70.61	70.7
4’’’’	72.36	72.38	72.3
5’’’’	68.29	68.29	68.4
6’’’’	17.78	17.80	17.8
6’’’-*O*-(*E*)-caffeoyl			
1’’’’’			125.6
2’’’’’			115.00
3’’’’’			145.5
4’’’’’			148.3
5’’’’’			115.7
6’’’’’			121.3
7’’’’’			145.3
8’’’’			113.8
9’’’’’			166.5

**Table 3:** 1H and 13C chemical shift values (ppm) and coupling constants (Hz) of Dihydrodehydrodiconiferyl alcohol 4-O-α-rhamnopyranoside (compound 8) in DMSO-D6 at 298 K.

	Compound 8 δ ^1^H	Compound 8 δ ^13^C
1		136.58
2	6.99 *d* 2.1	110.61
3		150.10
4		144.85
5	7.04 *d* 8.3	117.90
6	6.85 *dd* 8.3, 2.1	118.04
7	5.46 *d* 6.5	86.56
8	3.42 *dddd* 7.4, 6.5, 5.3, 0.8	53.51
9A	3.71 *dd* 10.8, 5.3	63.20
9B	3.60 *dd* 10.8, 7.4	
1’		135.27
2’	6.68 *d* 1.8	112.56
3’		143.43 (w)
4’		145.53 (s)
5’		128.82 (w)
6’	6.67 *dd* 1.8, 0.8	116.53
7’	3.40 *d* 6.5	60.26
8’	1.68 *m*	34.79
9’	2.52 *m*	31.62
3-OCH_3_	3.73 *s*	55.85
3’-OCH_3_	3.76 *s*	55.75
	4-*O*-*α*-rha	
1’’	5.23 *d* 2.0	99.71
2’’	3.83 *dd* 3.4, 2.0	70.33
3’’	3.63 *dd* 9.4, 3.4	70.47
4’’	3.26 *t* 9.4	71.80
5’’	3.57 *dd* 9.4, 6.2	69.66
6’’	1.08 *d* 6.2	17.92

**Table 4:** 1H and 13C NMR chemical shift values (ppm) and coupling constants (Hz) of the novel lactone 3-methoxy-5-hydroxy-4-olide (compound 12) in DMSO-D6 at 298 K.

	Compound 12 δ ^1^H	Compound 12 δ ^13^C
1		175.86
2A	2.80 *dd* 18.2, 6.8	35.04
2B	2.39 *dd* 18.2, 1.3	
3	4.07 *dt* 6.8, 4.3	76.00
4	4.22 *dd* 4.2, 1.3	88.13
5	3.74 *dd* 6.5, 4.2	65.57
6	1.12 *d* 6.5	18.98
3-OCH_3_	3.23 *s*	55.65

**Table 5:** 1H and 13C NMR chemical shift values (ppm) and coupling constants (Hz) of the novel bilactone 4-hydroxy-3-(3-hydroxy-4-(1-hydroxyethyl)-oxotetrahydrofuran-5-methyltetrahydropyranone (compound 13) in DMSO-D6 at 298 K.

	Compound 13 δ ^1^H	Compound 13 δ ^13^C
1		171.25
2A	2.82 *dd* 15.2, 11.5	32.73
2B	2.66 *dd* 15.2, 6.6	
3	2.91 *dddd* 11.5, 8.0, 6.6, 1.4	38.23
4	4.04 *dd* 8.7, 8.0	80.22
5	4.39 *dd* 8.7, 6.3	73.19
5-CH_3_	1.29 *d* 6.3	18.19
1’		176.85
2’	3.21 *dd* 5.3, 1.4	51.87
3’	4.82 *d* 5.3	78.56
4’	4.28 *d* 3.7	86.97
4’-1’’-hydroxyethyl		
1’’	3.78 *dd* 6.6, 3.7	65.51
2’’	1.13 *d* 6.6	19.02

**Table 6:** 1H and 13C NMR chemical shift values (ppm) and coupling constants (Hz) of the novel lactone 4-O-(5-hydroxy-4-oxohexanoyl) osmundalactone (compound 15) in DMSO-D6 at 298 K.

	Compound 15 δ ^1^H	Compound 15 δ ^13^C
1		161.88
2	6.11 *dd* 9.9, 1.5	122.25
3	6.87 *dd* 9.9, 3.4	143.83
4	5.30 *ddd* 6.6, 3.4, 1.5	67.25
5	4.60 *p* 6.6	76.02
6	1.30 *d* 6.6	17.90
	4-*O*-(5-hydroxy-4-oxohexanoyl)	
1’		171.71
2’	2.54 *m*	27.31
3’	2.86 *m*	32.12
4’		212.85
5’	4.04 *q* 6.9	72.18
6’	1.16 *d* 6.9	19.66

**Table 7:** Cytotoxicity of compounds **11** and **15** against three mammalian cell lines. The compounds were diluted in DMSO. The cells were tested for metabolic activity after 72 h of incubation. The EC_50_ values were determined by non-linear regression from three independent experiments (MOLM-13 and H9c2) described in the methods section. The data from NRK are from three experiments. “-” denotes that no data are available due to low or no toxicity.

	MOLM13 (µM)	H9c2 (µM)	NRK(µM)
**11**	46.2 ± 0.07	48.7 ± 0.18	49.6 ± 0.11
**15**	111.9 ± 0.07	102.7 ± 0.12	-

## Data Availability

The original contributions presented in the study are included in the article/Supplementary Material, further inquiries can be directed to the corresponding author/s.

## Supplementary Materials

The following supporting information can be downloaded at: https://www.mdpi.com/article/10.3390/molecules29174247/s1.
