# Supplementary material for: Cytotoxic Natural Products from the Jurassic Relict Osmunda regalis L
Source: Molecules. 2024 Sep 7;29(17):4247. doi: 10.3390/molecules29174247 (PMC11397566; doi:10.3390/molecules29174247)
Supplement: Supplementary file 1 [file molecules-29-04247-s001.zip › molecules-3181349-supplementary.pdf]

# Cytotoxic Natural Products from the Jurassic Relict *Osmunda regalis* L.

Andrea Estefania Carpinteyro Diaz [1], Lars Herfindal [2], Bjarte Holmelid [1], Cato Brede [3], Heidi Lie Andersen [4], Anni Vedeler [5], and Torgils Fossen [1,*]

1 Department of Chemistry and Centre for Pharmacy, University of Bergen, N-5007 Bergen, Norway; an-drea.diaz@uib.no (A.E.C.D.), bjarte.holmelid@uib.no (B.H.),

2 Department of Clinical Science and Centre for Pharmacy, University of Bergen, N-5009 Bergen, Norway; lars.herfindal@uib.no (L.H.),

3 Department of Medical Biochemistry, Stavanger University Hospital, N-4011 Stavanger, Norway, ca-to.brede@uis.no (C.B.),

4 University Gardens, University of Bergen, Allégt. 41, N-5007 Bergen, Norway, heidi.andersen@uib.no (H.L.A.),

5 Department of Biomedicine, University of Bergen, N-5009 Bergen, Norway, anni.vedeler@uib.no (A.V.);

* Correspondence: torgils.fossen@uib.no (T.F.); Tel.: +47-55-58-34-63

Cover page ………………………………………………………… Page 1
Table of content …………………………………………………….. Page 2
NMR spectra of compound **2** ……………………………………… Page 3-14
NMR spectra of compound **3** ……………………………………… Page 15-24
NMR spectra of compound **6** ……………………………………… Page 25-30
NMR spectra of compound **12** ……………………………………... Page 31-38
NMR spectra of compound **13** ……………………………………… Page 39-46
NMR spectra of compound **15** ……………………………………… Page 47-54
High resolution mass spectrum of compound **2** ……………………… Page 55
High resolution mass spectrum of compound **3** ……………………… Page 56
High resolution mass spectrum of compound **6** ……………………… Page 57
High resolution mass spectrum of compound **12** …………………... Page 58
High resolution mass spectrum of compound **13** …………………... Page 59
High resolution mass spectrum of compound **15** …………………... Page 60
CD spectrum of compound **8** ……………………………………… Page 61
CD spectrum of compound **12** ……………………………………… Page 62
CD spectrum of compound **13** ……………………………………… Page 63
CD spectrum of compound **15** ……………………………………… Page 64
UV spectrum of compound **2** ……………………………………… Page 65
UV spectrum of compound **3** ……………………………………… Page 66
UV spectrum of compound **6** ……………………………………… Page 67
UV spectrum of compound **12** ……………………………………… Page 68
UV spectrum of compound **13** ……………………………………… Page 69
UV spectrum of compound **15** ……………………………………… Page 70
$^{1}$H and $^{13}$C NMR chemical shift values of compound **1** …………… Page 71
$^{1}$H and $^{13}$C NMR chemical shift values of compound **4** …………… Page 72
$^{1}$H and $^{13}$C NMR chemical shift values of compound **5** and **7** ……... Page 73
$^{1}$H and $^{13}$C NMR chemical shift values of compound **9** …………… Page 74
$^{1}$H and $^{13}$C NMR chemical shift values of compound **10** …………... Page 75
$^{1}$H and $^{13}$C NMR chemical shift values of compound **11** …………... Page 76
$^{1}$H and $^{13}$C NMR chemical shift values of compound **14** …………... Page 77
$^{1}$H and $^{13}$C NMR chemical shift values of compound **16** …………... Page 78
$^{1}$H and $^{13}$C NMR chemical shift values of compound **17** …………... Page 79

**Figure S1. 1D $^{1}$H NMR spectrum of kaempferol 3-*O*-(2''-*O*-*β*-glucopyranosyl-(2'''-*O*-*α*-rhamnopyranosyl))-*β*-glucopyranoside (2)**

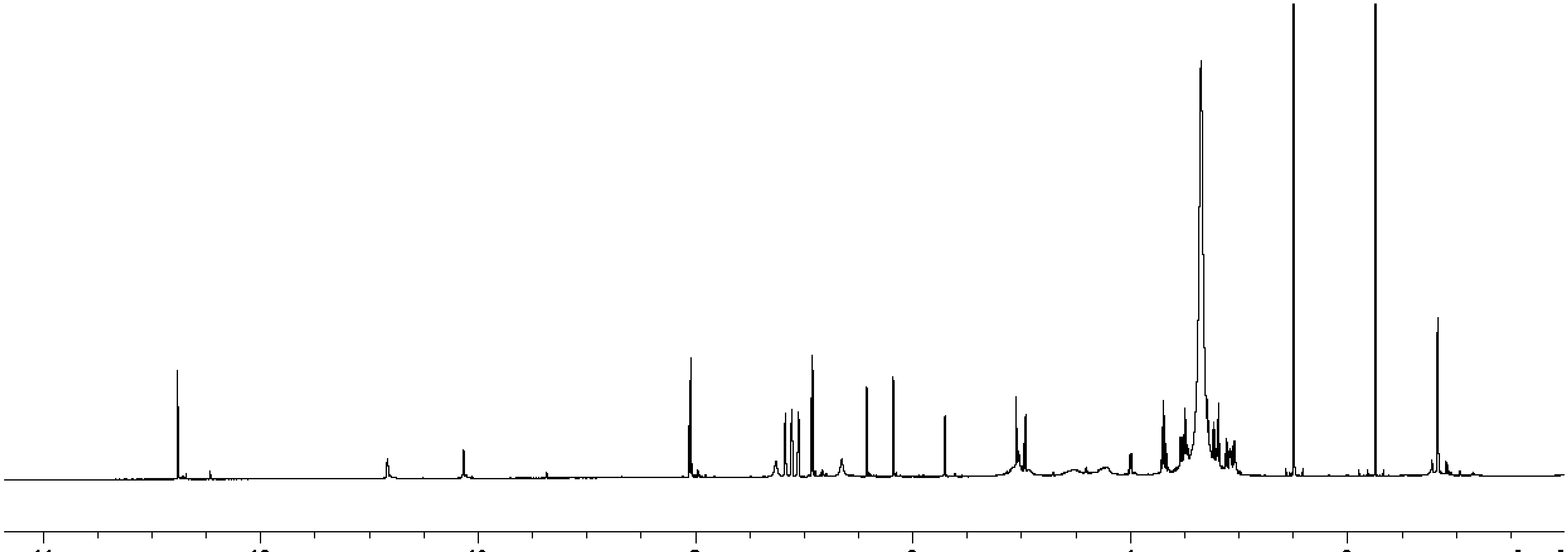

**Figure S2. 1D $^1$H selective TOCSY NMR spectrum of the 3-glucopyranosyl unit of kaempferol 3-*O*-(2''-*O*-*β*-glucopyranosyl-(2'''-*O*-*α*-rhamnopyranosyl))-*β*-glucopyranoside (2)**

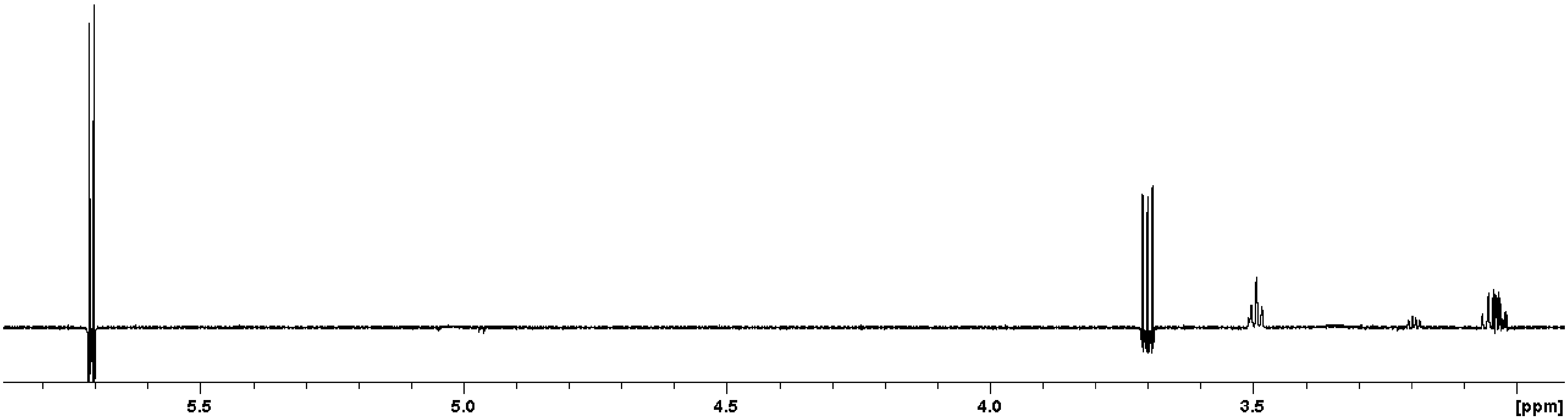

**Figure S3. 1D $^{1}$H selective TOCSY NMR spectrum of the 2’’-glucopyranosyl unit of kaempferol 3-*O*-(2’’-*O*-*β*-glucopyranosyl-(2’’’-*O*-*α*-rhamnopyranosyl))-*β*-glucopyranoside (2)**

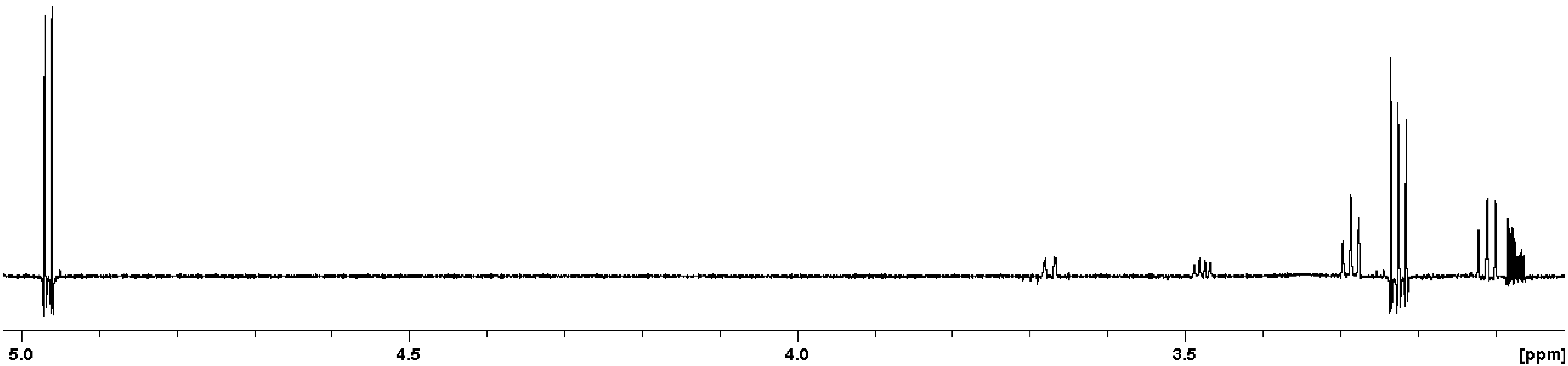

**Figure S4. 1D ¹H selective TOCSY NMR spectrum of the 2’’’-rhamnopyranosyl unit of kaempferol 3-*O*-(2’’-*O*-*β*-glucopyranosyl-(2’’’-*O*-*α*-rhamnopyranosyl))-*β*-glucopyranoside (2)**

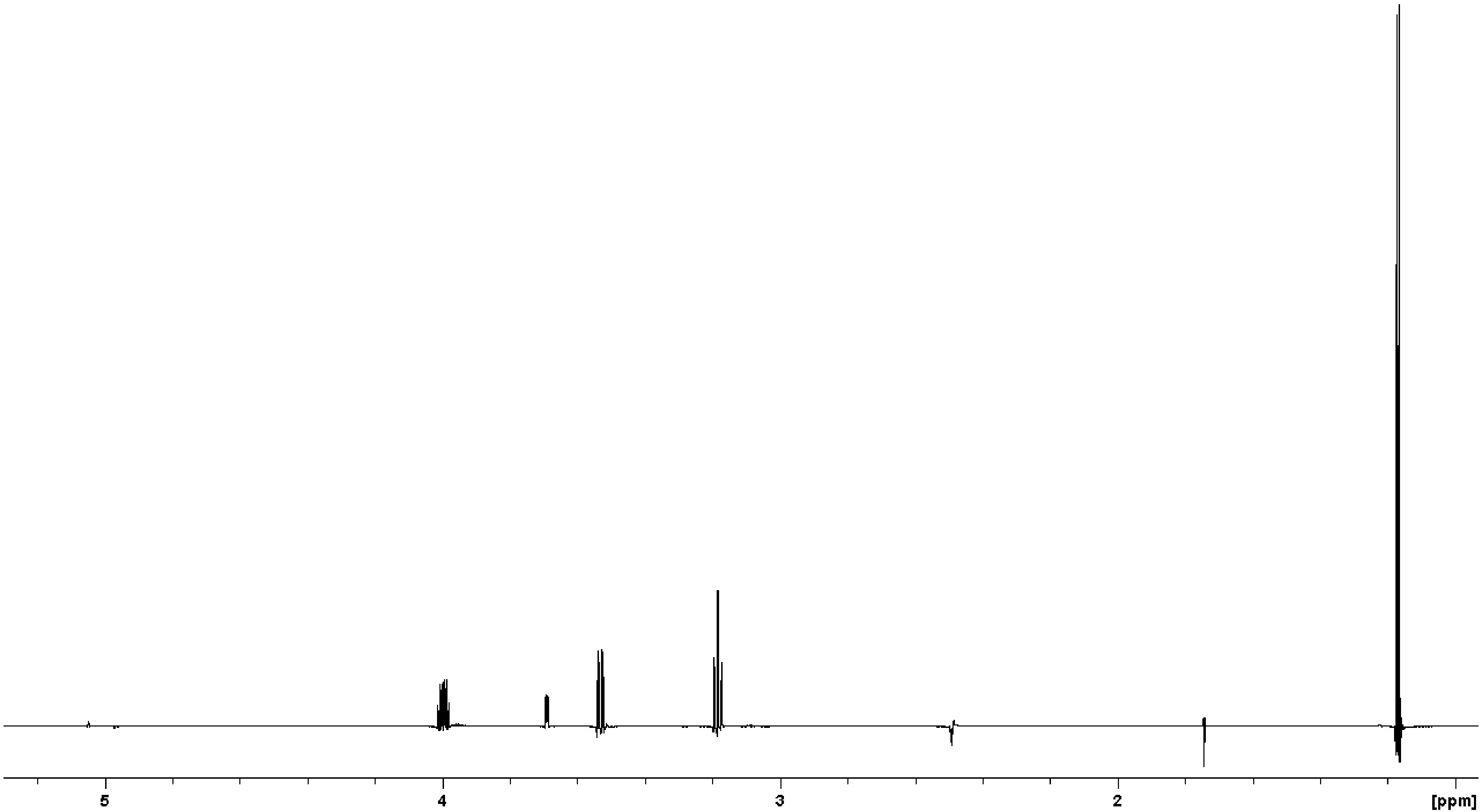

**Figure S5. 1D $^{13}$C CAPT NMR spectrum of kaempferol 3-*O*-(2”-*O*-*β*-glucopyranosyl-(2’”-*O*-*α*-rhamnopyranosyl))-*β*-glucopyranoside (2)**

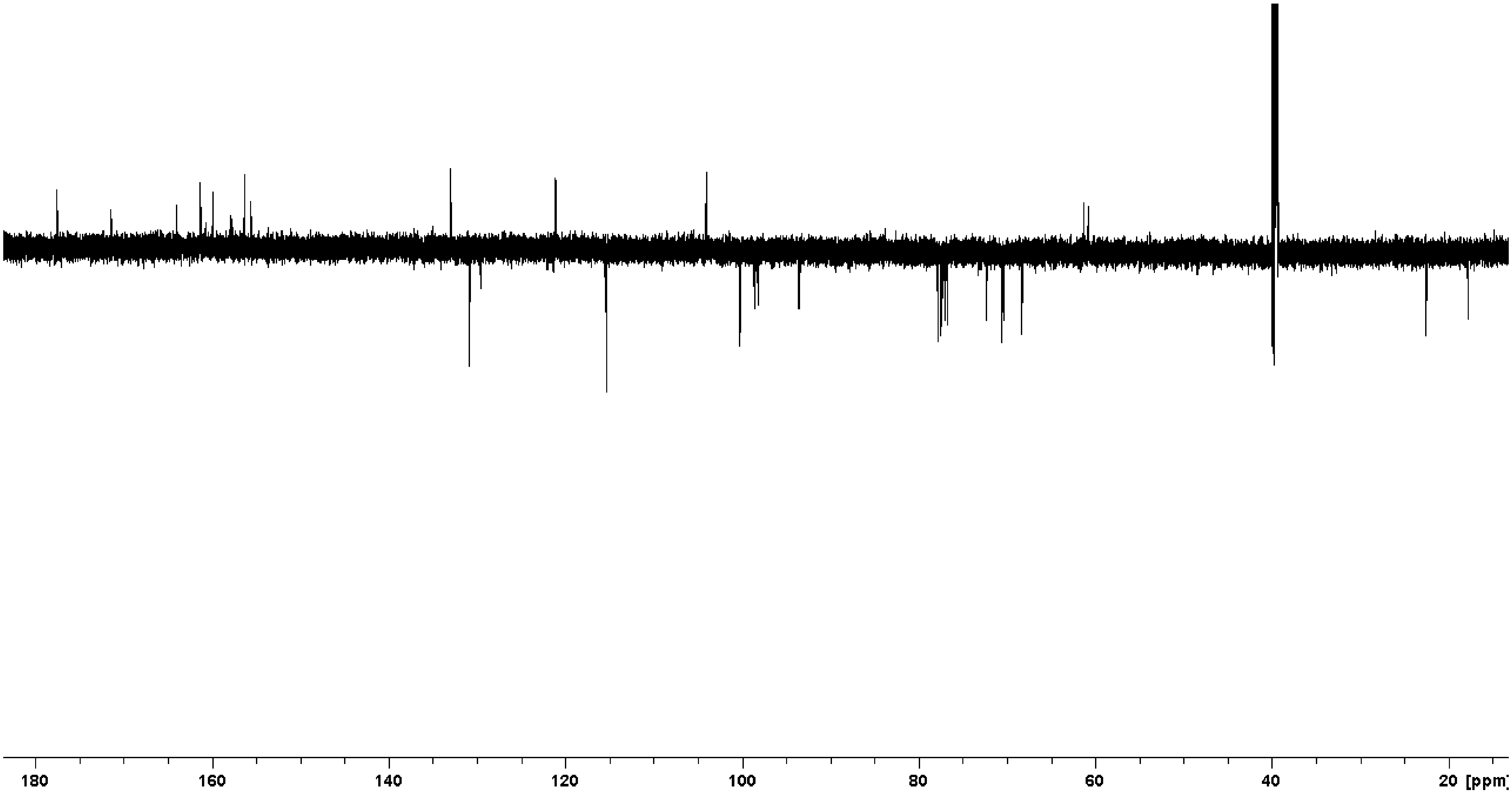

**Figure S6. 2D $^{1}H$-$^{13}C$ HMBC NMR spectrum of kaempferol 3-*O*-(2''-*O*-*β*-glucopyranosyl-(2'''-*O*-*α*-rhamnopyranosyl))-*β*-glucopyranoside (2)**

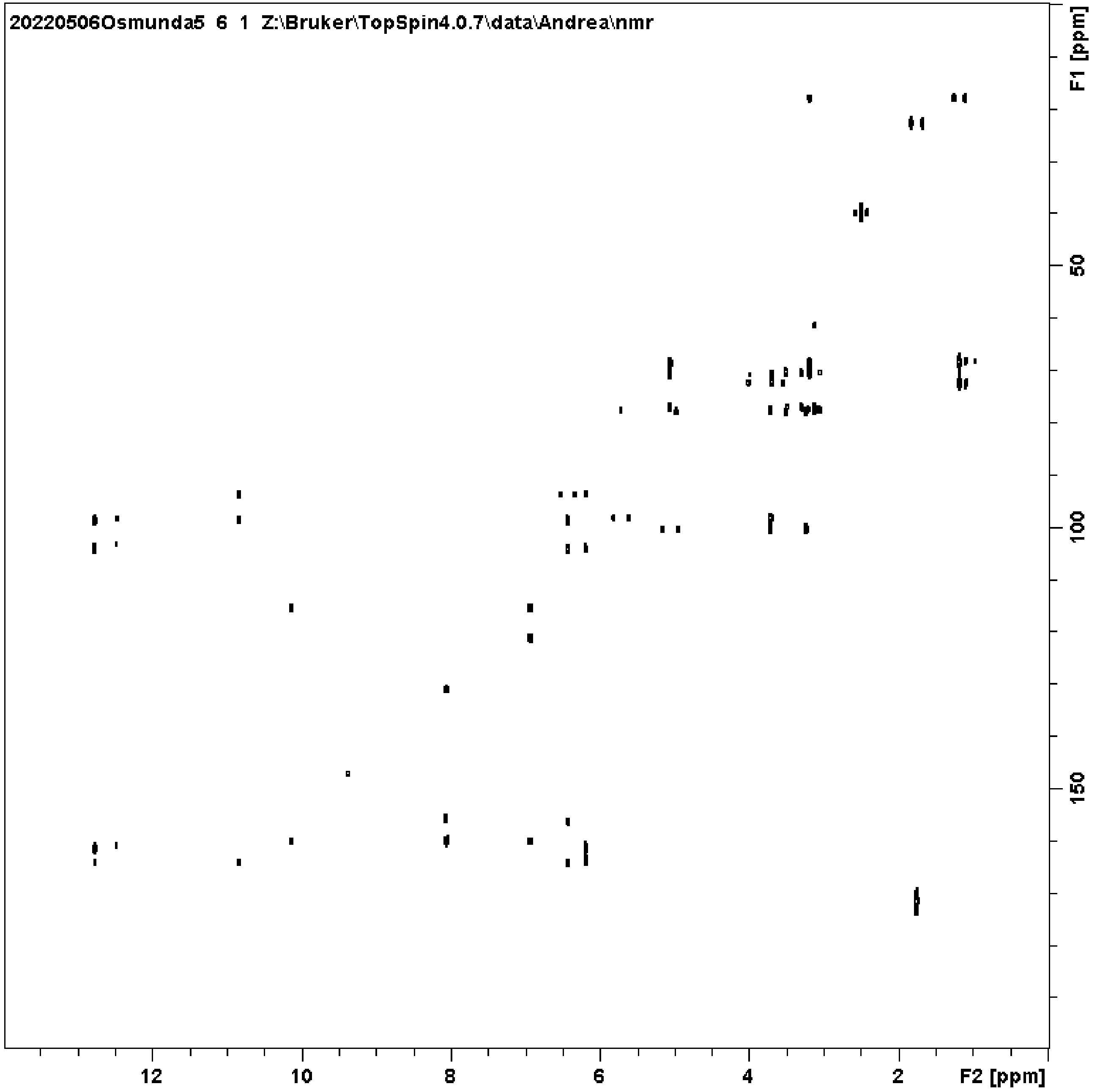


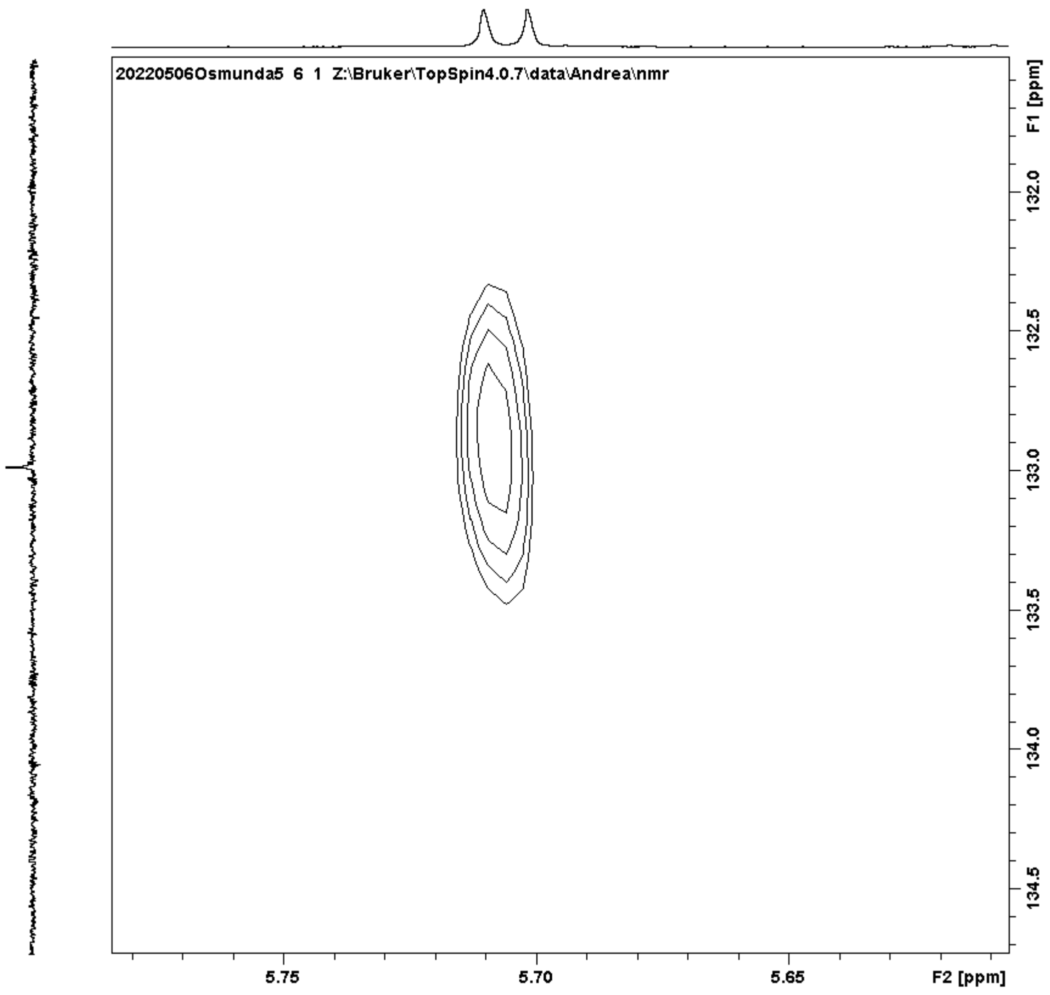

**Figure S7. 2D $^{1}$H-$^{13}$C HSQC NMR spectrum of kaempferol 3-*O*-(2''-*O*-*β*-glucopyranosyl-(2'''-*O*-*α*-rhamnopyranosyl))-*β*-glucopyranoside (2)**

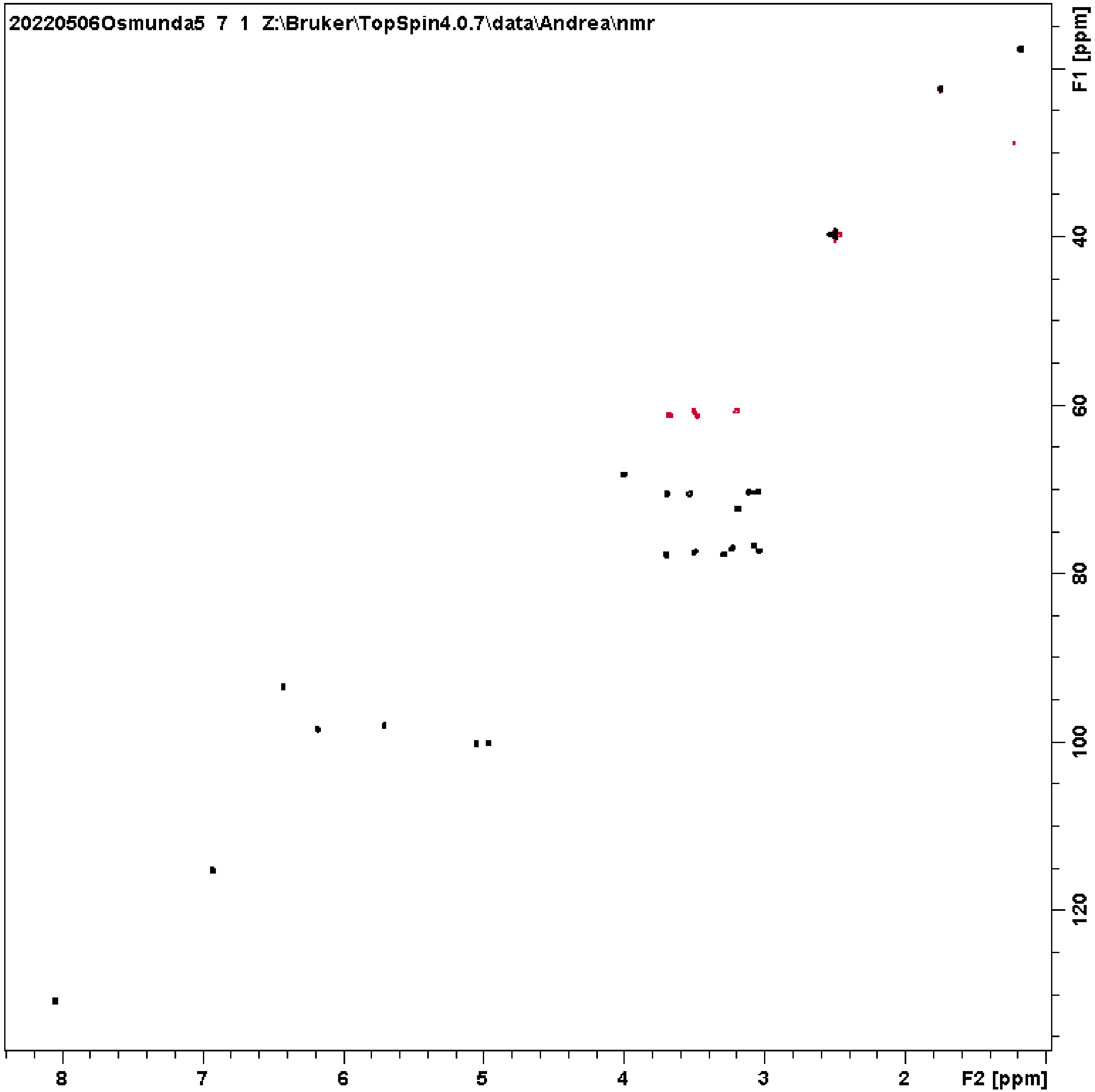

**Figure S8. 2D $^{1}$H-$^{13}$C HSQC-TOCSY NMR spectrum of kaempferol 3-*O*-(2''-*O*-*β*-glucopyranosyl-(2'''-*O*-*α*-rhamnopyranosyl))-*β*-glucopyranoside (2)**

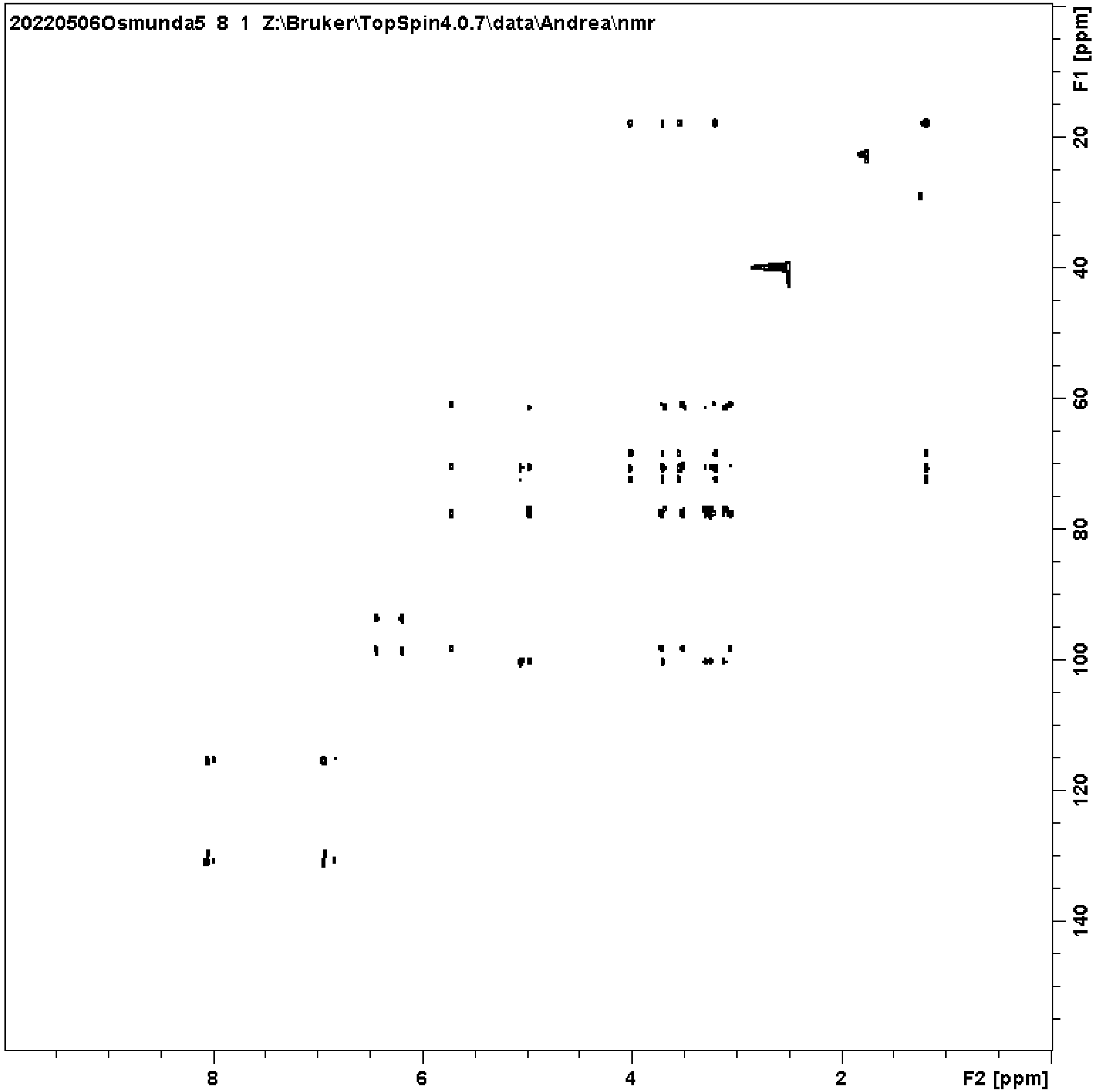

**Figure S9. 2D $^{1}H$-$^{13}C$ H2BC NMR spectrum of kaempferol 3-*O*-(2”-*O*-*β*-glucopyranosyl-(2’”-*O*-*α*-rhamnopyranosyl))-*β*-glucopyranoside (2)**

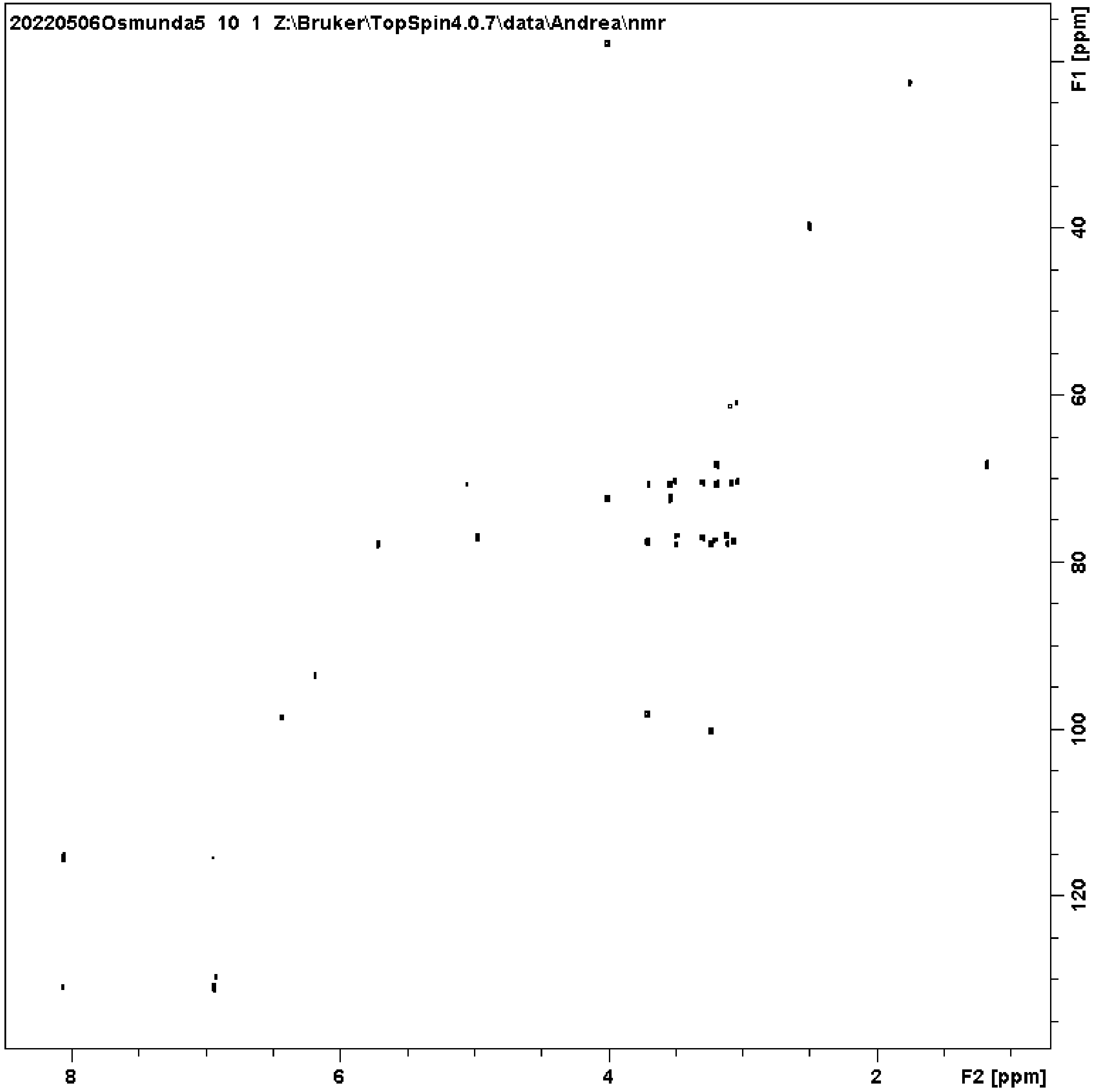

**Figure S10. 2D $^{1}H$-$^{13}C$ Band Selective HMBC NMR spectrum of kaempferol 3-*O*-(2''-*O*-*β*-glucopyranosyl-(2'''-*O*-*α*-rhamnopyranosyl))-*β*-glucopyranoside (2)**

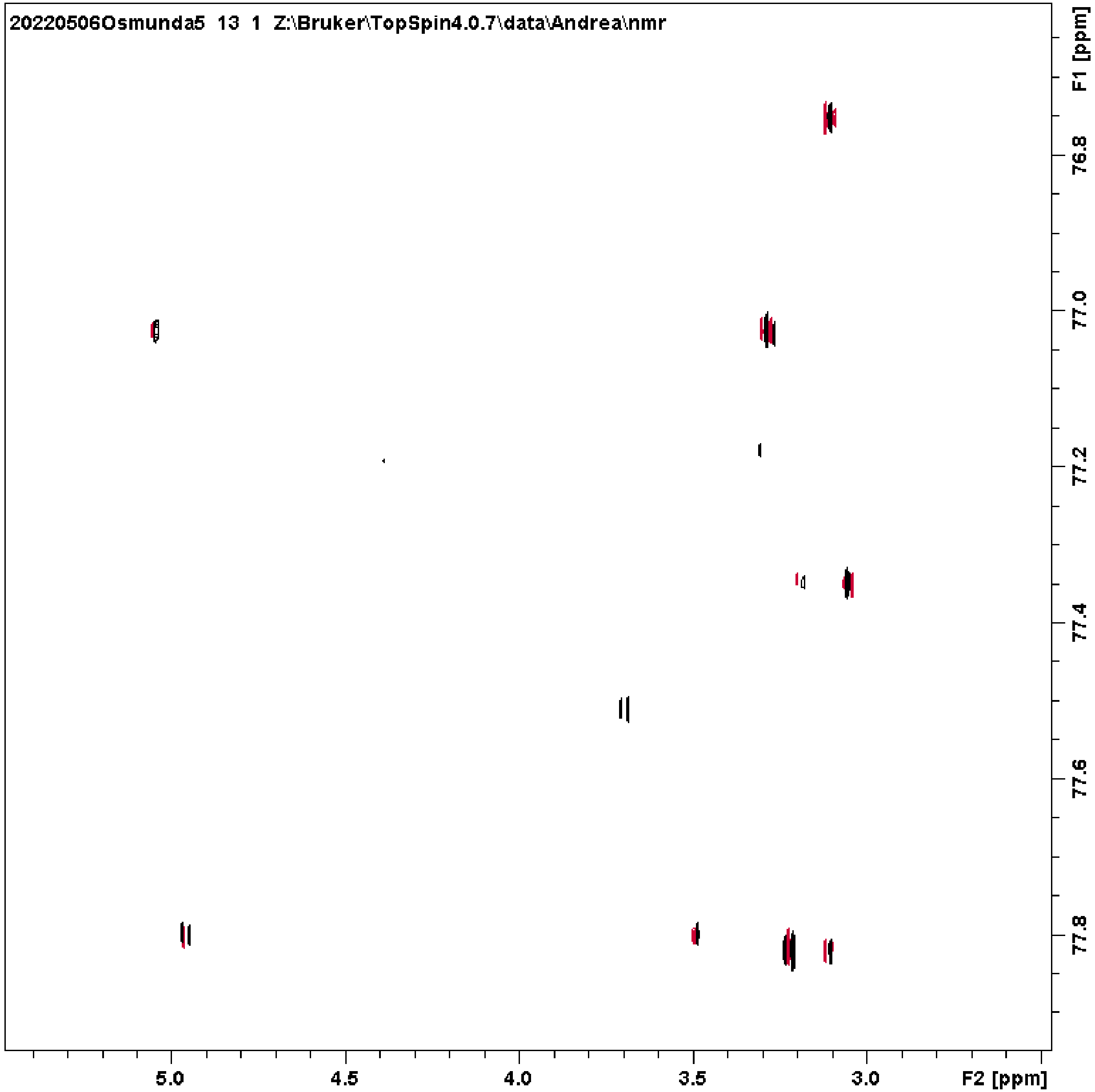

**Figure S11. 2D $^{1}H$-$^{1}H$ COSY NMR spectrum of kaempferol 3-*O*-(2''-*O*-*β*-glucopyranosyl-(2'''-*O*-*α*-rhamnopyranosyl))-*β*-glucopyranoside (2)**

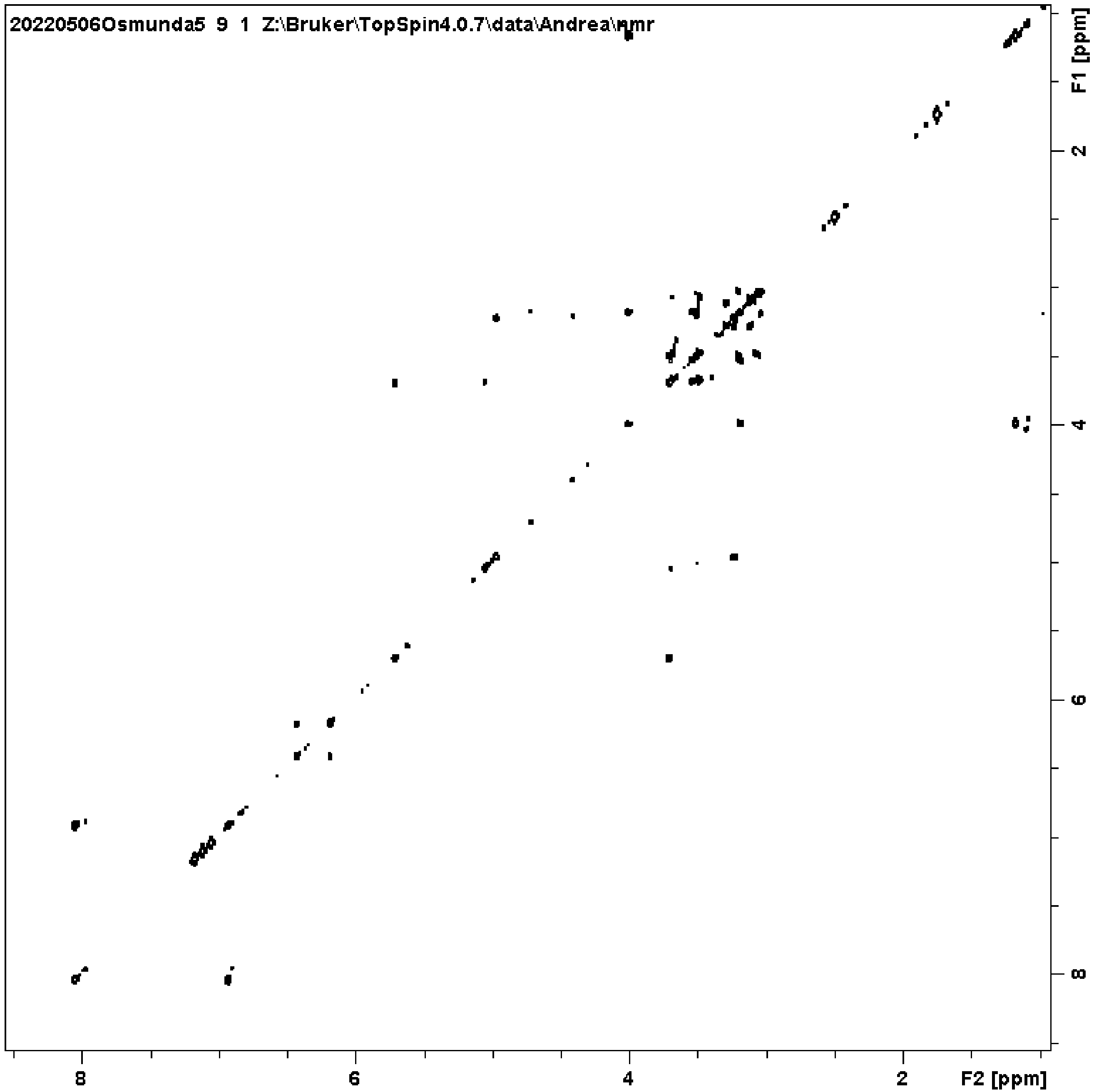

**Figure S12. 2D $^{1}H$-$^{1}H$ ROESY NMR spectrum of kaempferol 3-*O*-(2”-*O*-*β*-glucopyranosyl-(2’”-*O*-*α*-rhamnopyranosyl))-*β*-glucopyranoside (2)**

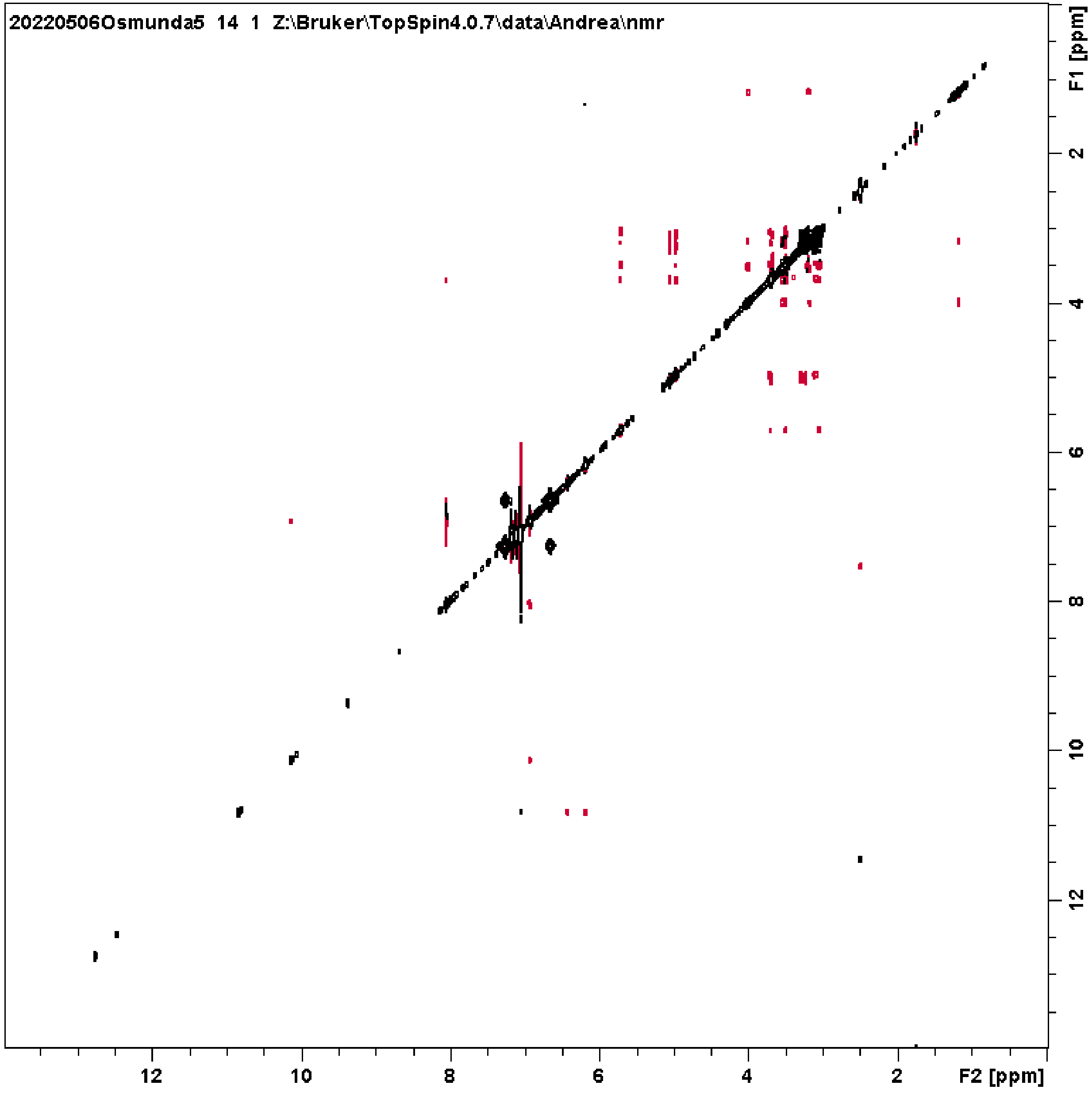

**Figure S13. 1D $^1$H NMR spectrum of quercetin 3-*O*-(2''-*O*-*β*-glucopyranosyl-(2'''-*O*-α-rhamnopyranosyl))-*β*-glucopyranoside (3)**

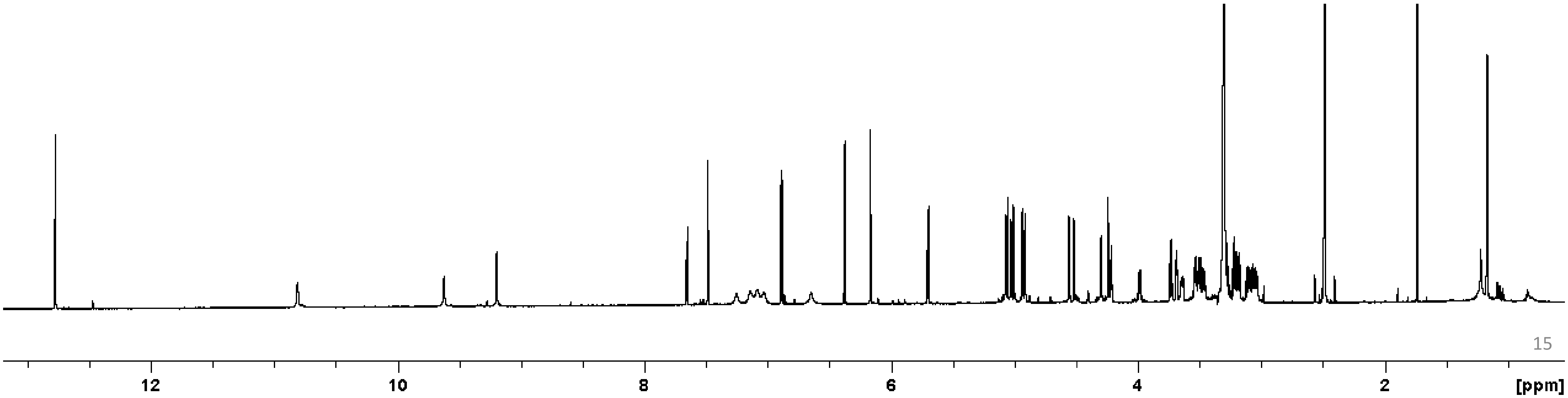

**Figure S14. 1D $^{1}$H selective TOCSY NMR spectrum of the 3-glucopyranosyl unit of quercetin 3-*O*-(2''-*O*-*β*-glucopyranosyl-(2'''-*O*-*α*-rhamnopyranosyl))-*β*-glucopyranoside (3)**

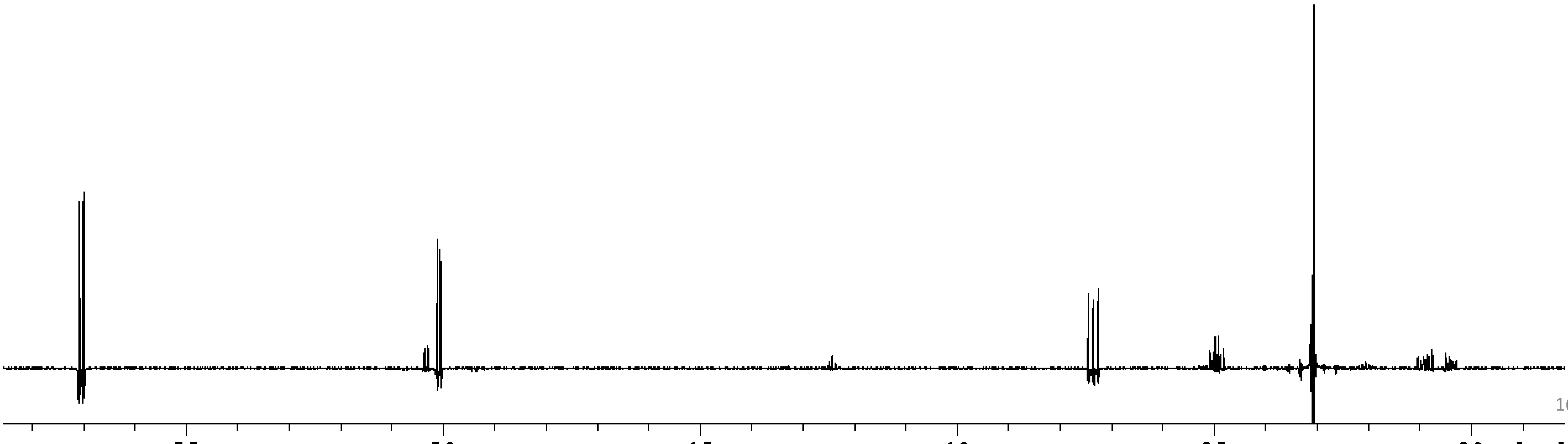

**Figure S15. 1D ¹H selective TOCSY NMR spectrum of the 2''-glucopyranosyl unit of quercetin 3-*O*-(2''-*O*-*β*-glucopyranosyl-(2'''-*O*-α-rhamnopyranosyl))-*β*-glucopyranoside (3)**

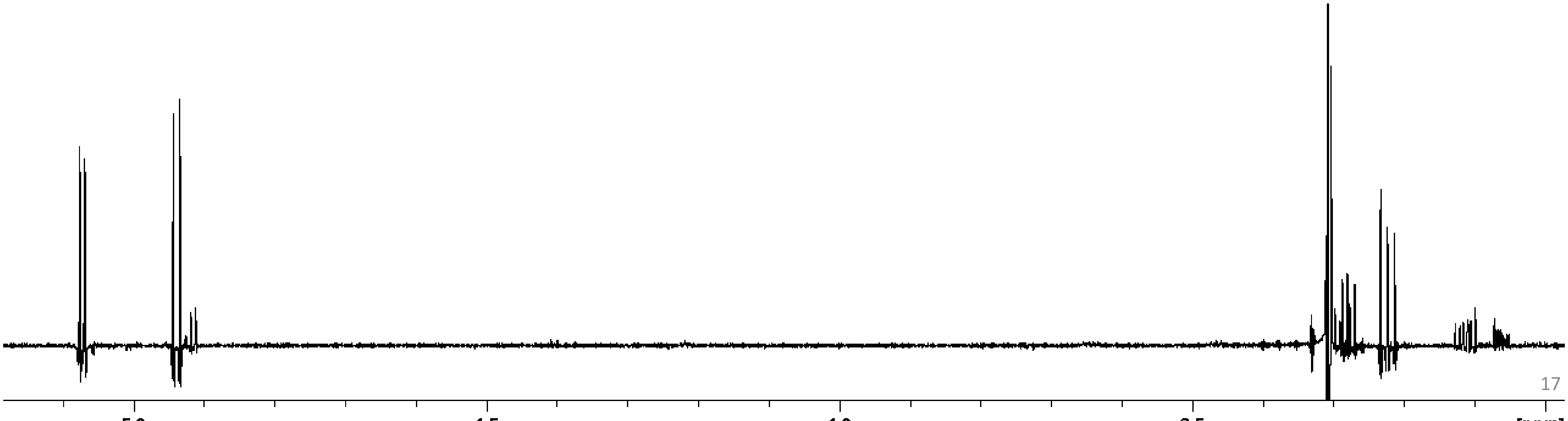

**Figure S16. 1D ¹H selective TOCSY NMR spectrum of the 2’’’-rhamnopyranosyl unit of quercetin 3-*O*-(2’’-*O*-*β*-glucopyranosyl-(2’’’-*O*-*α*-rhamnopyranosyl))-*β*-glucopyranoside (3)**

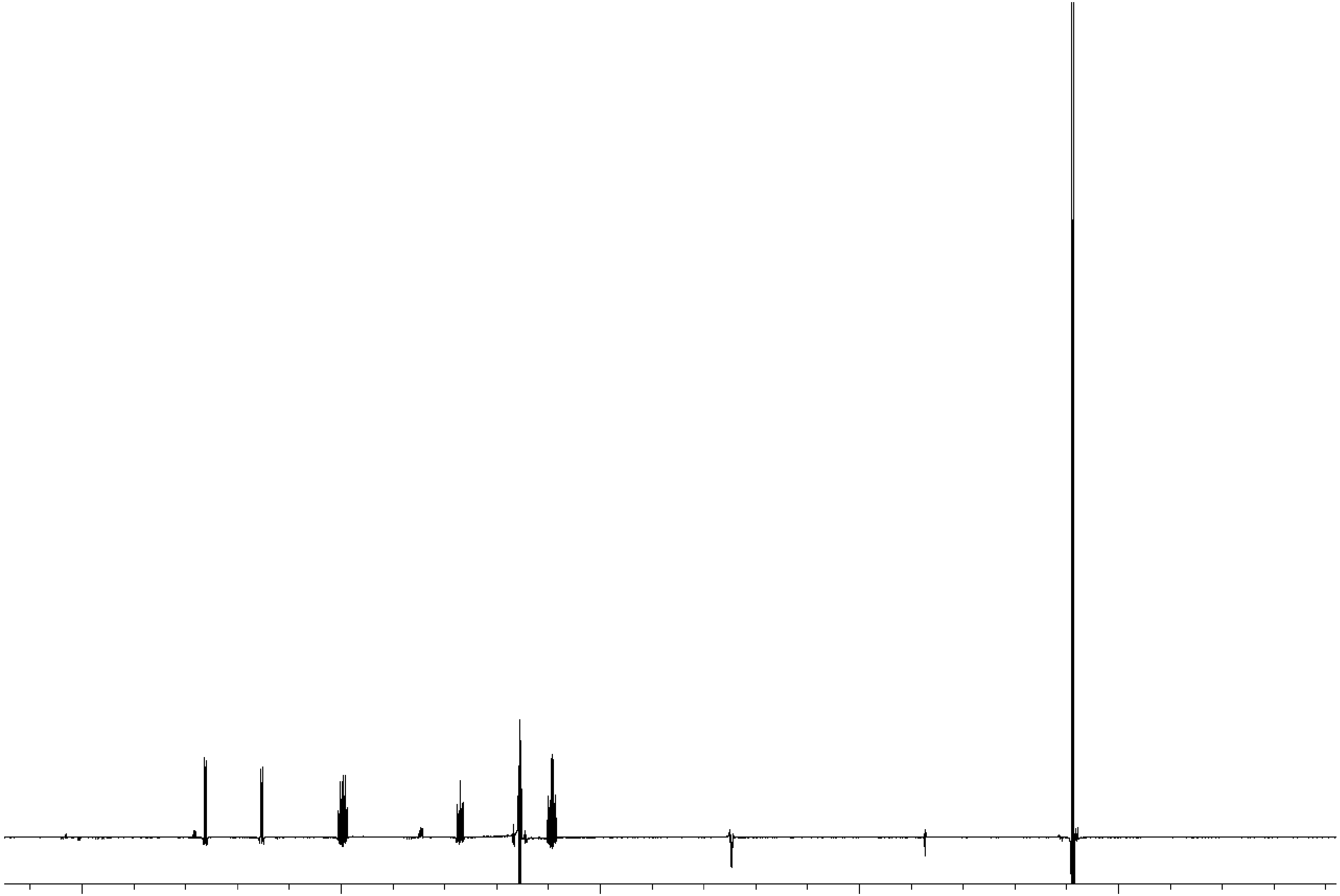

**Figure S17. 1D $^{13}$C CAPT NMR spectrum of quercetin 3-*O*-(2''-*O*-*β*-glucopyranosyl-(2'''-*O*-α-rhamnopyranosyl))-*β*-glucopyranoside (3)**

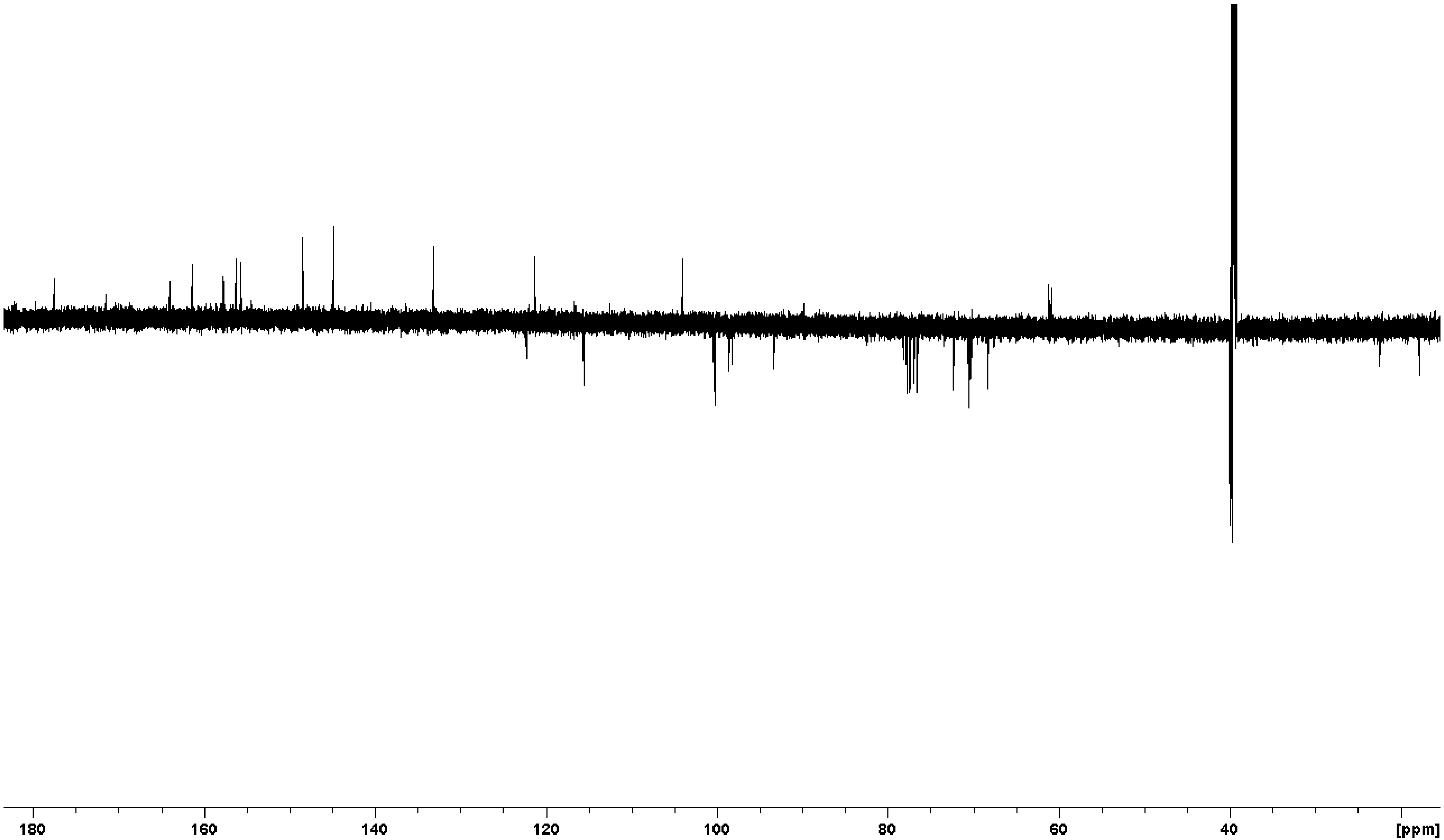

**Figure S18. 2D $^{1}$H-$^{13}$C HMBC NMR spectrum of quercetin 3-*O*-(2''-*O*-*β*-glucopyranosyl-(2'''-*O*-α-rhamnopyranosyl))-*β*-glucopyranoside (3)**

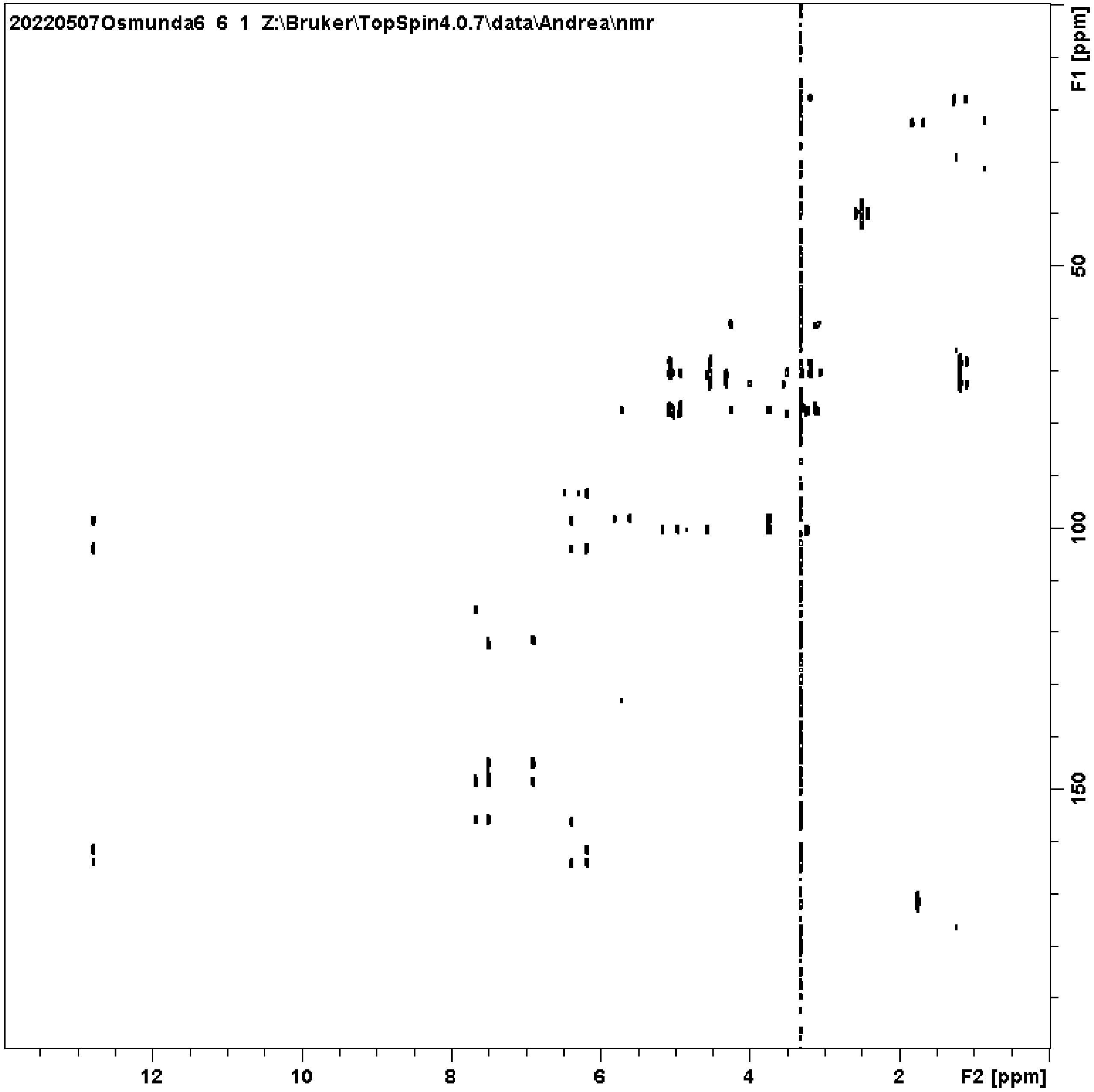

**Figure S19. 2D $^{1}H$-$^{13}C$ HSQC NMR spectrum of quercetin 3-*O*-(2''-*O*-*β*-glucopyranosyl-(2'''-*O*-α-rhamnopyranosyl))-*β*-glucopyranoside (3)**

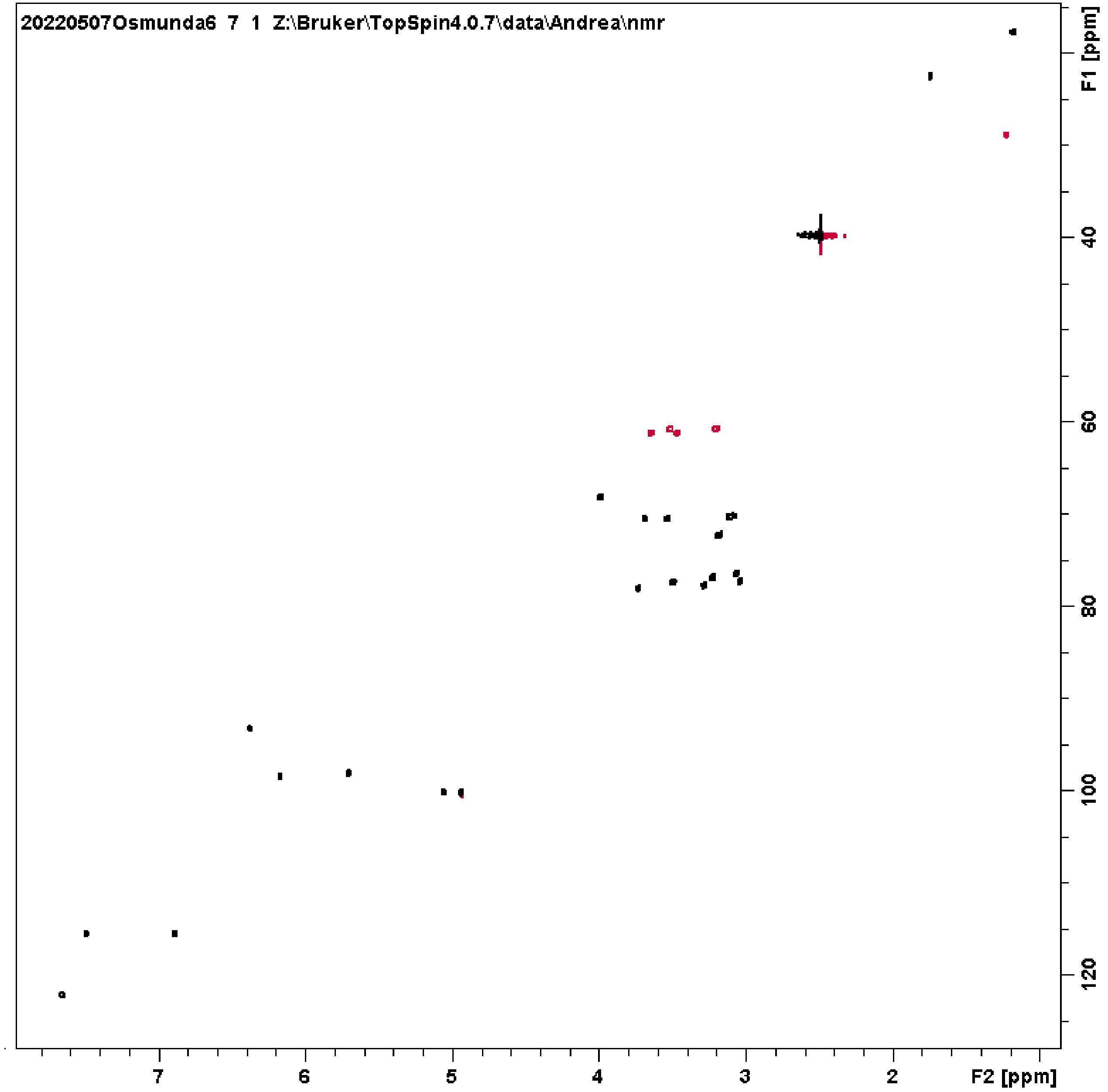

**Figure S20. 2D $^{1}$H-$^{13}$C HSQC-TOCSY NMR spectrum of quercetin 3-*O*-(2''-*O*-*β*-glucopyranosyl-(2'''-*O*-α-rhamnopyranosyl))-*β*-glucopyranoside (3)**

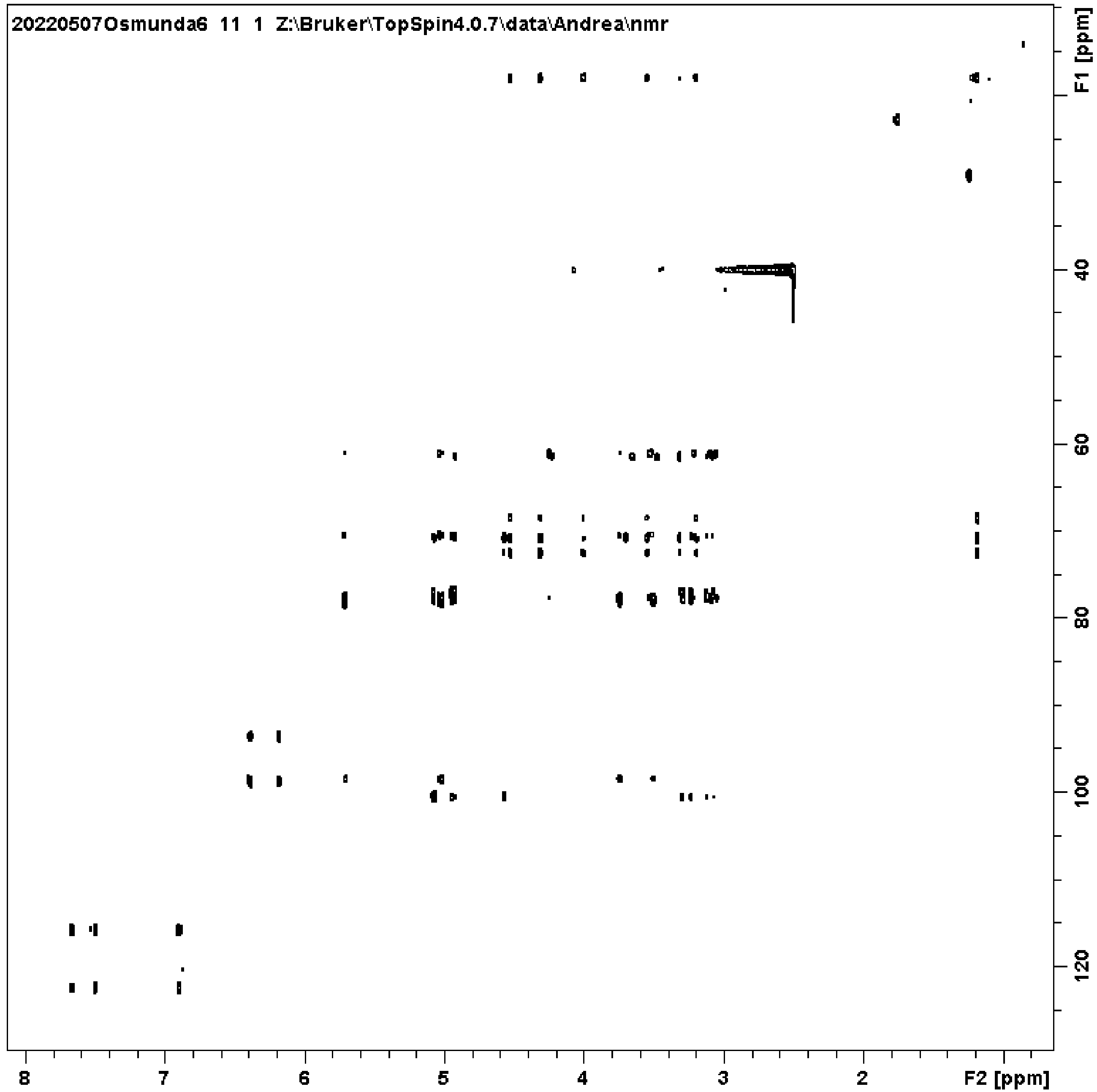

**Figure S21. 2D $^{1}H$-$^{13}C$ H2BC NMR spectrum of quercetin 3-*O*-(2''-*O*-*β*-glucopyranosyl-(2'''-*O*-α-rhamnopyranosyl))-*β*-glucopyranoside (3)**

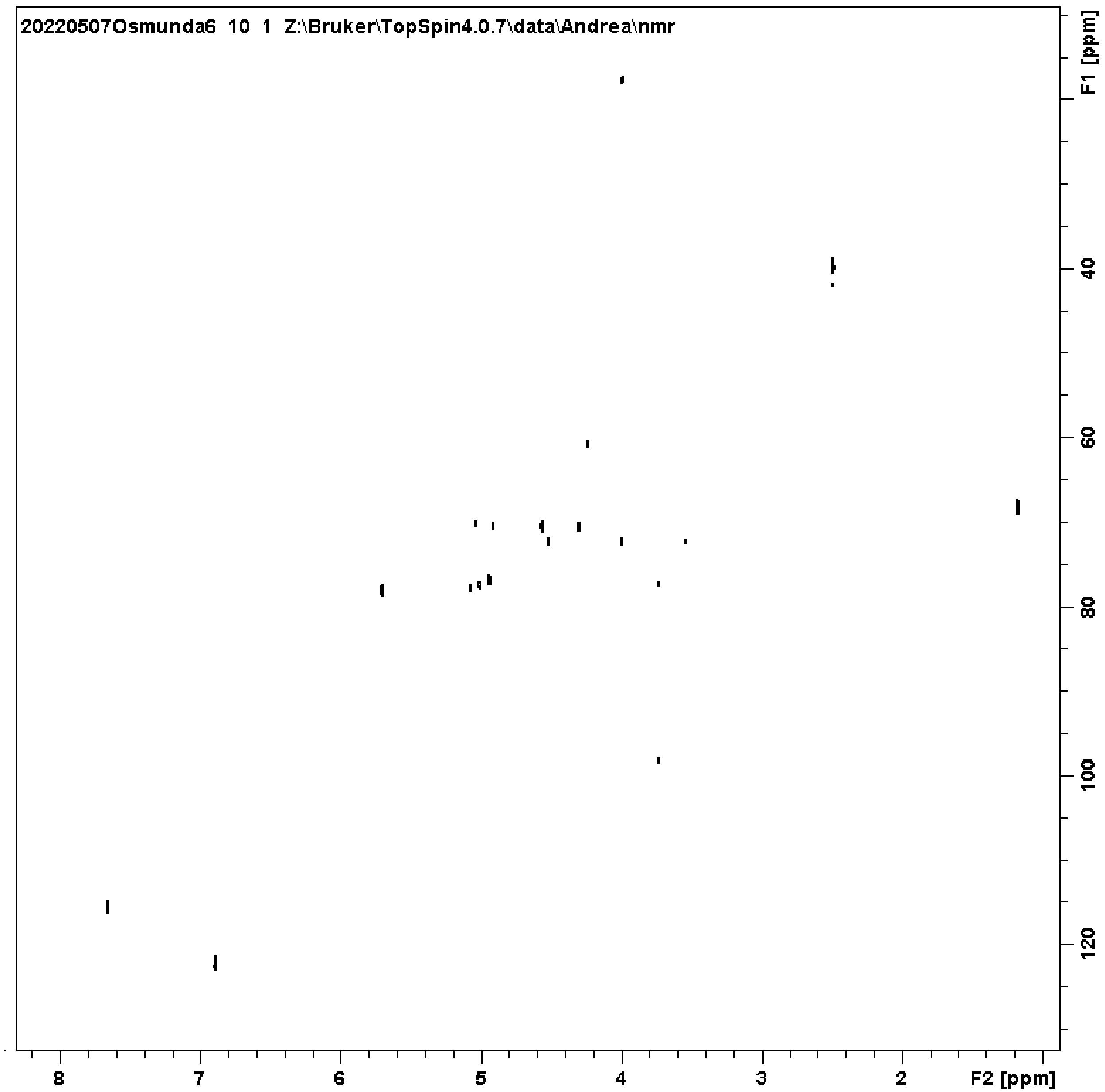

**Figure S22. 2D $^{1}H$-$^{1}H$ COSY NMR spectrum of quercetin 3-*O*-(2''-*O*-*β*-glucopyranosyl-(2'''-*O*-α-rhamnopyranosyl))-*β*-glucopyranoside (3)**

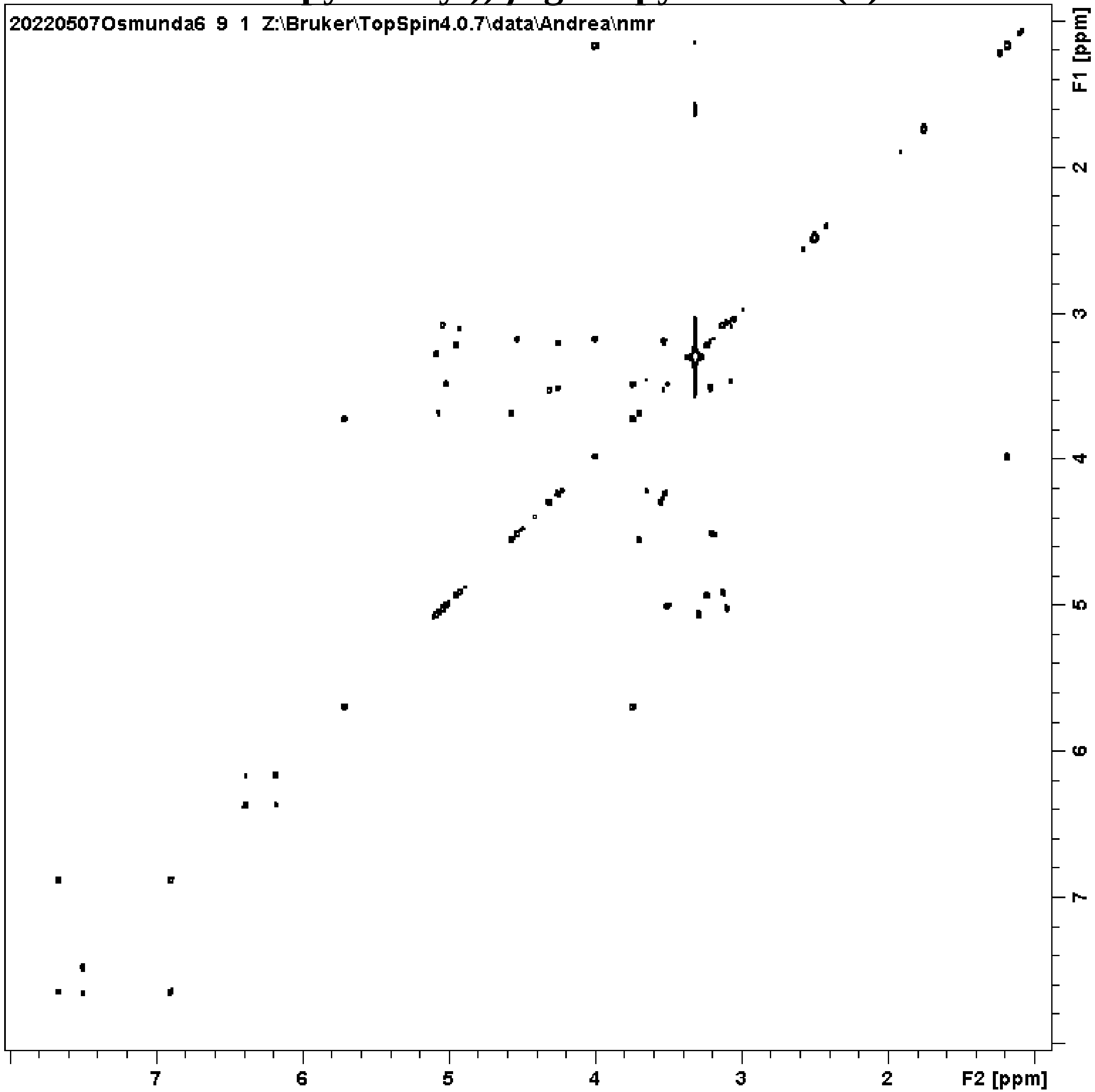

**Figure S23. 1D $^{1}$H NMR spectrum of kaempferol 3-*O*-(2”-*O*-*β*-glucopyranosyl-(2’”-*O*-*α*-rhamnopyranosyl-6’”-*O*-(*E*)-caffeoyl))-*β*-glucopyranoside (6)**

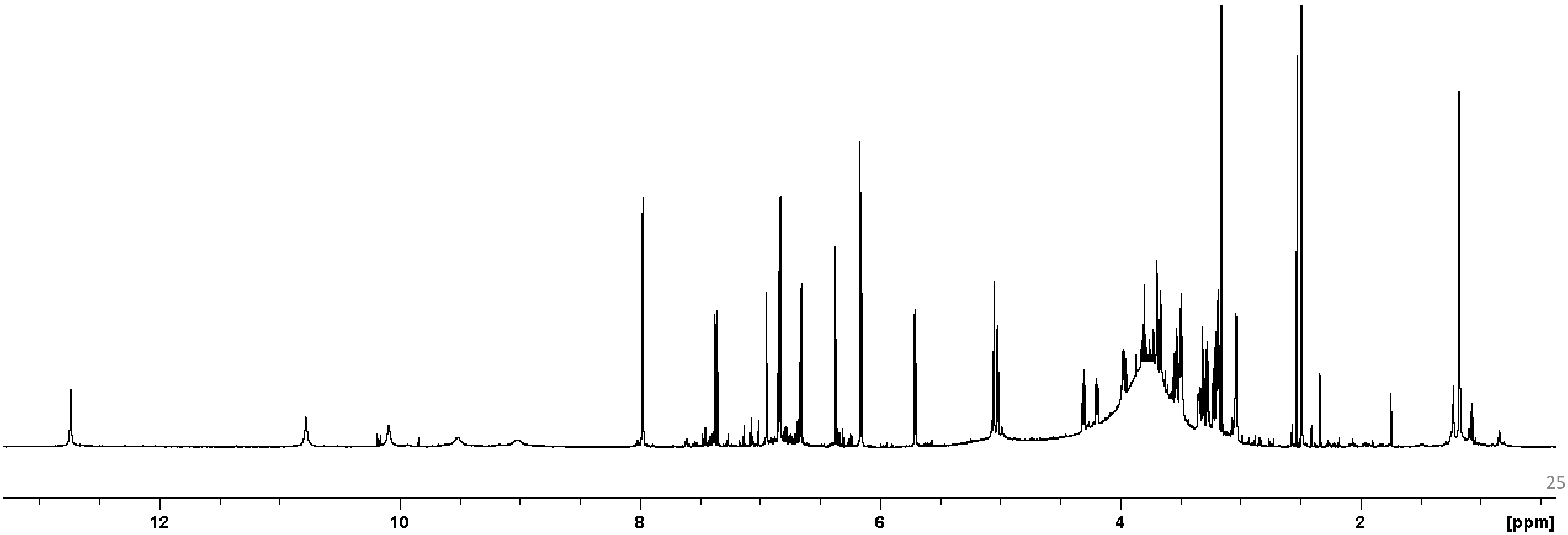

**Figure S24. 2D $^{1}H$-$^{13}C$ HMBC NMR spectrum of kaempferol 3-*O*-(2''-*O*-*β*-glucopyranosyl-(2'''-*O*-*α*-rhamnopyranosyl-6'''-*O*-(*E*)-caffeoyl))-*β*-glucopyranoside (6)**

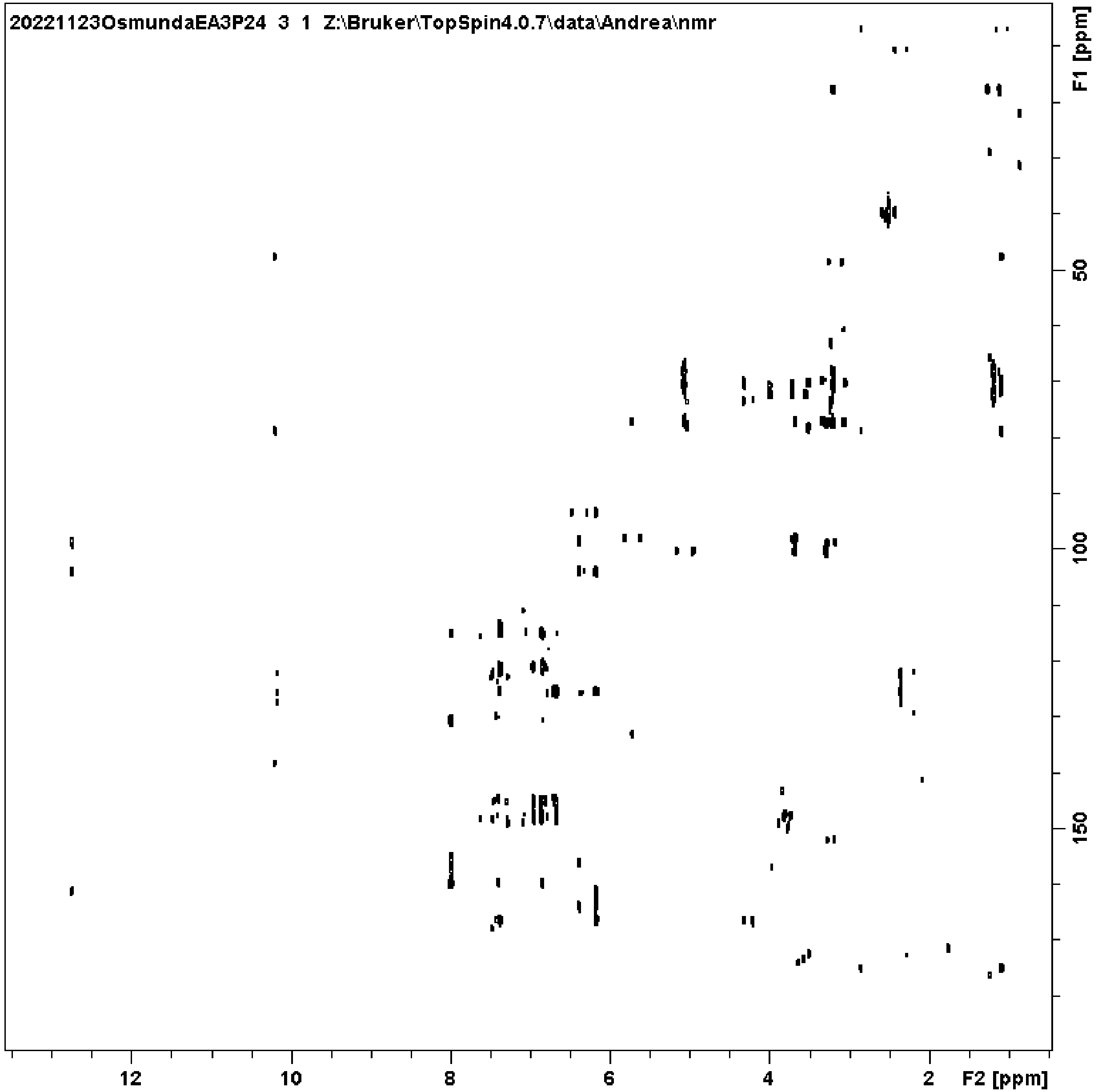

**Figure S25. 2D $^{1}H$-$^{13}C$ HSQC NMR spectrum of kaempferol 3-*O*-(2''-*O*-*β*-glucopyranosyl-(2'''-*O*-*α*-rhamnopyranosyl-6'''-*O*-(*E*)-caffeoyl))-*β*-glucopyranoside (6)**

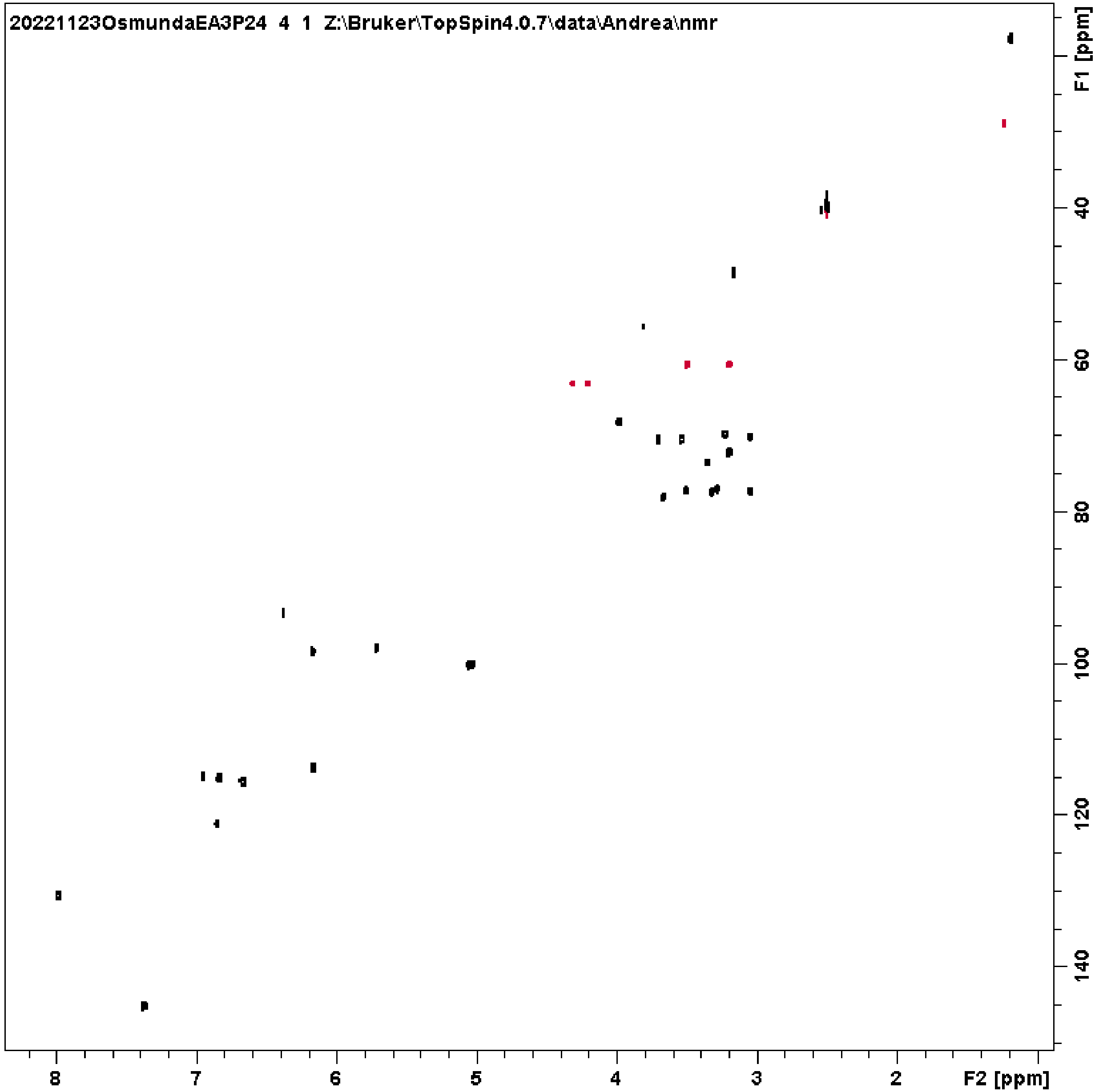

**Figure S26. 2D $^{1}$H-$^{13}$C HSQC-TOCSY NMR spectrum of kaempferol 3-*O*-(2''-*O*-*β*-glucopyranosyl-(2'''-*O*-*α*-rhamnopyranosyl-6'''-*O*-(*E*)-caffeoyl))-*β*-glucopyranoside (6)**

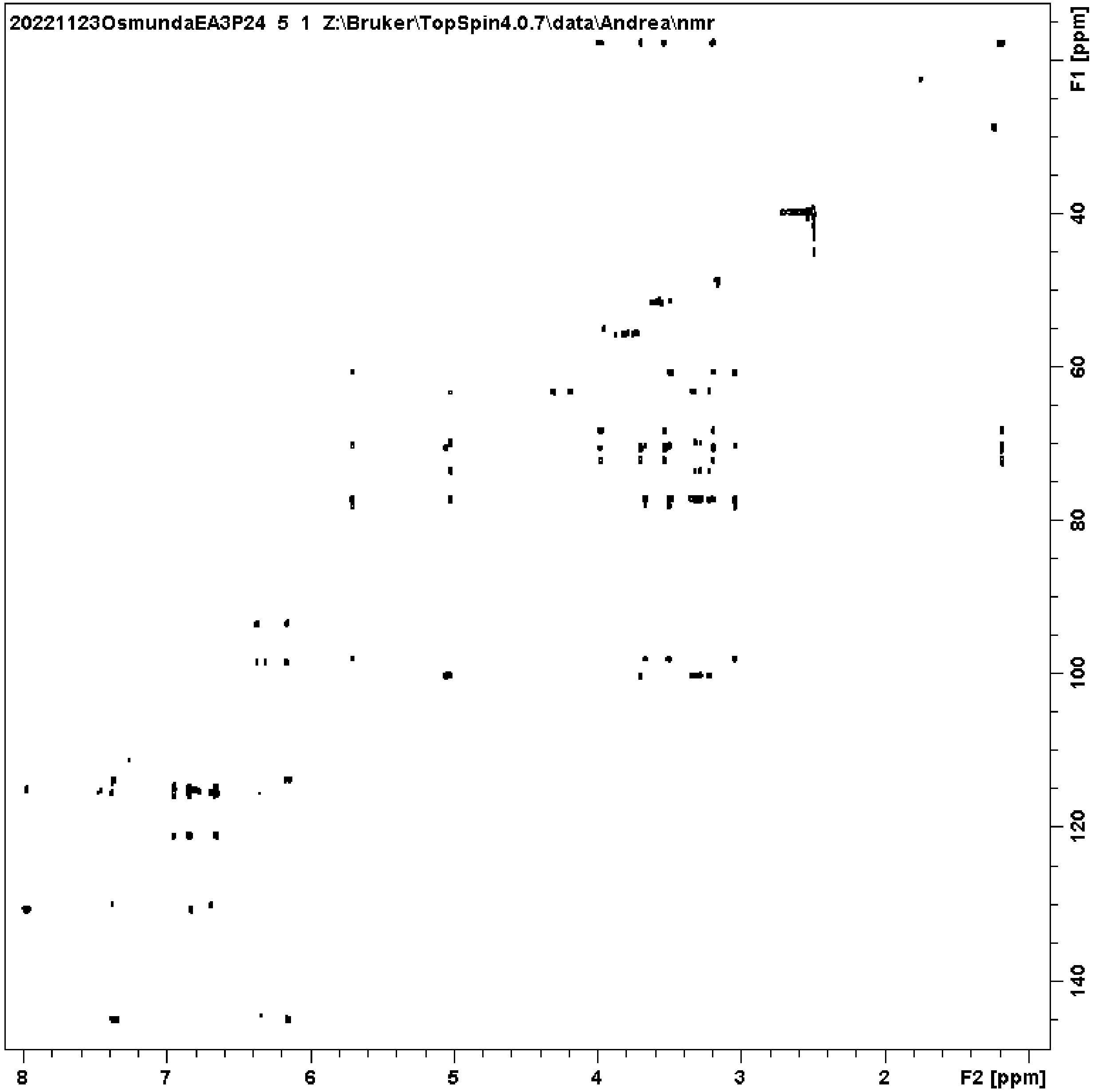

**Figure S27. 2D $^{1}H$-$^{13}C$ H2BC NMR spectrum of kaempferol 3-*O*-(2”-*O*-*β*-glucopyranosyl-(2’”-*O*-*α*-rhamnopyranosyl-6’”-*O*-(*E*)-caffeoyl))-*β*-glucopyranoside (6)**

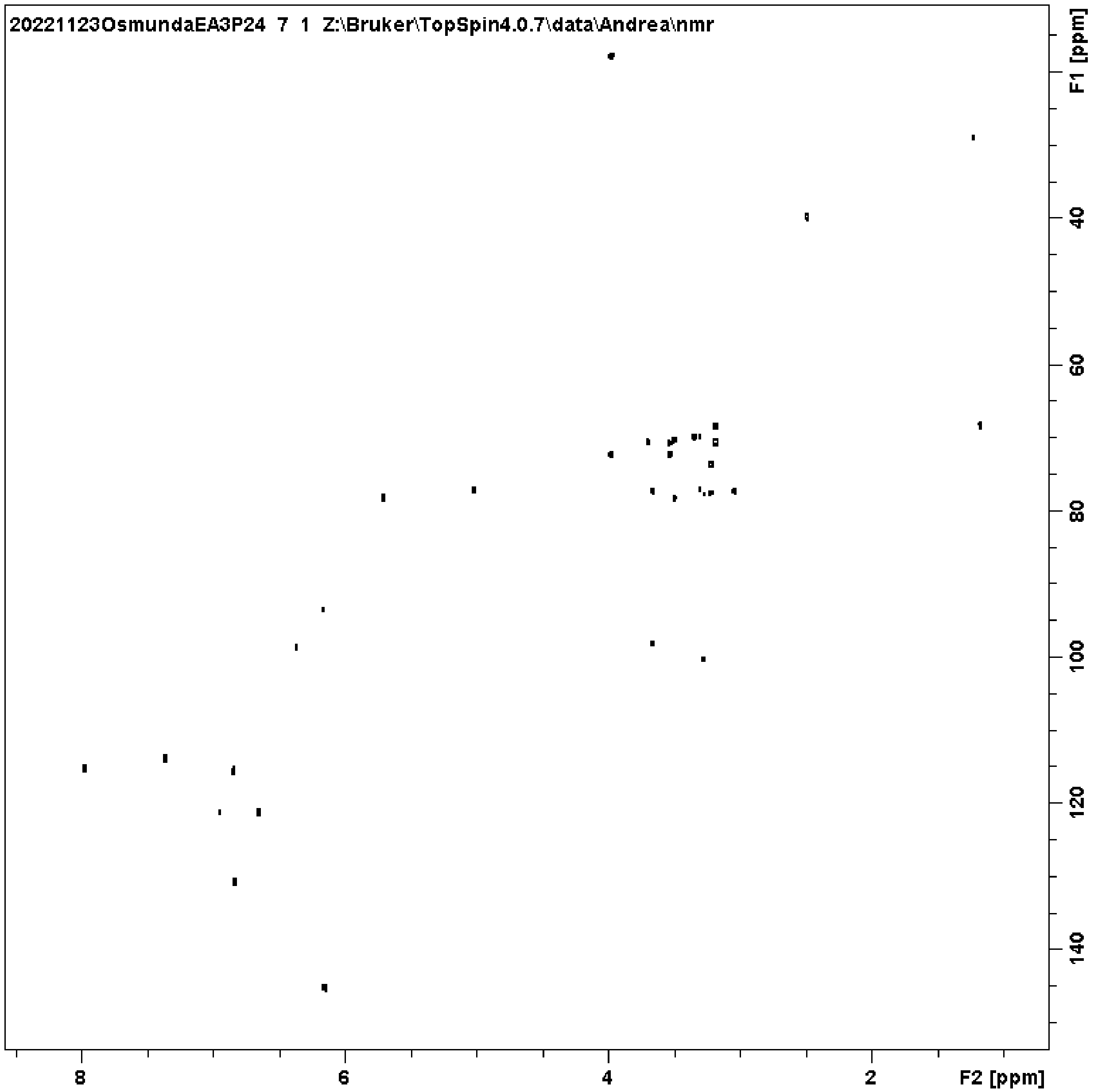

**Figure S28. 2D $^{1}H$-$^{1}H$ COSY NMR spectrum of kaempferol 3-*O*-(2’’-*O*-*β*-glucopyranosyl-(2’’’-*O*-*α*-rhamnopyranosyl-6’’’-*O*-(*E*)-caffeoyl))-*β*-glucopyranoside (6)**

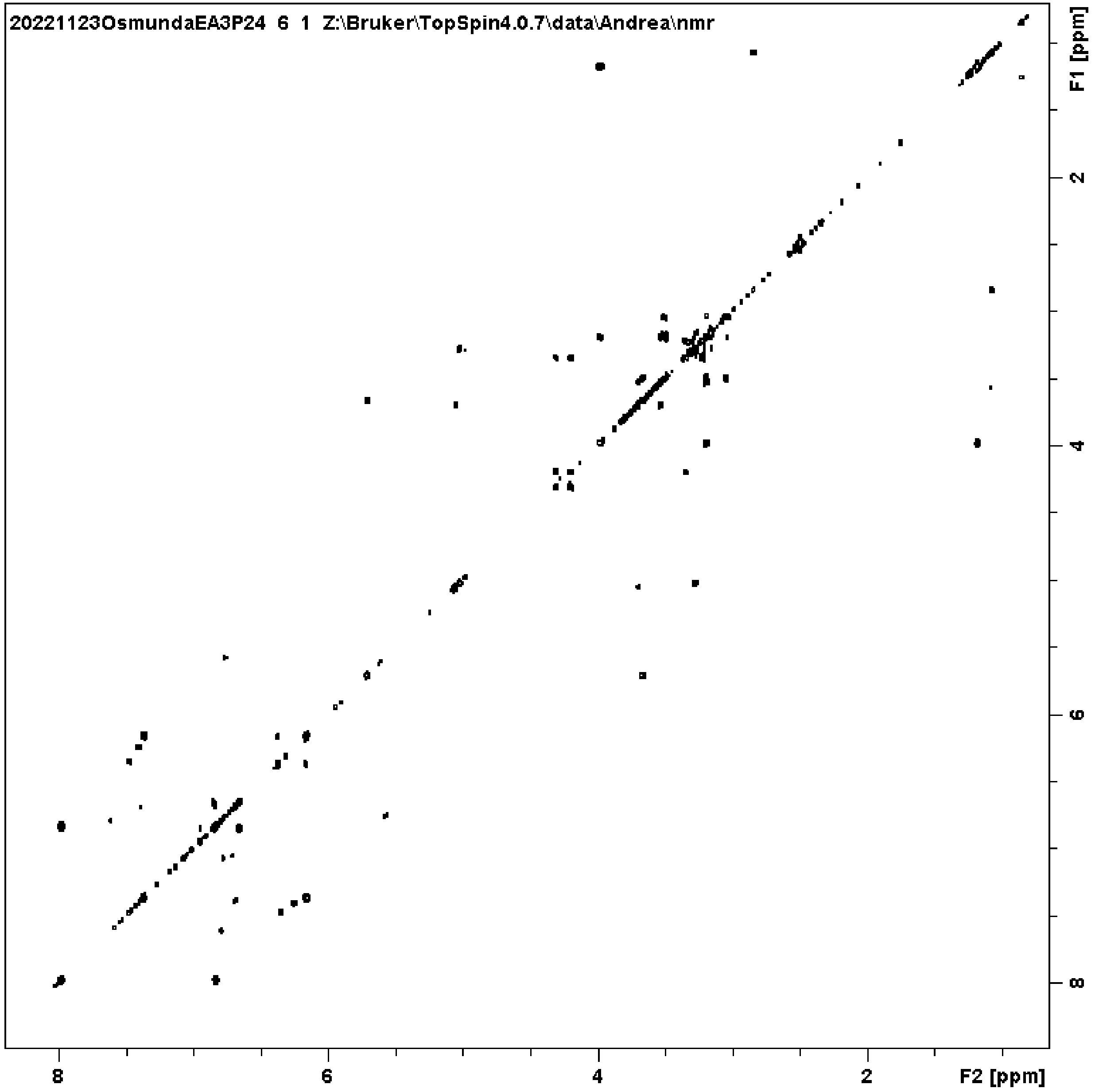

# Figure S29. 1D $^{1}$H NMR spectrum of 3-methoxy-5-hydroxy-4-olide (12)

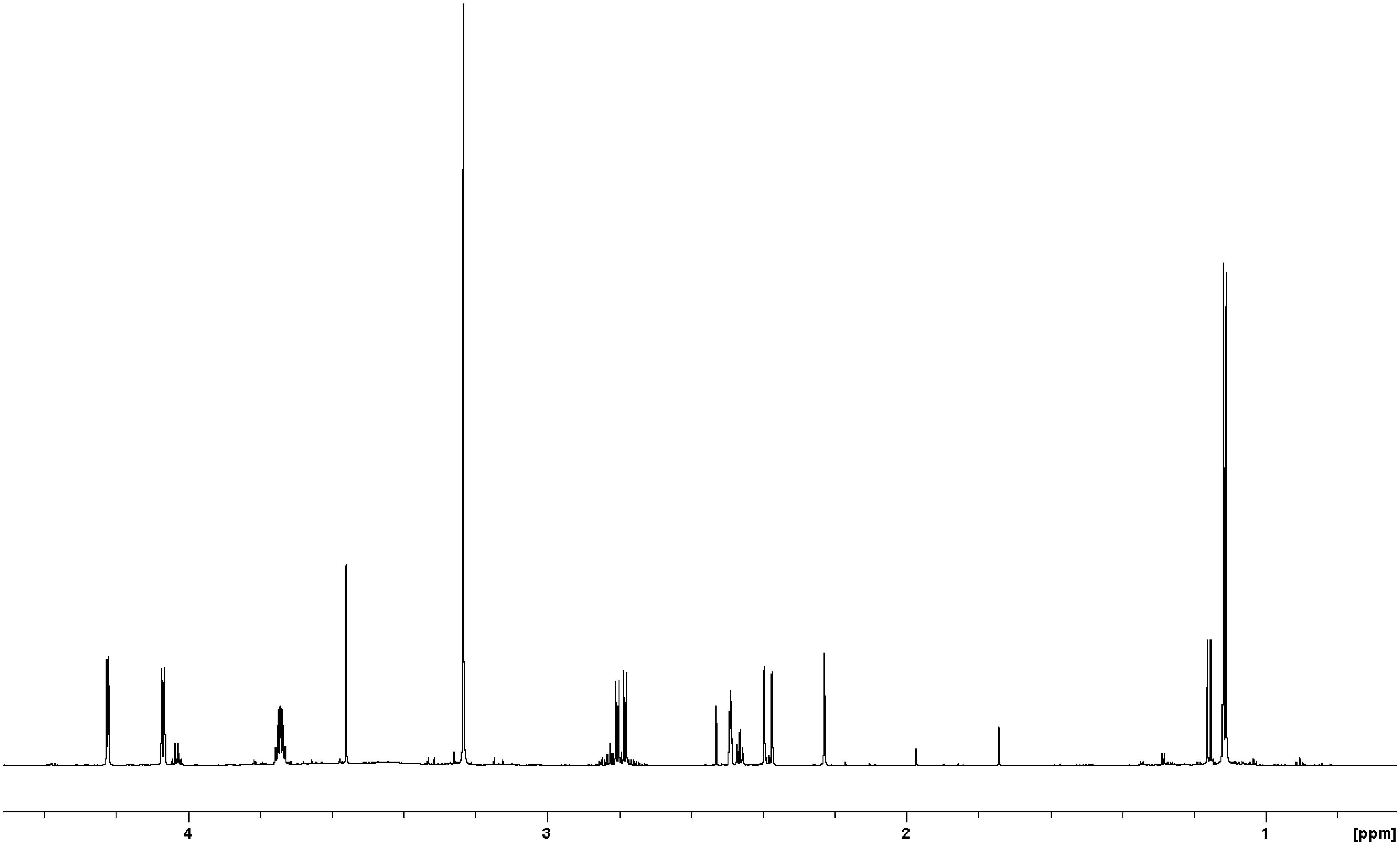

**Figure S30. 1D $^{13}$C CAPT NMR spectrum of 3-methoxy-5-hydroxy-4-olide (12)**

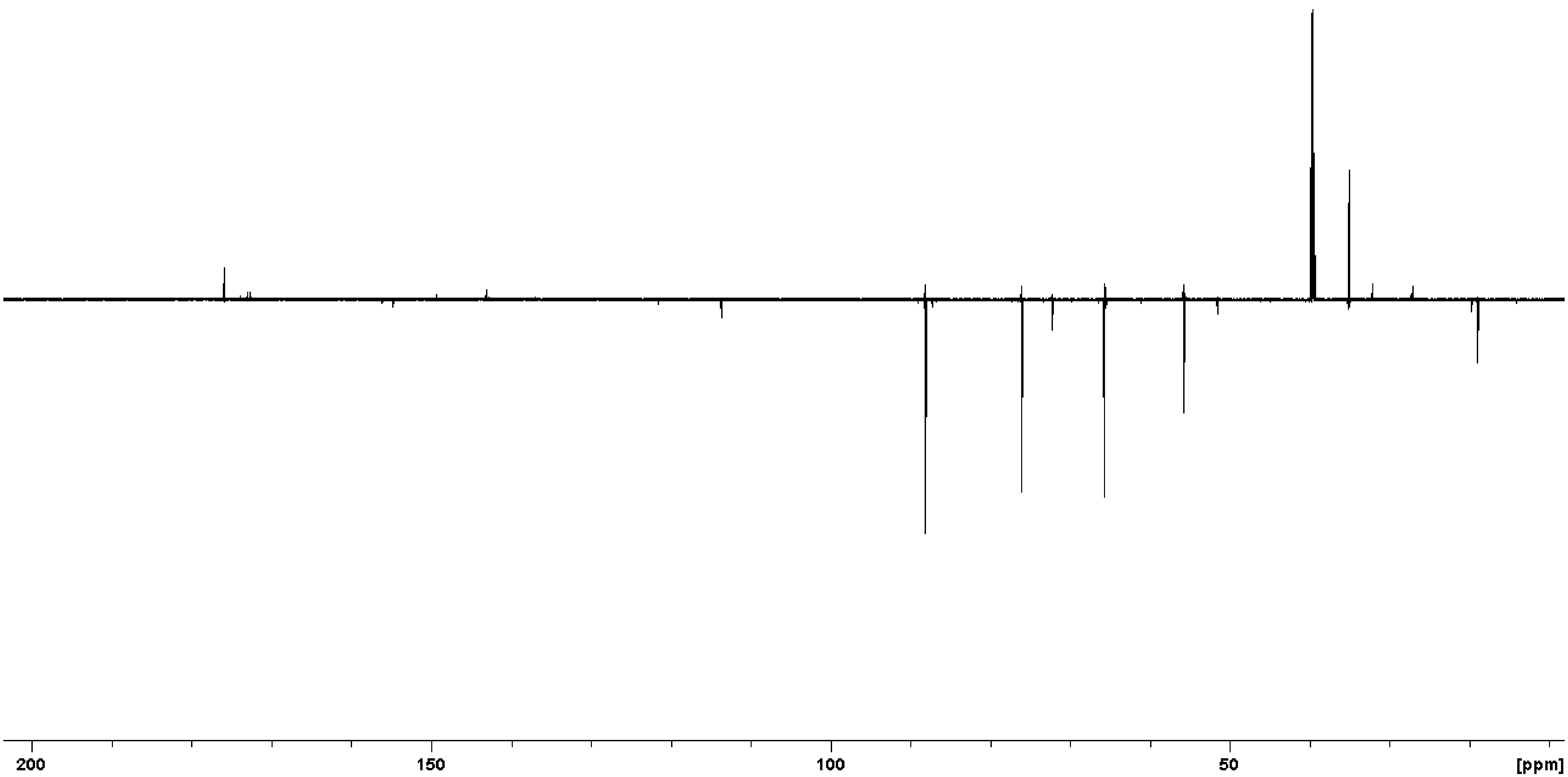

**Figure S31. 2D $^1H$-$^{13}C$ HMBC NMR spectrum of 3-methoxy-5-hydroxy-4-olide (12)**

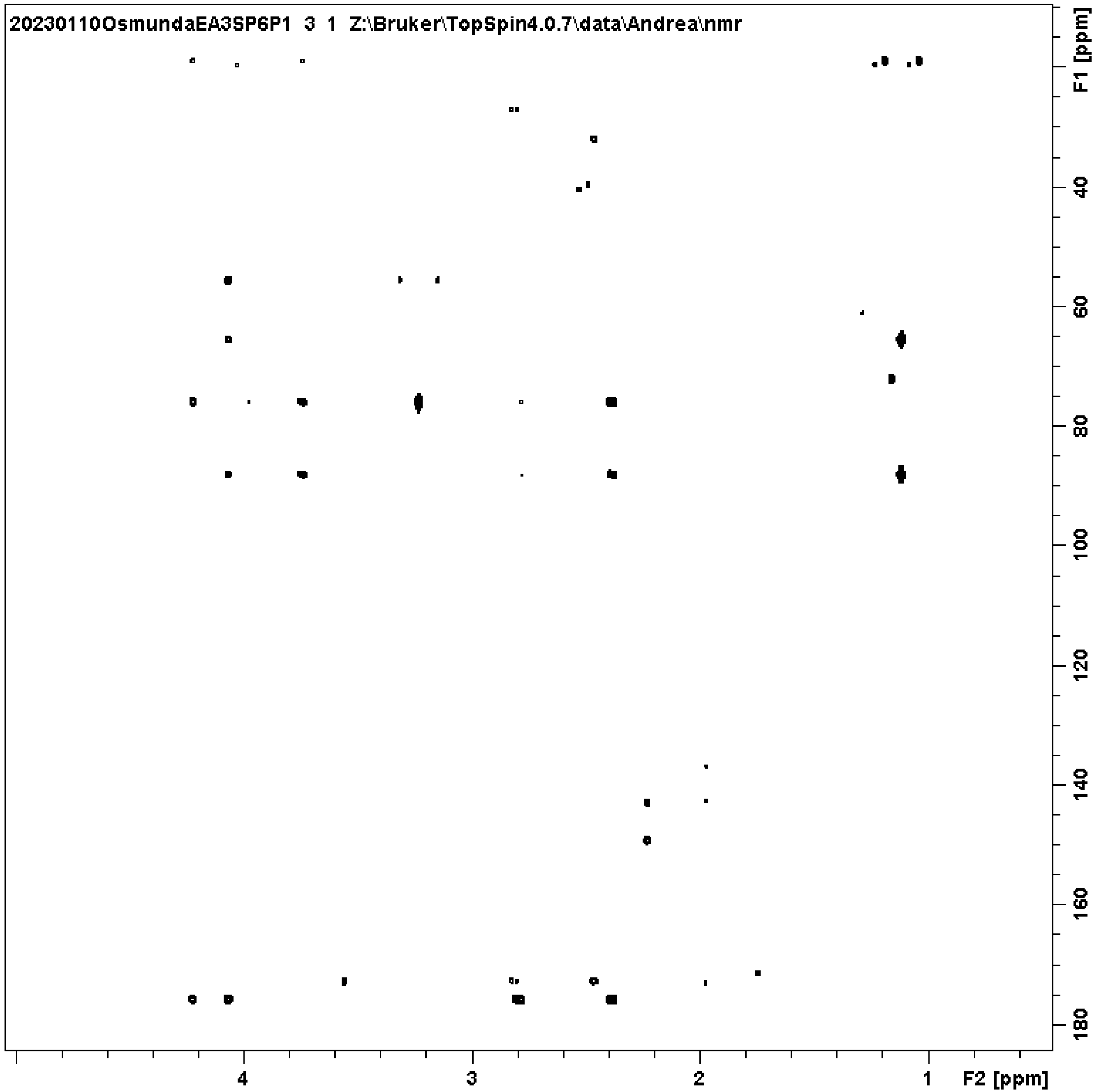

**Figure S32. 2D $^{1}H$-$^{13}C$ HSQC NMR spectrum of 3-methoxy-5-hydroxy-4-olide (12)**

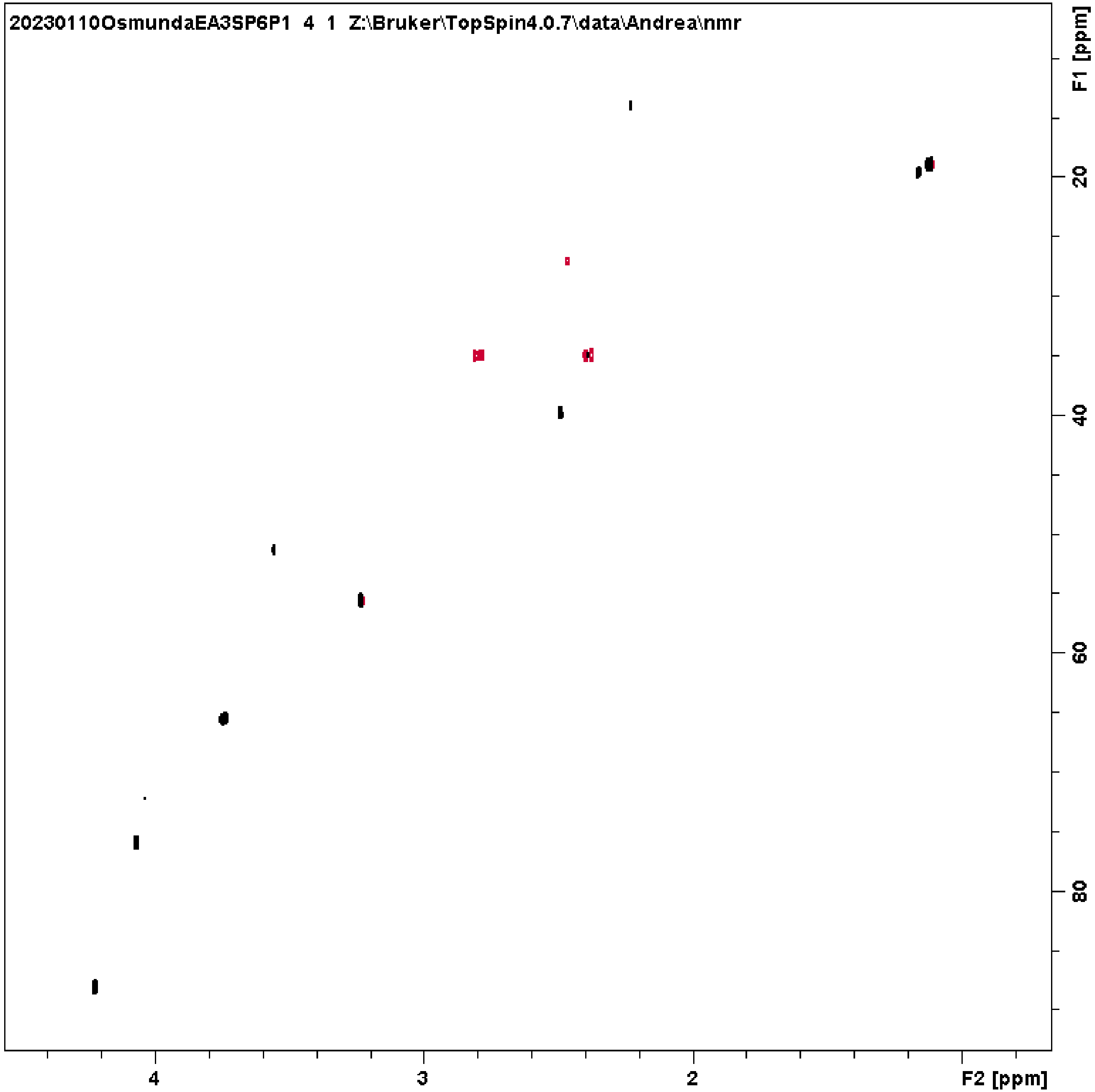

**Figure S33. 2D $^{1}H$-$^{13}C$ HSQC-TOCSY NMR spectrum of 3-methoxy-5-hydroxy-4-olide (12)**

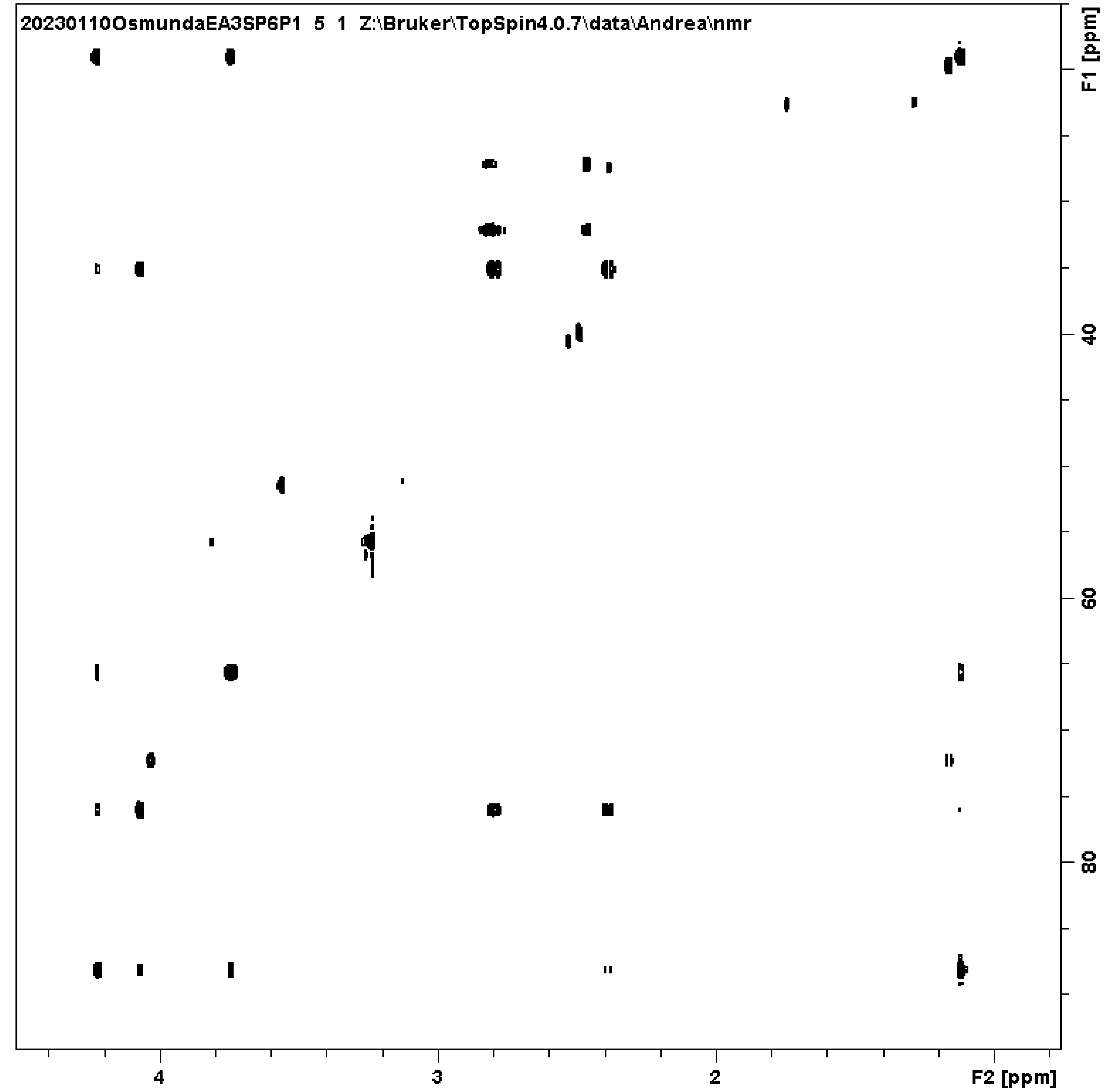

**Figure S34. 2D $^{1}H$-$^{13}C$ H2BC NMR spectrum of 3-methoxy-5-hydroxy-4-olide (12)**

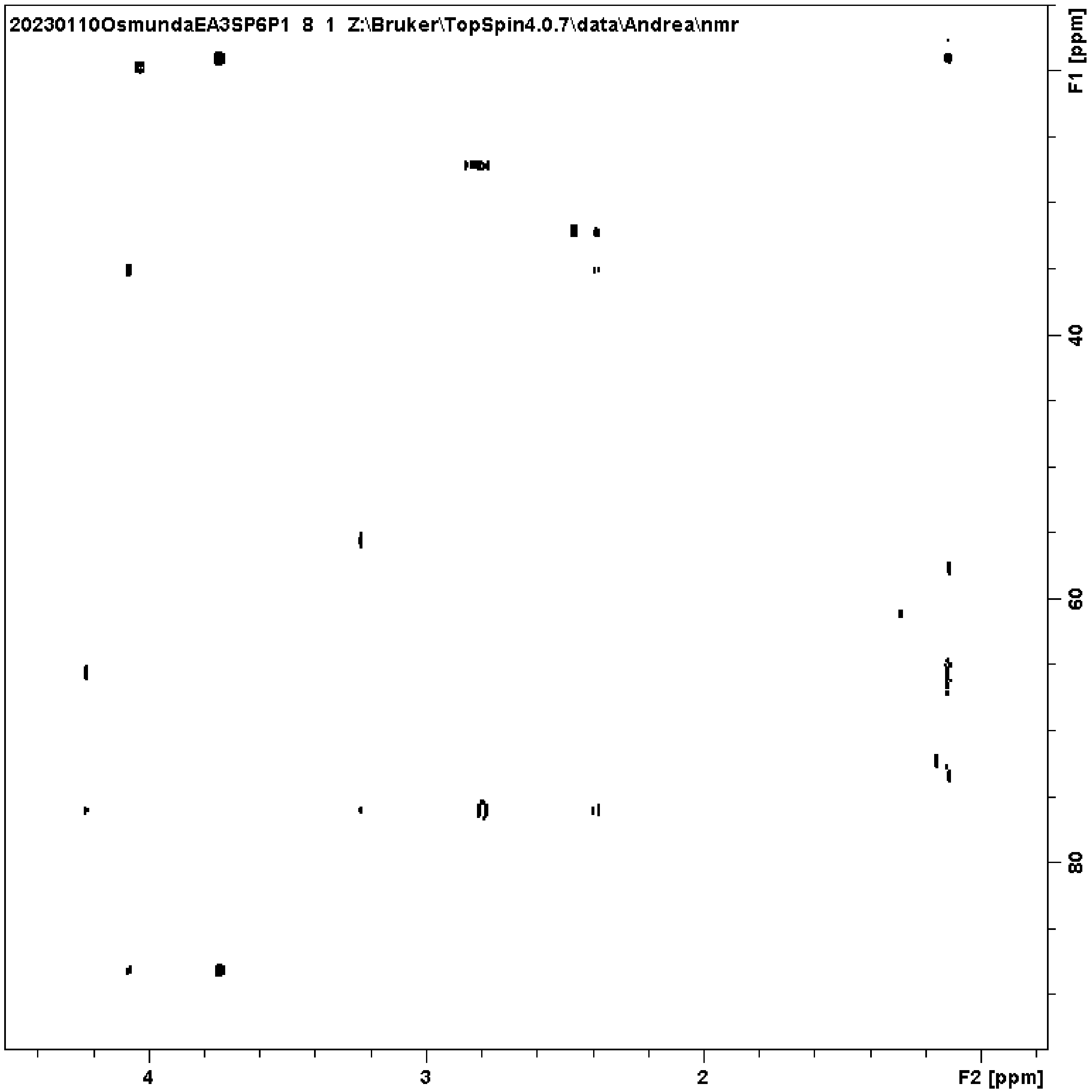

**Figure S35. 2D $^{1}H$-$^{1}H$ COSY NMR spectrum of 3-methoxy-5-hydroxy-4-olide**

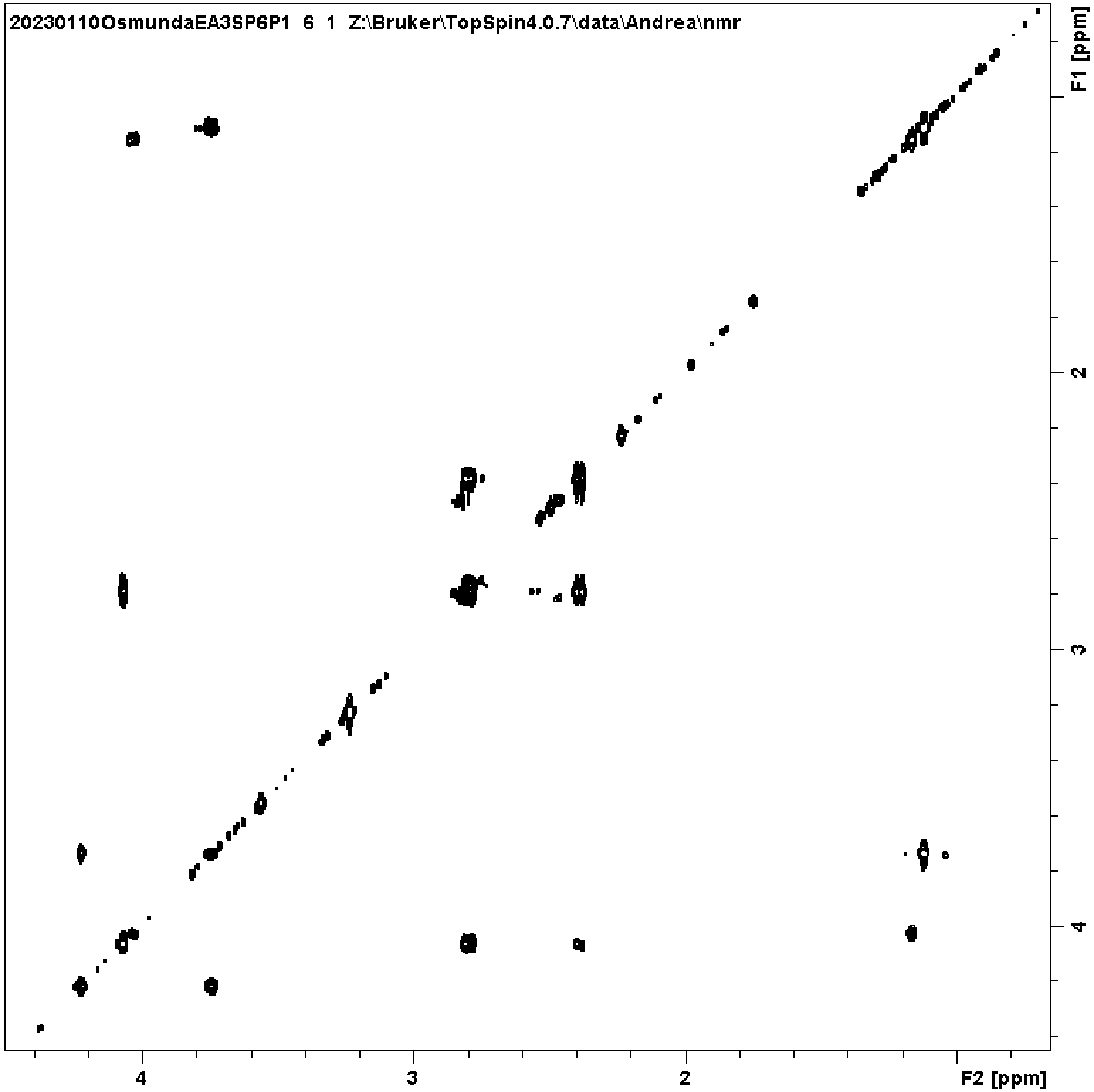

# Figure S36. 2D $^1H$-$^1H$ ROESY NMR spectrum of 3-methoxy-5-hydroxy-4-olide (12)

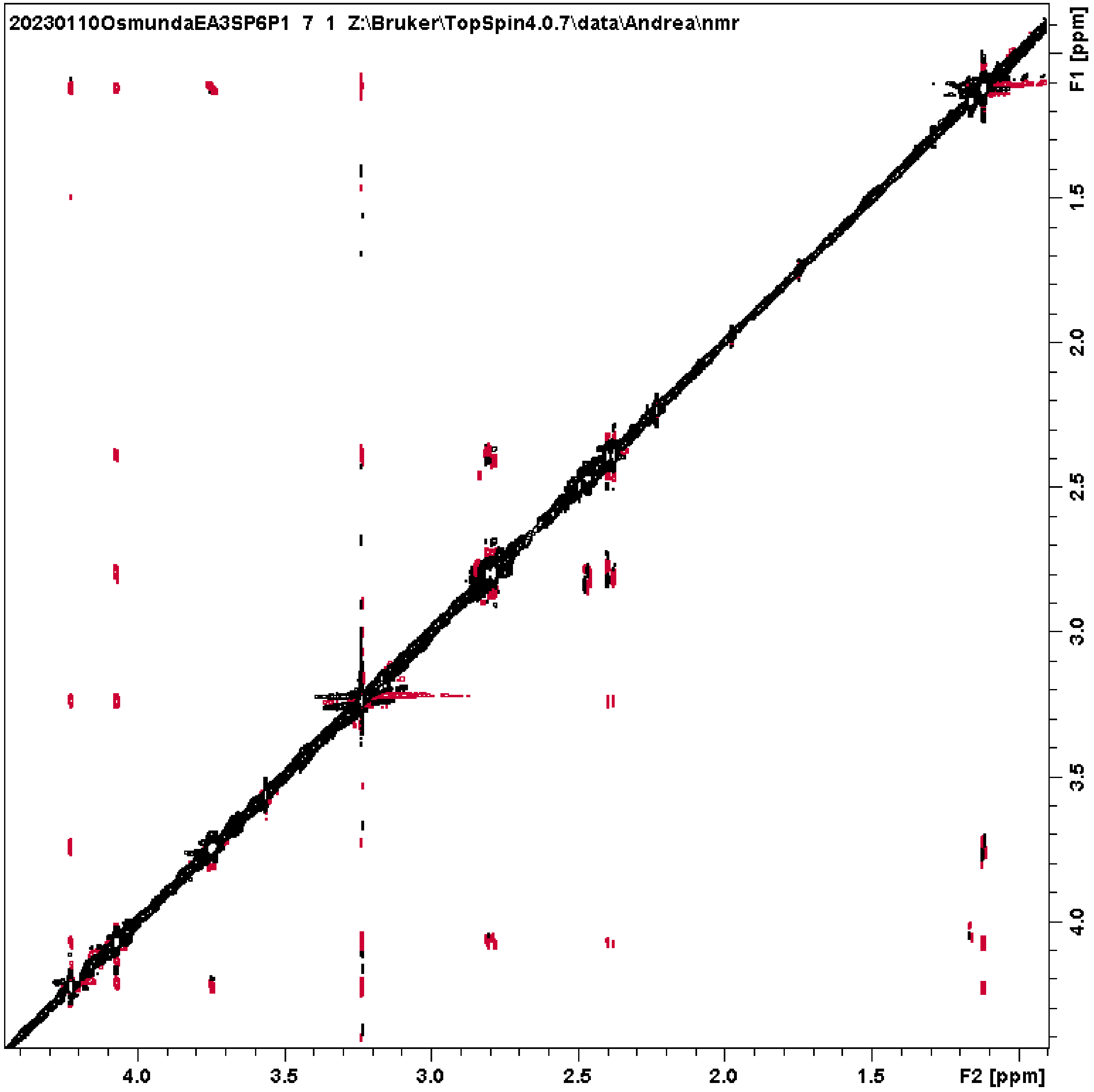

**Figure S37. 1D $^{1}H$ NMR spectrum of 4-hydroxy-3-(3'-hydroxy-4'-(hydroxymethyl)-oxotetrafuranone-5-methyl tetrahydropyranone (13)**

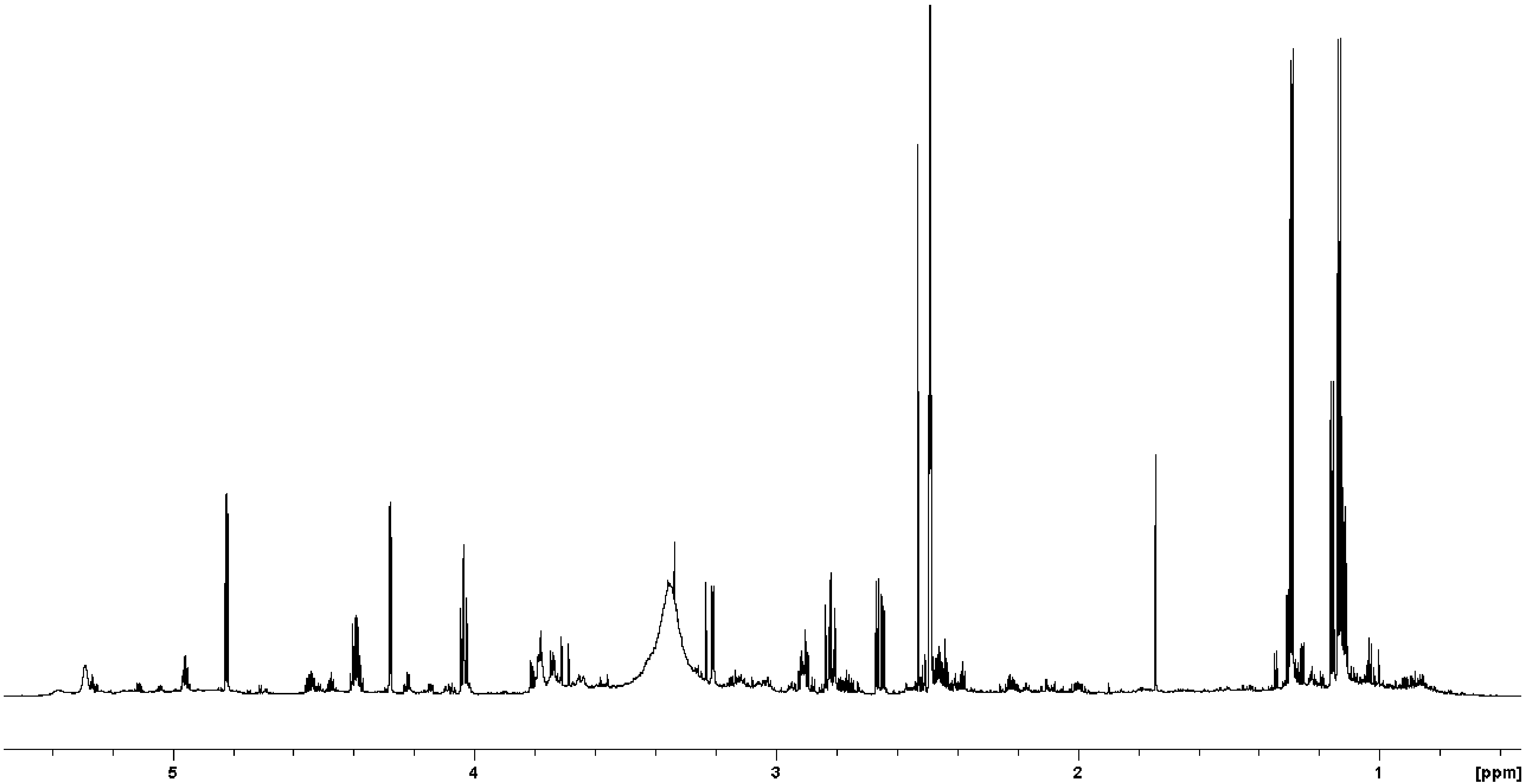

**Figure S38. 1D $^{13}$C CAPT NMR spectrum of 4-hydroxy-3-(3'-hydroxy-4'-(hydroxymethyl)-oxotetrafuranone-5-methyl tetrahydropyranone (13)**

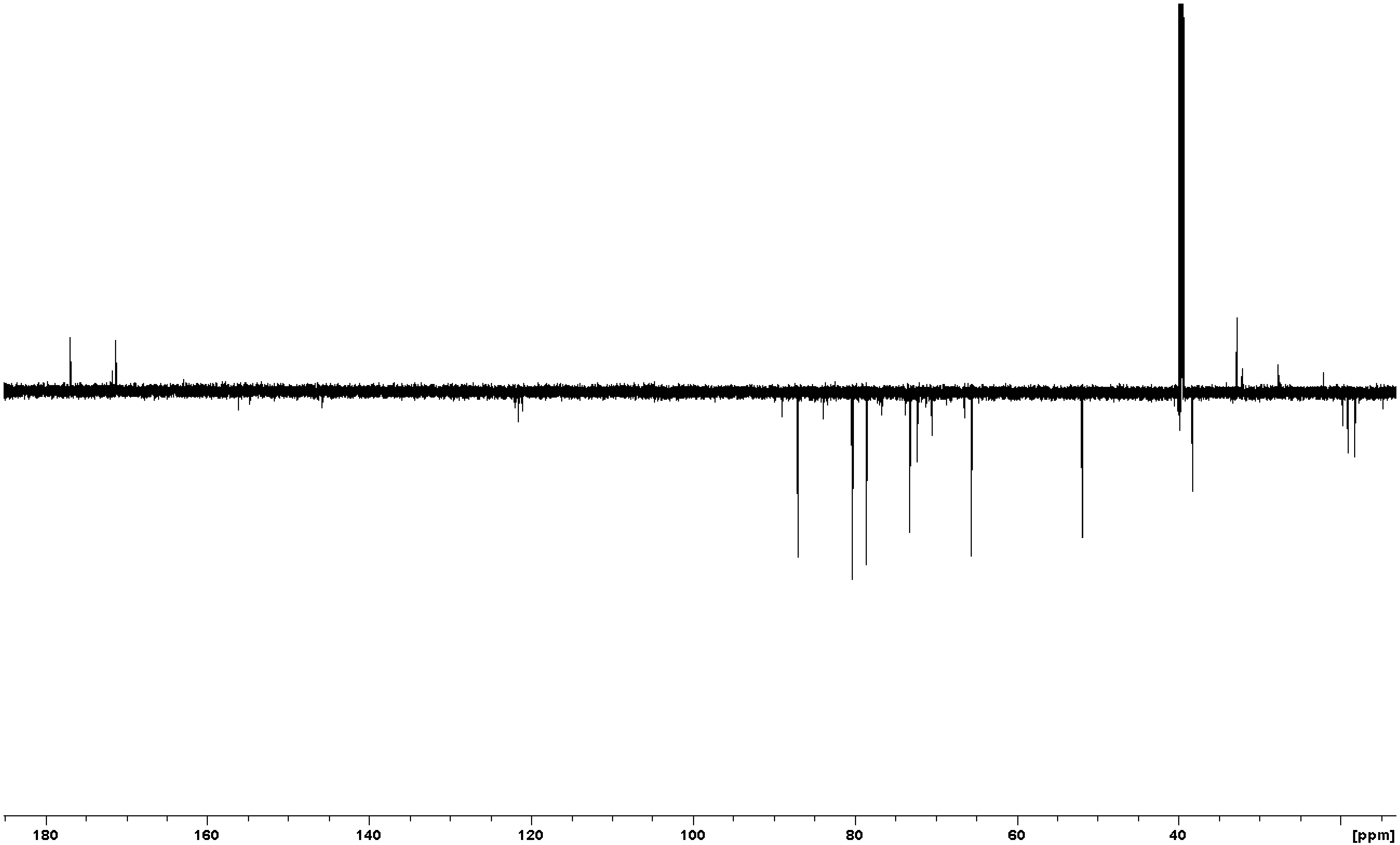

**Figure S39. 2D $^{1}H$-$^{13}C$ HMBC NMR spectrum of 4-hydroxy-3-(3'-hydroxy-4'-(hydroxymethyl)-oxotetrafuranone-5-methyl tetrahydropyranone (13)**

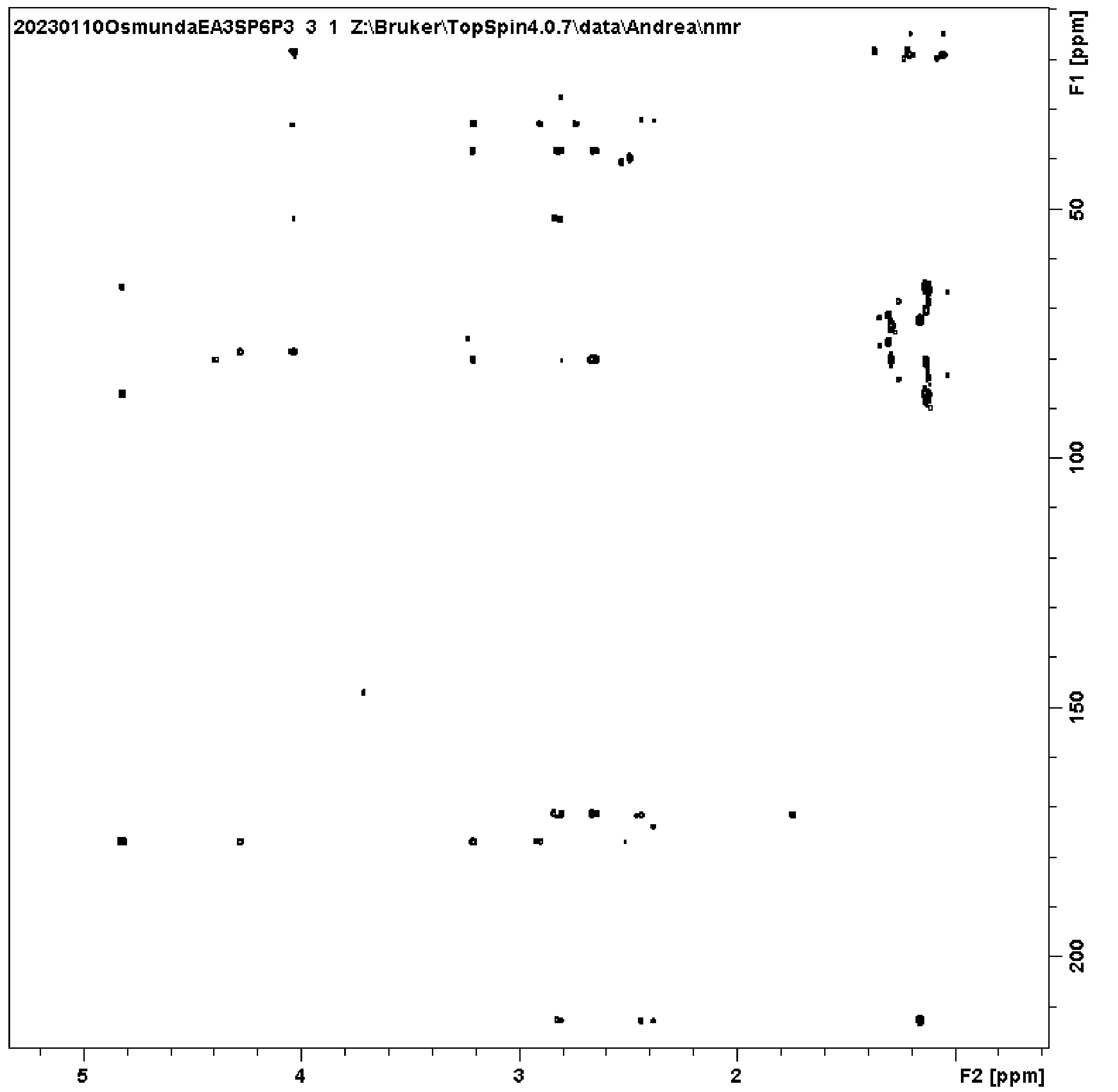

**Figure S40. 2D $^{1}H$-$^{13}C$ HSQC NMR spectrum of 4-hydroxy-3-(3'-hydroxy-4'-(hydroxymethyl)-oxotetrafuranone-5-methyl tetrahydropyranone (13)**

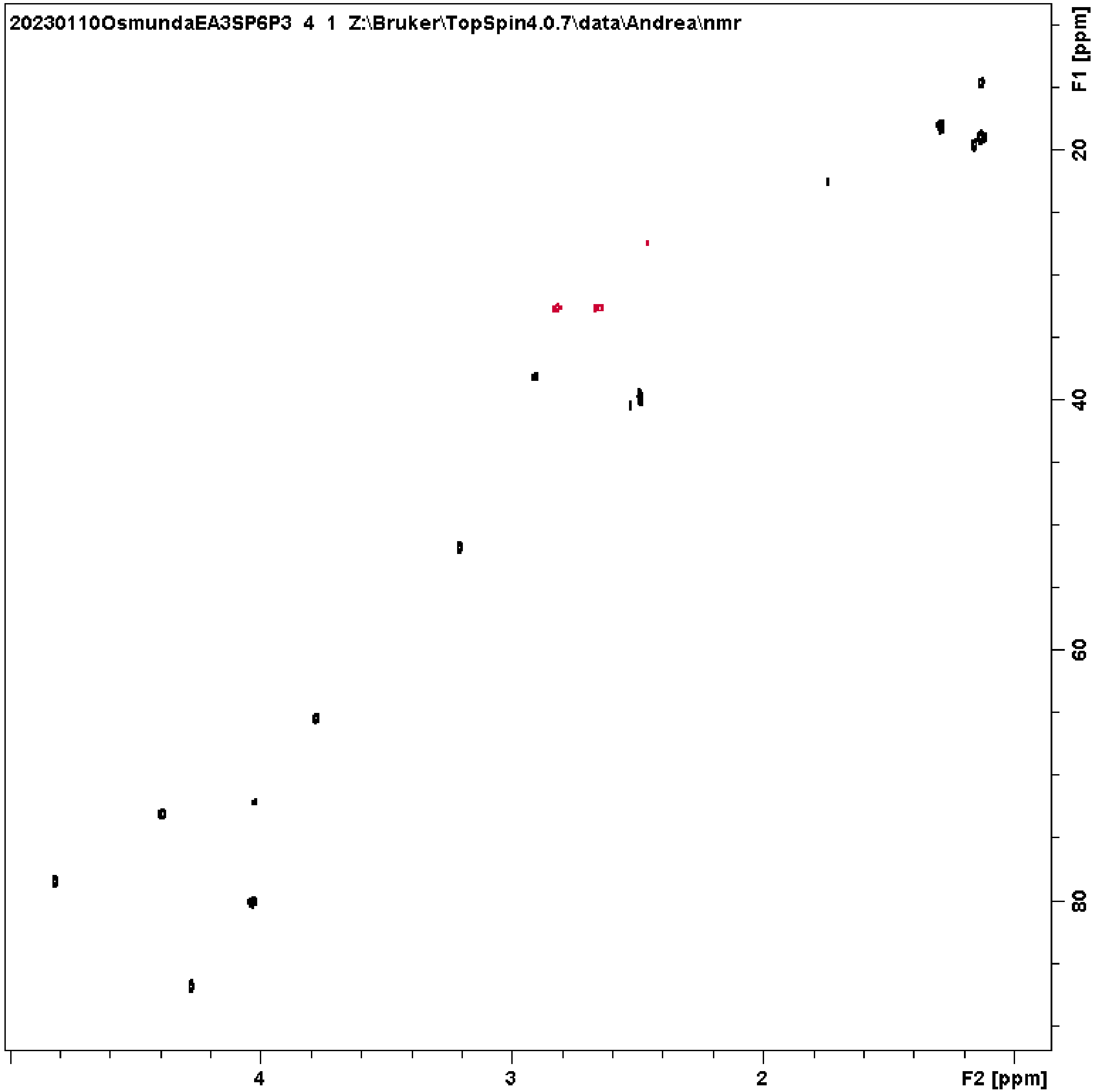

**Figure S41. 2D $^{1}H$-$^{13}C$ HSQC-TOCSY NMR spectrum of 4-hydroxy-3-(3’-hydroxy-4’-(hydroxymethyl)-oxotetrafuranone-5-methyl tetrahydropyranone (13)**

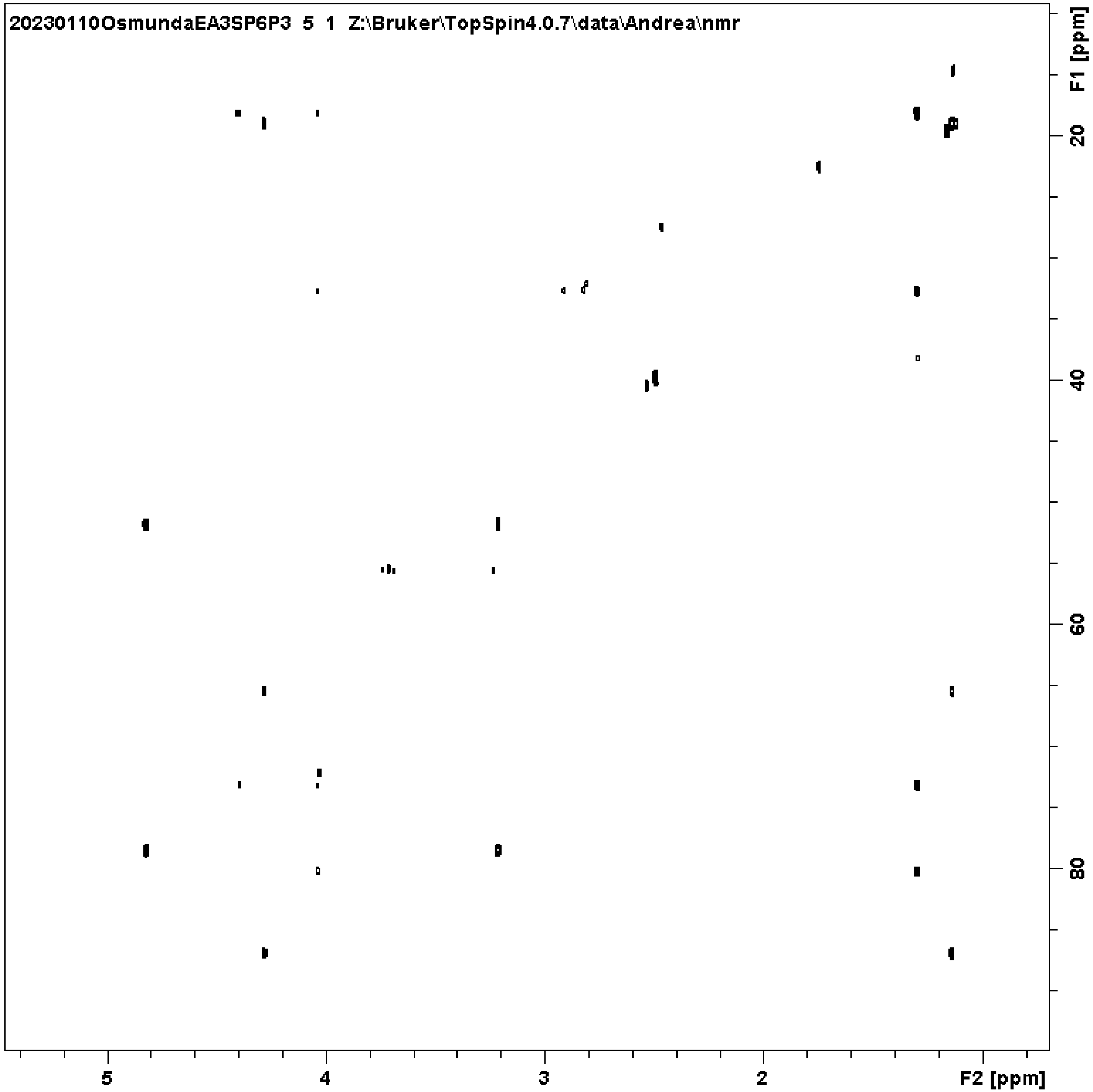

**Figure S42. 2D $^{1}H$-$^{13}C$ H2BC NMR spectrum of 4-hydroxy-3-(3'-hydroxy-4'-(hydroxymethyl)-oxotetrafuranone-5-methyl tetrahydropyranone (13)**

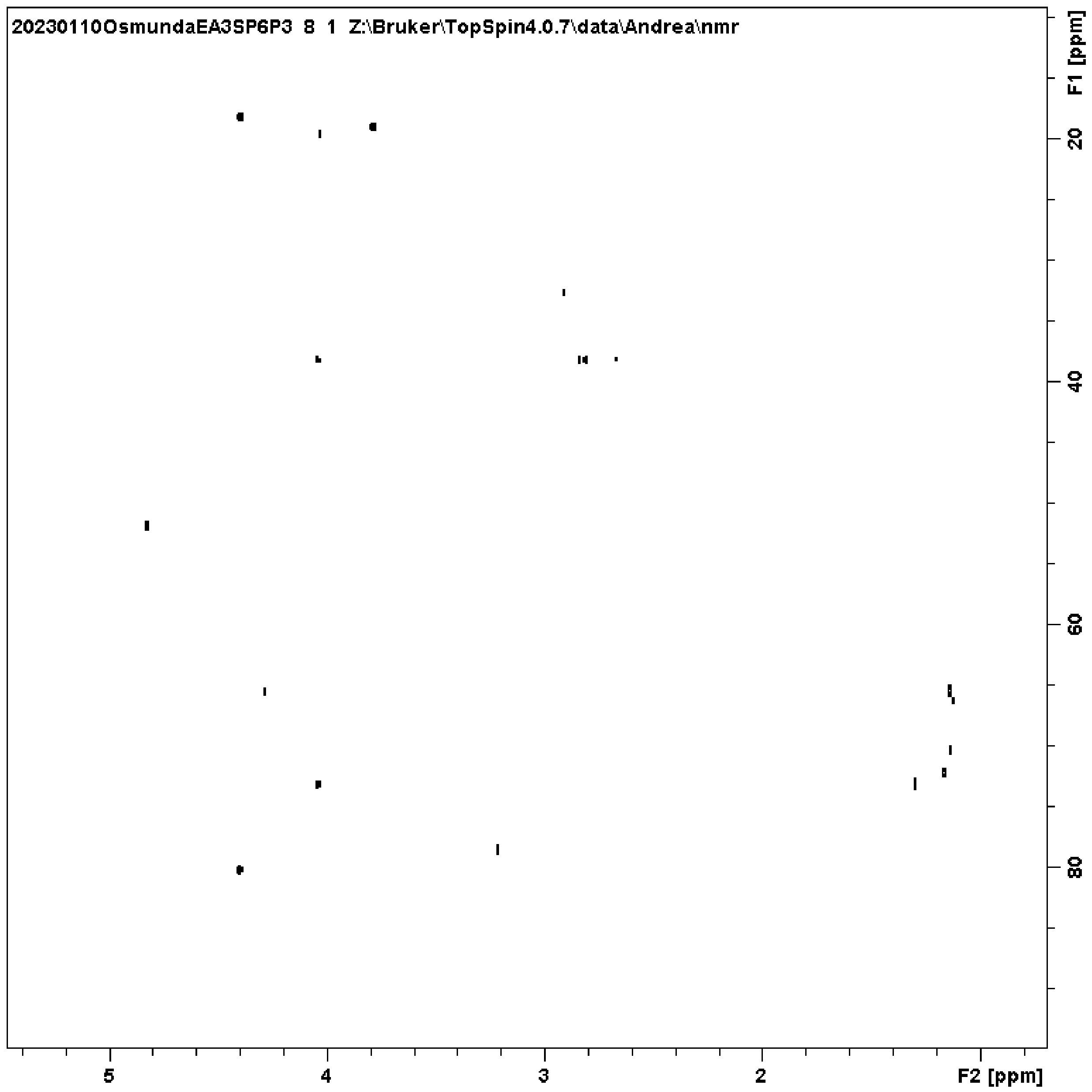

**Figure S43. 2D $^{1}H$-$^{1}H$ COSY NMR spectrum of 4-hydroxy-3-(3'-hydroxy-4'-(hydroxymethyl)-oxotetrafuranone-5-methyl tetrahydropyranone (13)**

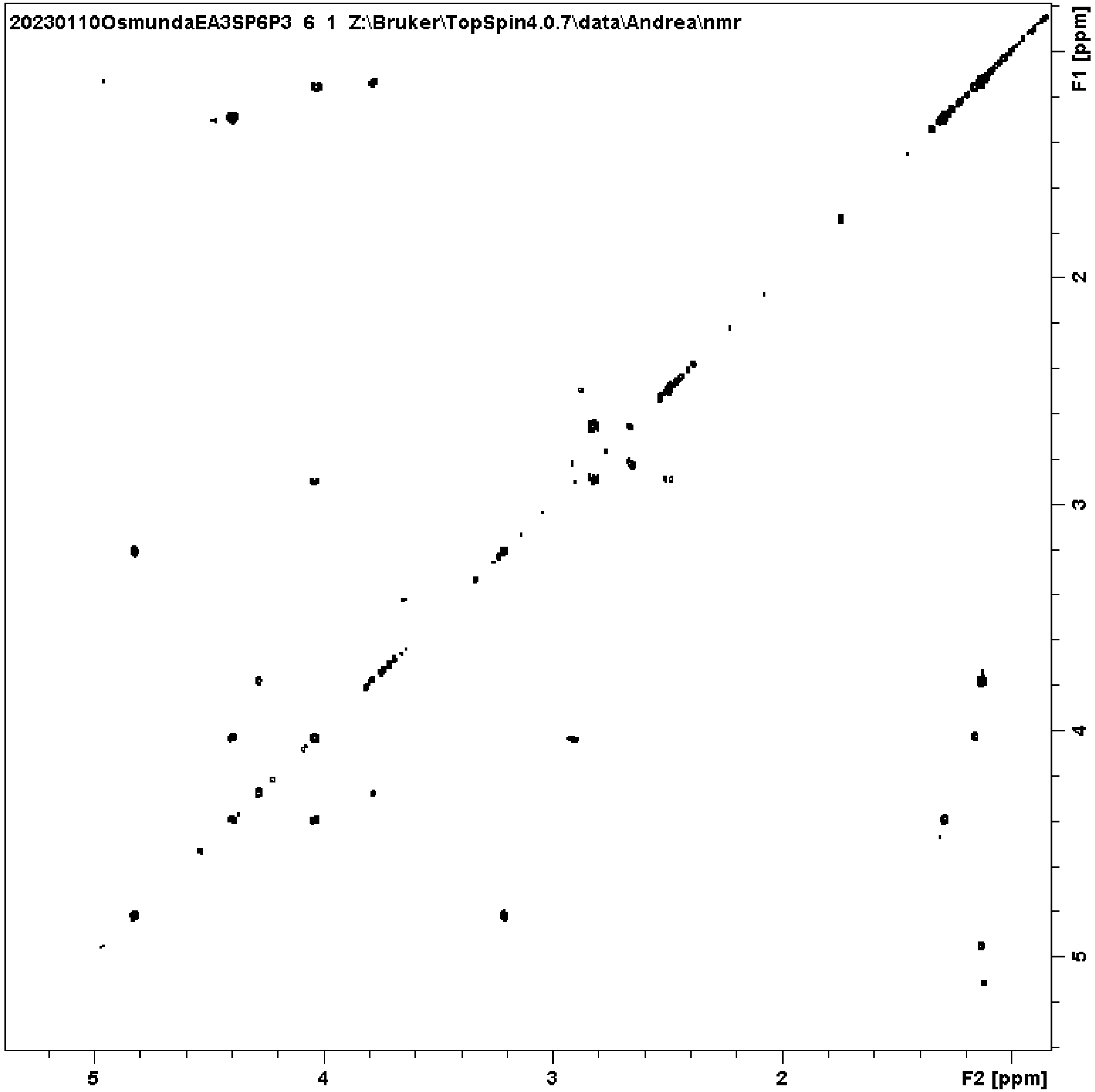

**Figure S44. 2D $^{1}H$-$^{1}H$ ROESY NMR spectrum of 4-hydroxy-3-(3'-hydroxy-4'-(hydroxymethyl)-oxotetrafuranone-5-methyl tetrahydropyranone (13)**

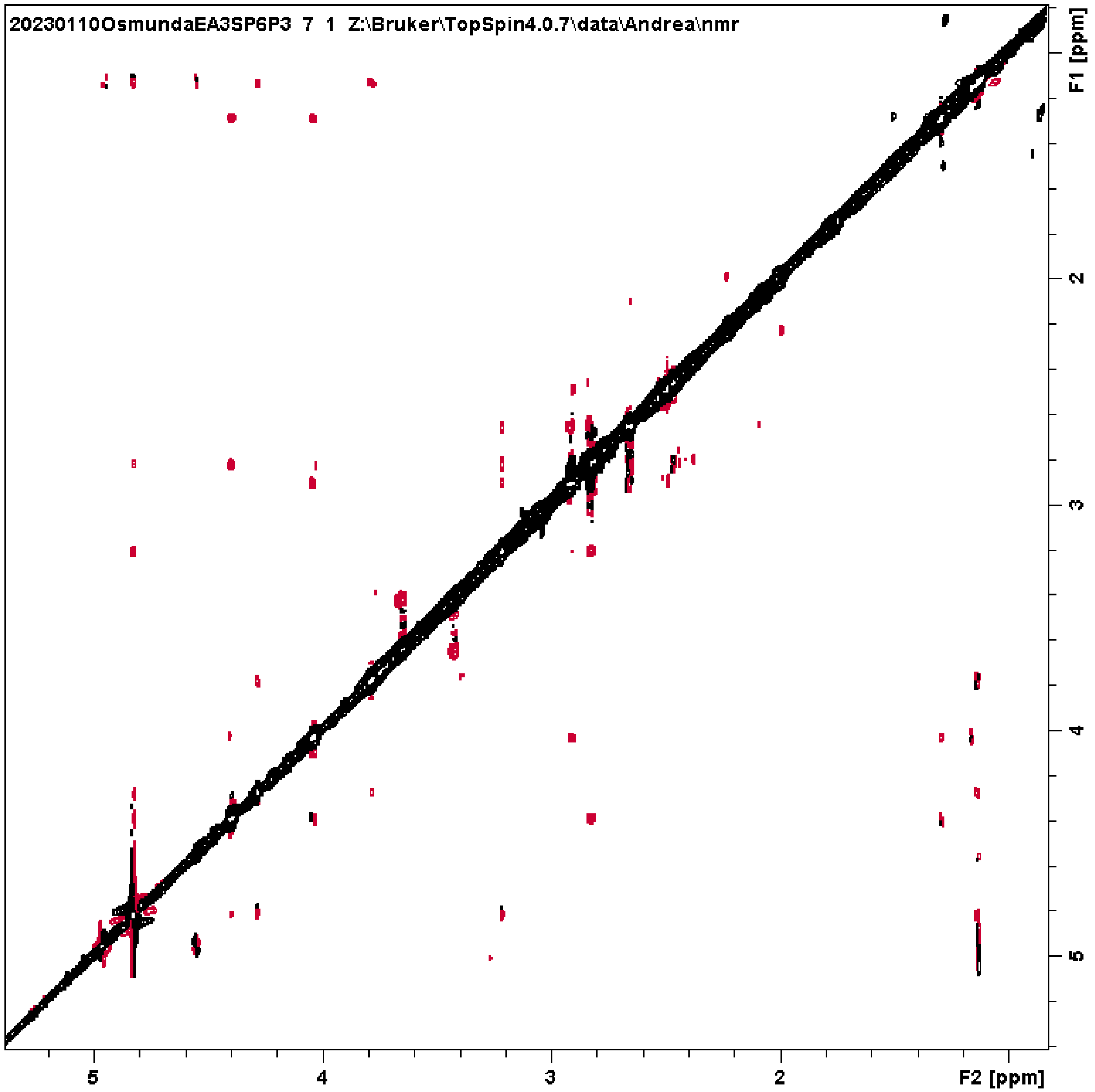

**Figure S45. 1D $^{1}$H NMR spectrum of 4-*O*-(5-hydroxy-4-oxohexanoyl)-osmundalactone (15)**

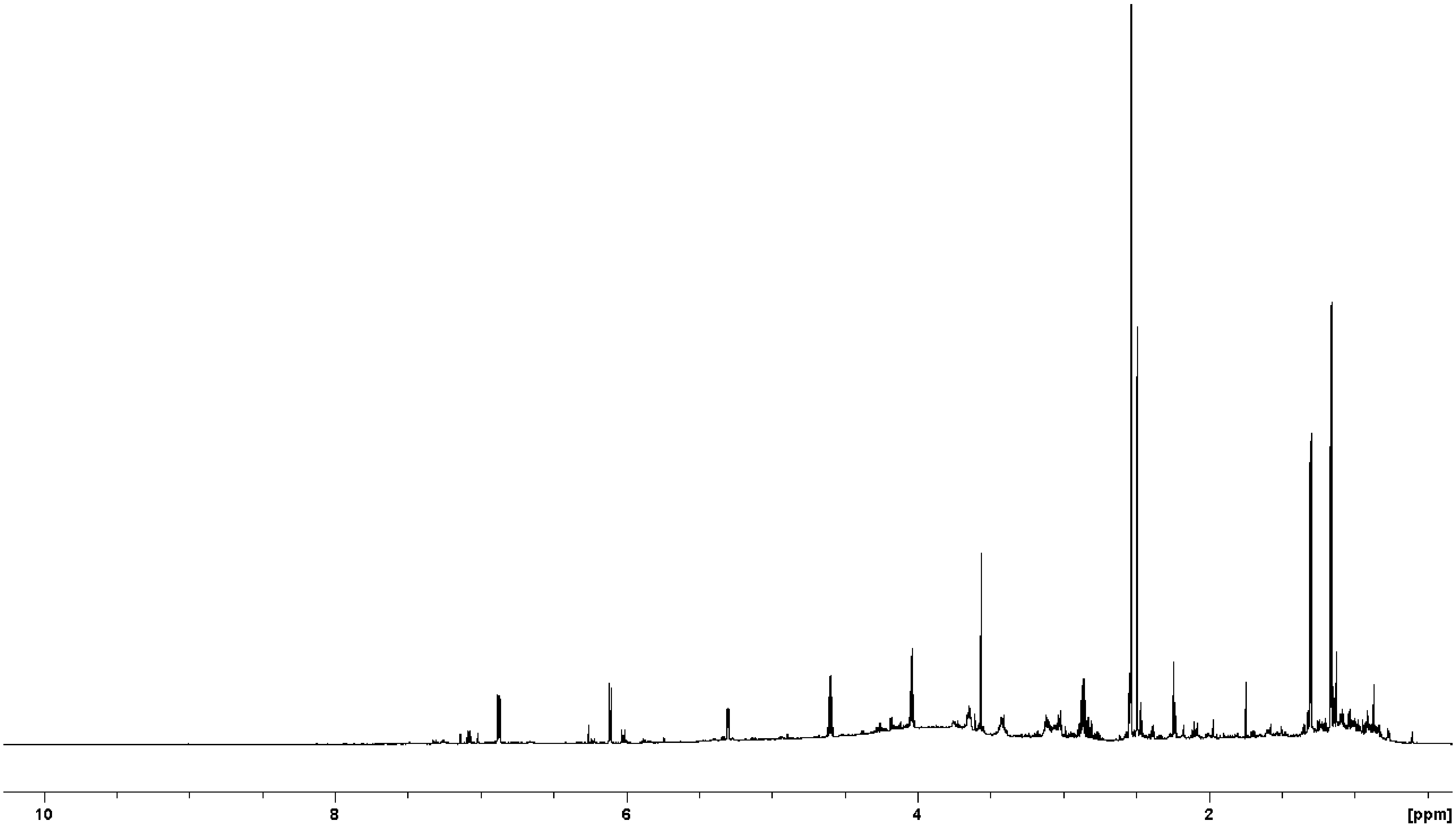

**Figure S46. 1D $^{13}$C CAPT NMR spectrum of 4-*O*-(5-hydroxy-4-oxohexanoyl)-osmundalactone (15)**

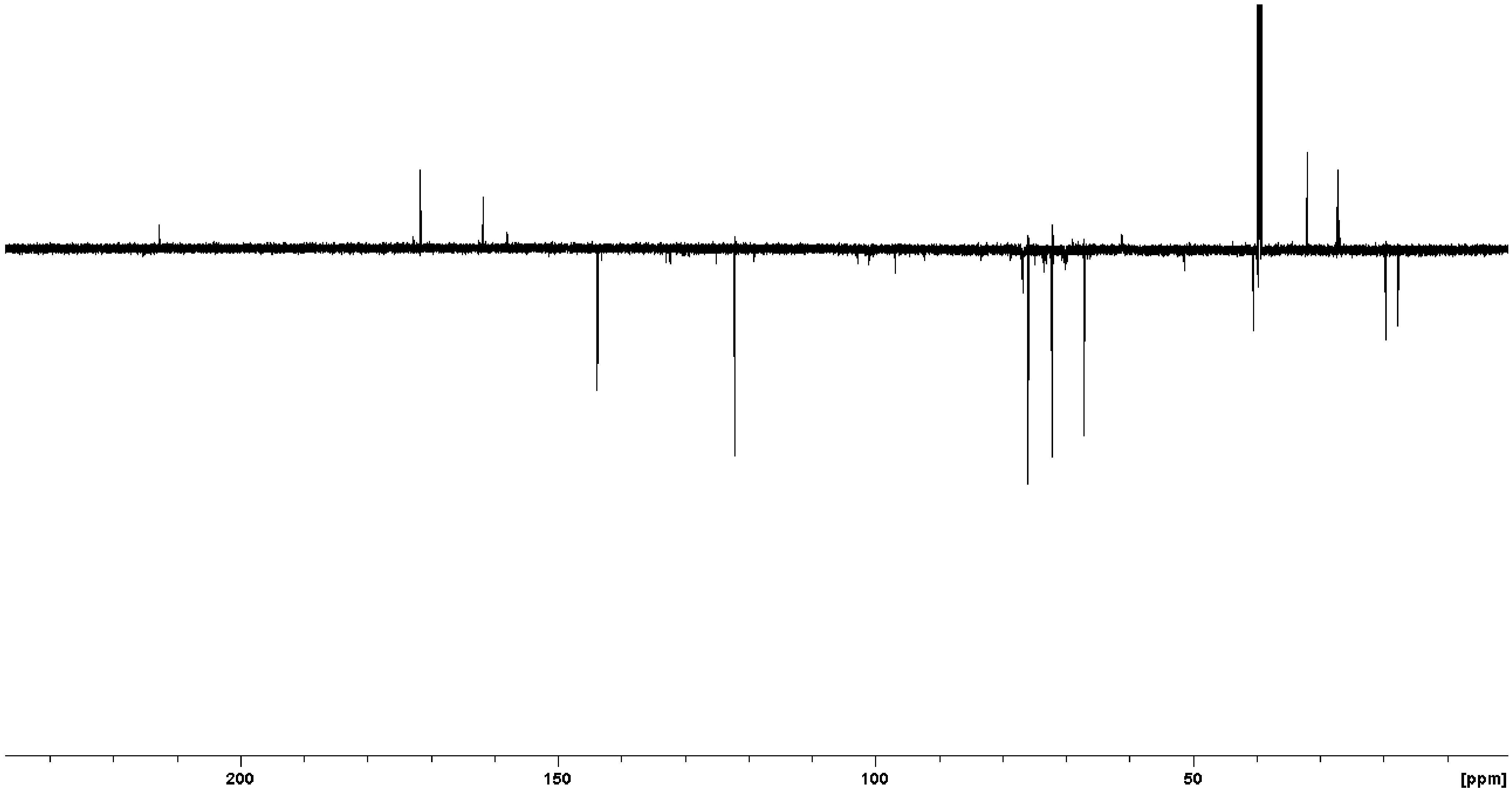

**Figure S47. 2D $^{1}H$-$^{13}C$ HMBC NMR spectrum of 4-*O*-(5-hydroxy-4-oxohexanoyl)-osmundalactone (15)**

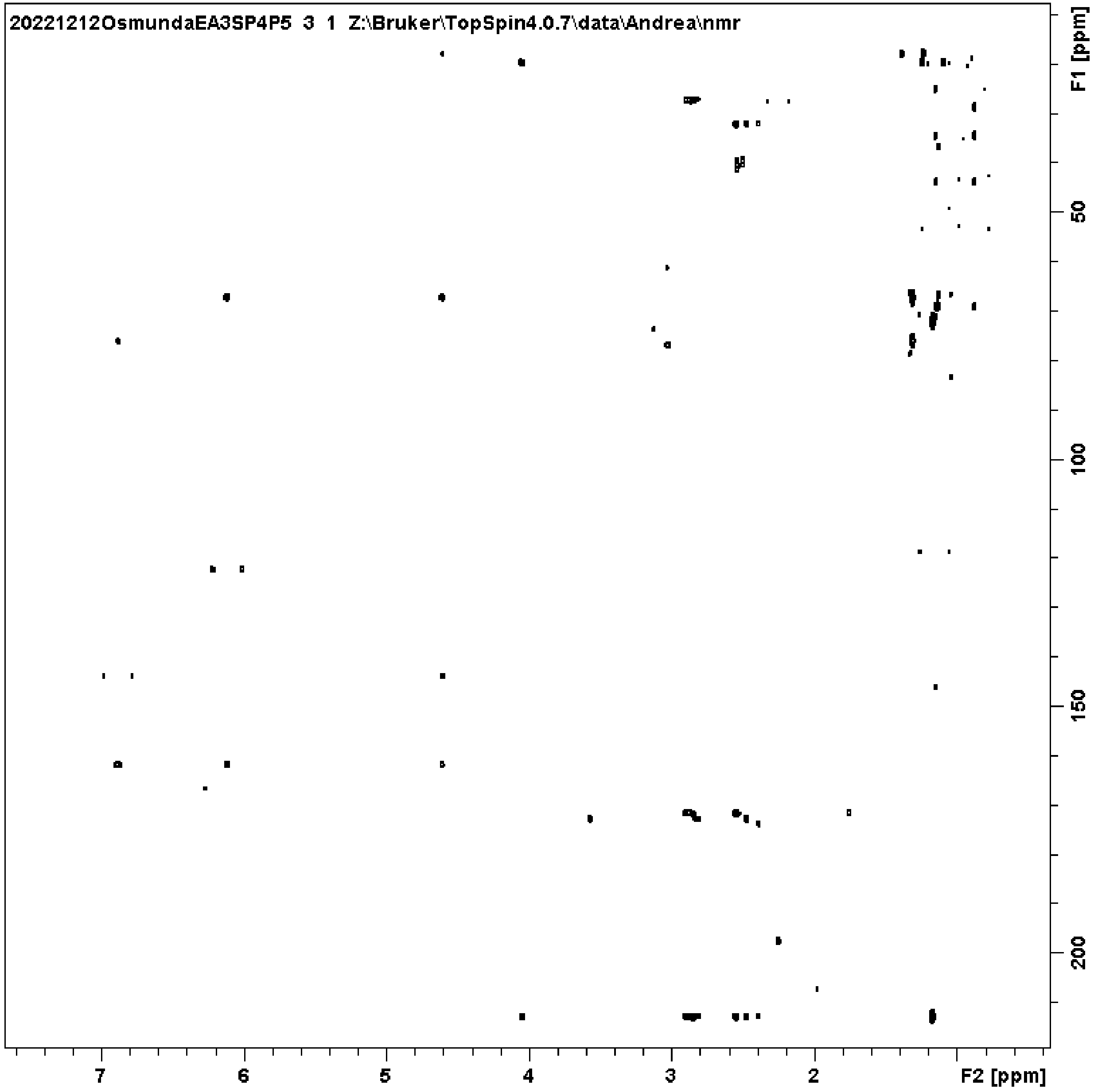

**Figure S48. 2D $^{1}H$-$^{13}C$ HSQC NMR spectrum of 4-*O*-(5-hydroxy-4-oxohexanoyl)-osmundalactone (15)**

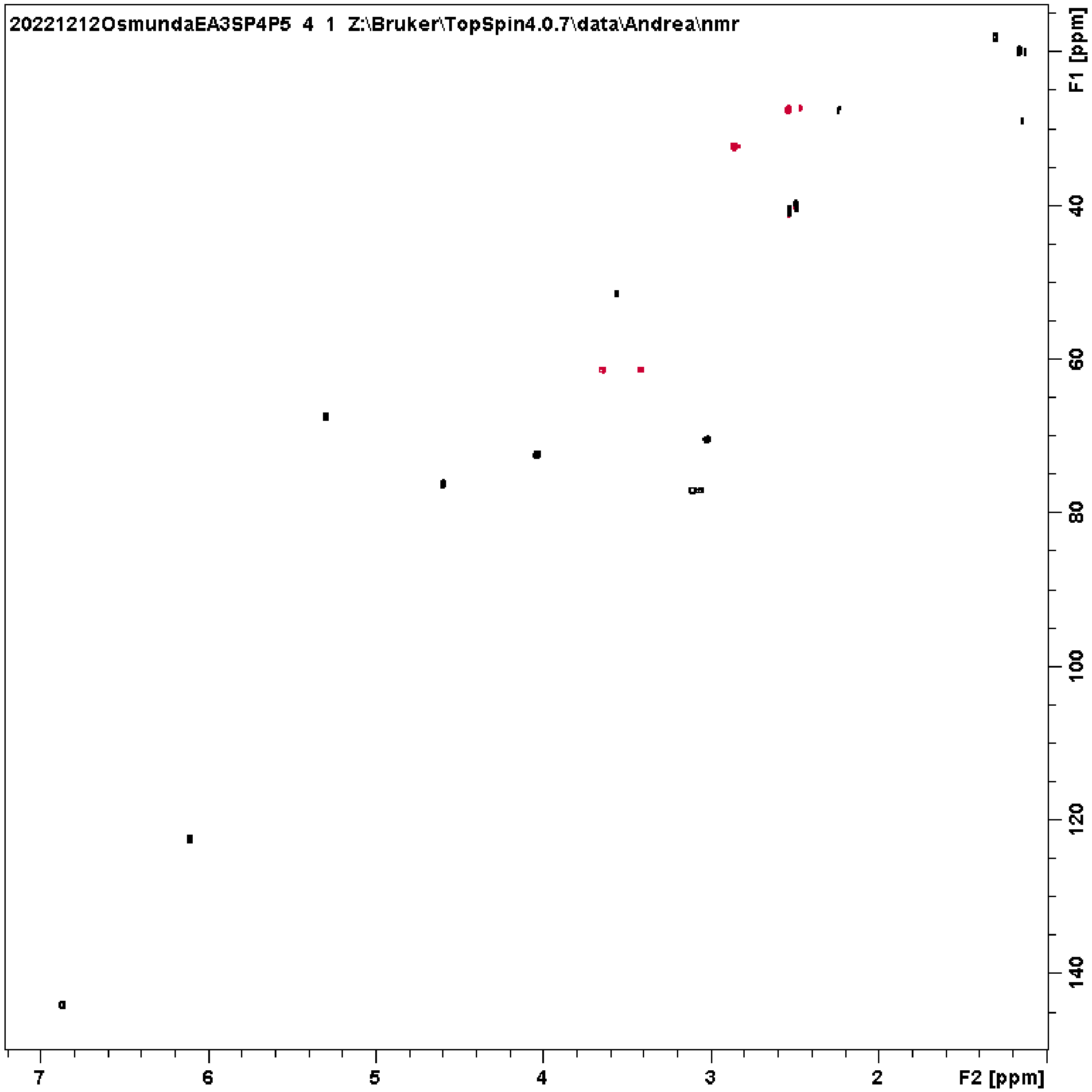

**Figure S49. 2D $^{1}H$-$^{13}C$ HSQC-TOCSY NMR spectrum of 4-*O*-(5-hydroxy-4-oxohexanoyl)-osmundalactone (15)**

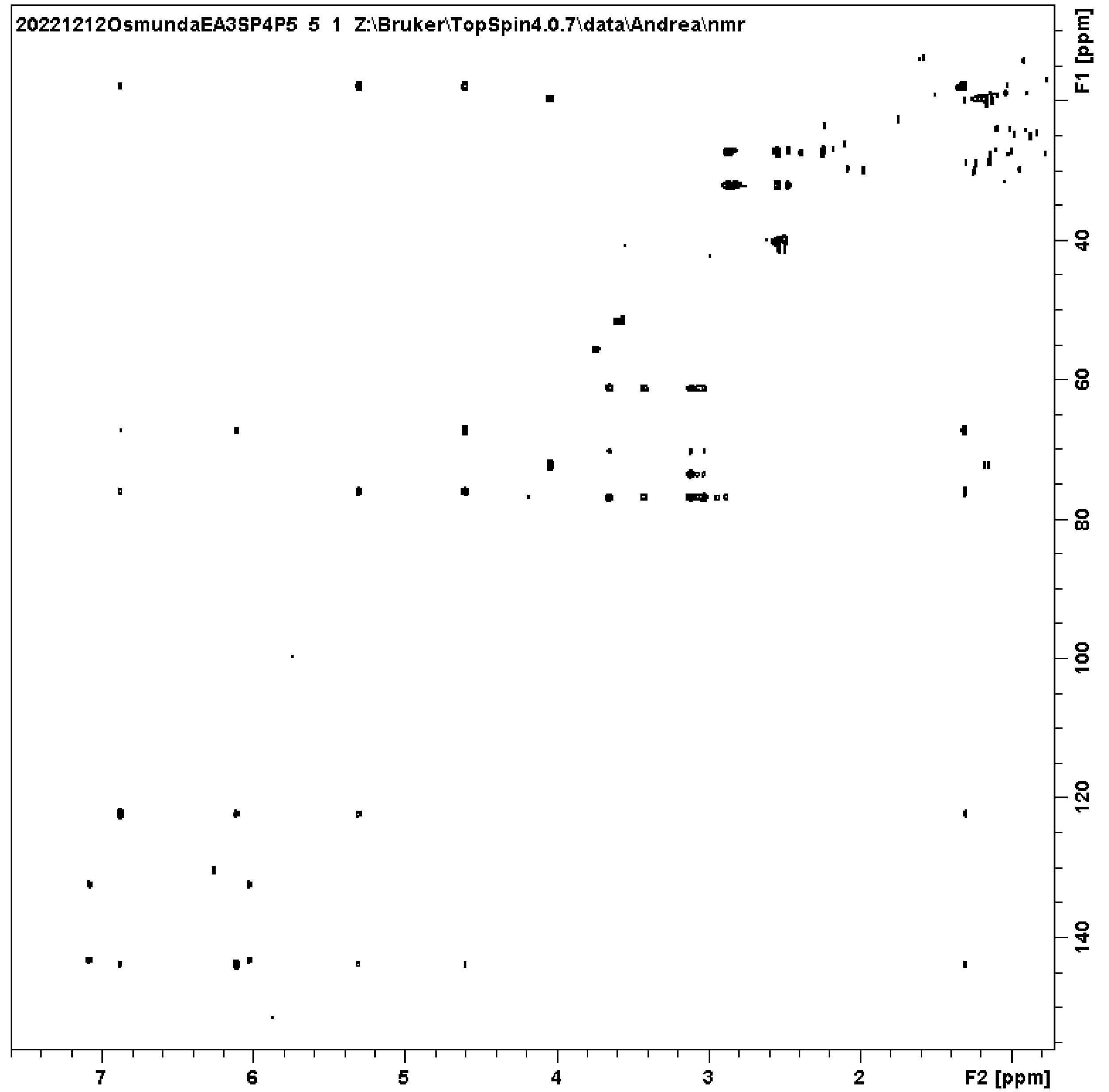

**Figure S50. 2D $^{1}H$-$^{13}C$ H2BC NMR spectrum of 4-*O*-(5-hydroxy-4-oxohexanoyl)-osmundalactone (15)**

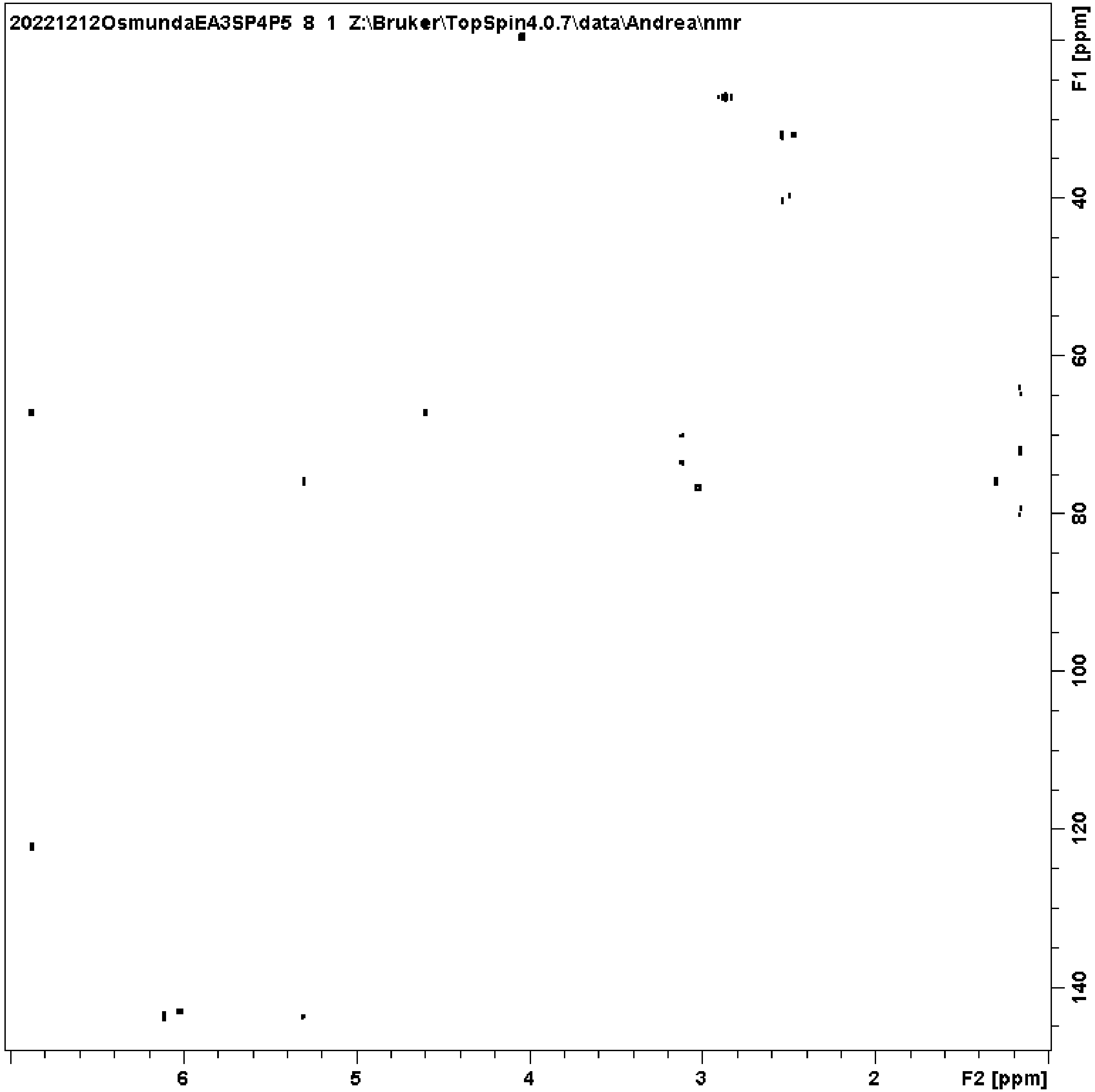

**Figure S51. 2D $^{1}H$-$^{1}H$ COSY NMR spectrum of 4-*O*-(5-hydroxy-4-oxohexanoyl)-osmundalactone (15)**

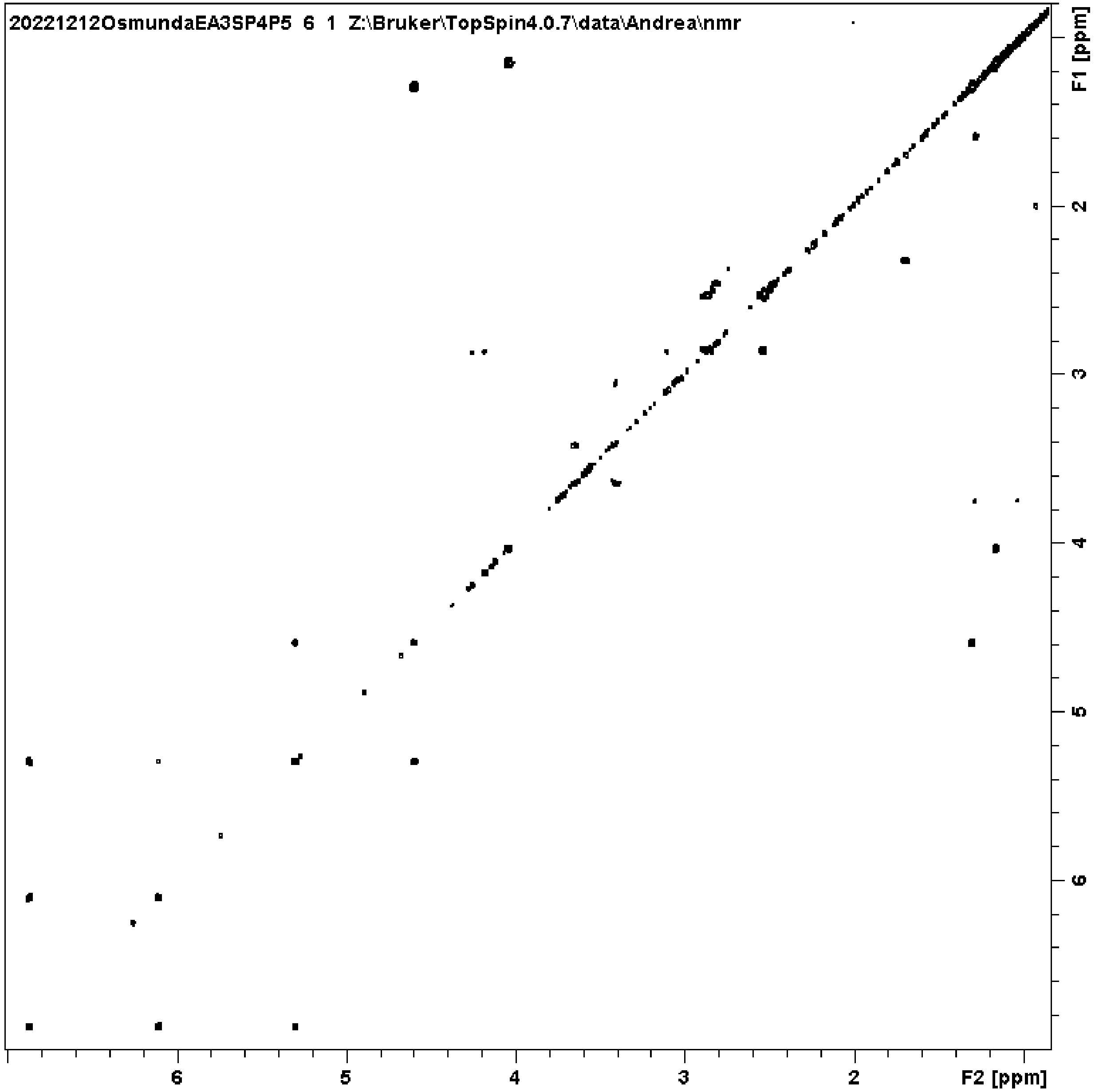

**Figure S52. 2D $^{1}H$-$^{1}H$ ROESY NMR spectrum of 4-*O*-(5-hydroxy-4-oxohexanoyl)-osmundalactone (15)**

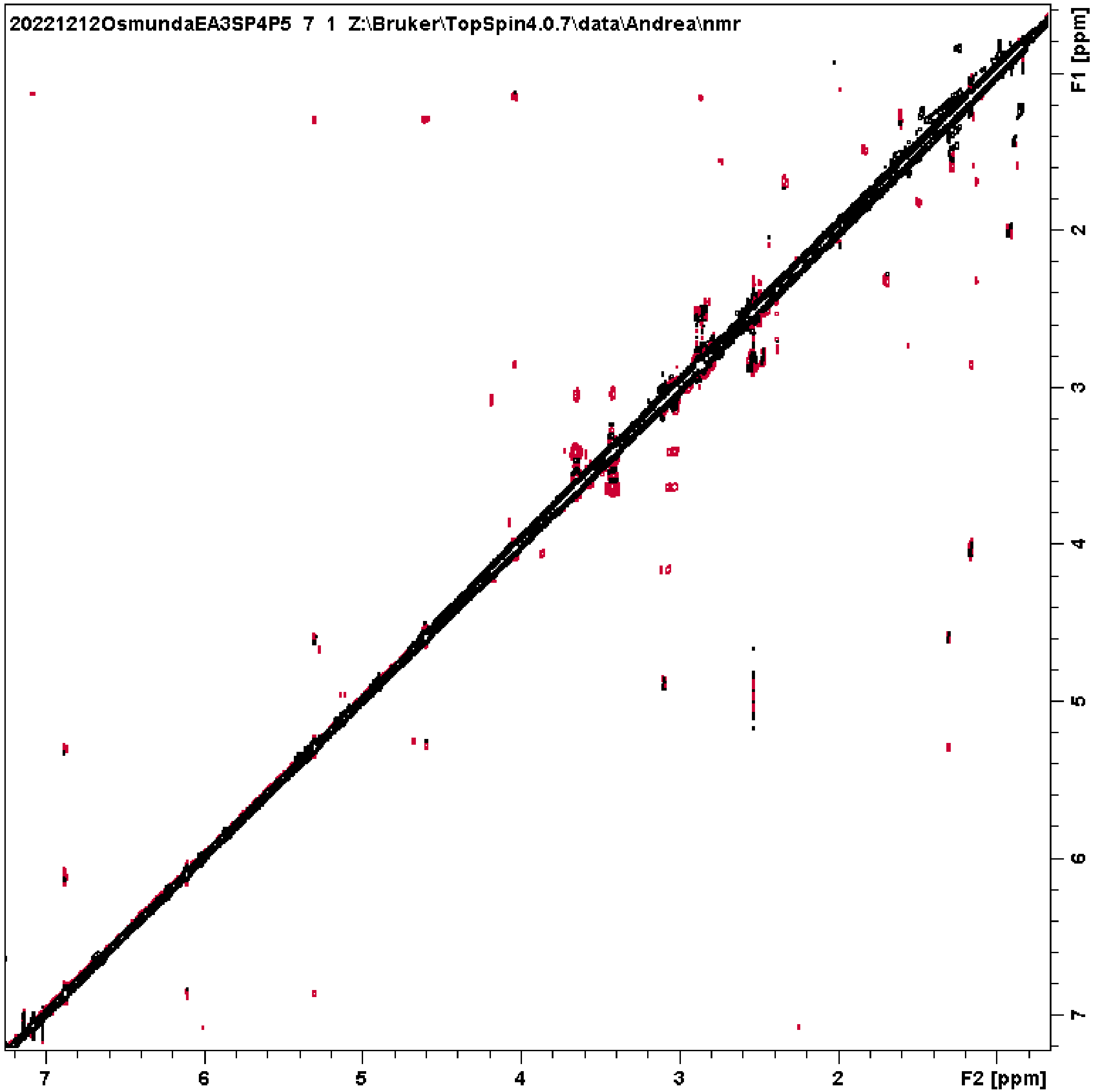

# Figure S53. High resolution mass spectrum of kaempferol 3-*O*-(2”-*O*-*β*-glucopyranosyl-(2’”-*O*-*α*-rhamnopyranosyl))-*β*-glucopyranoside (2)

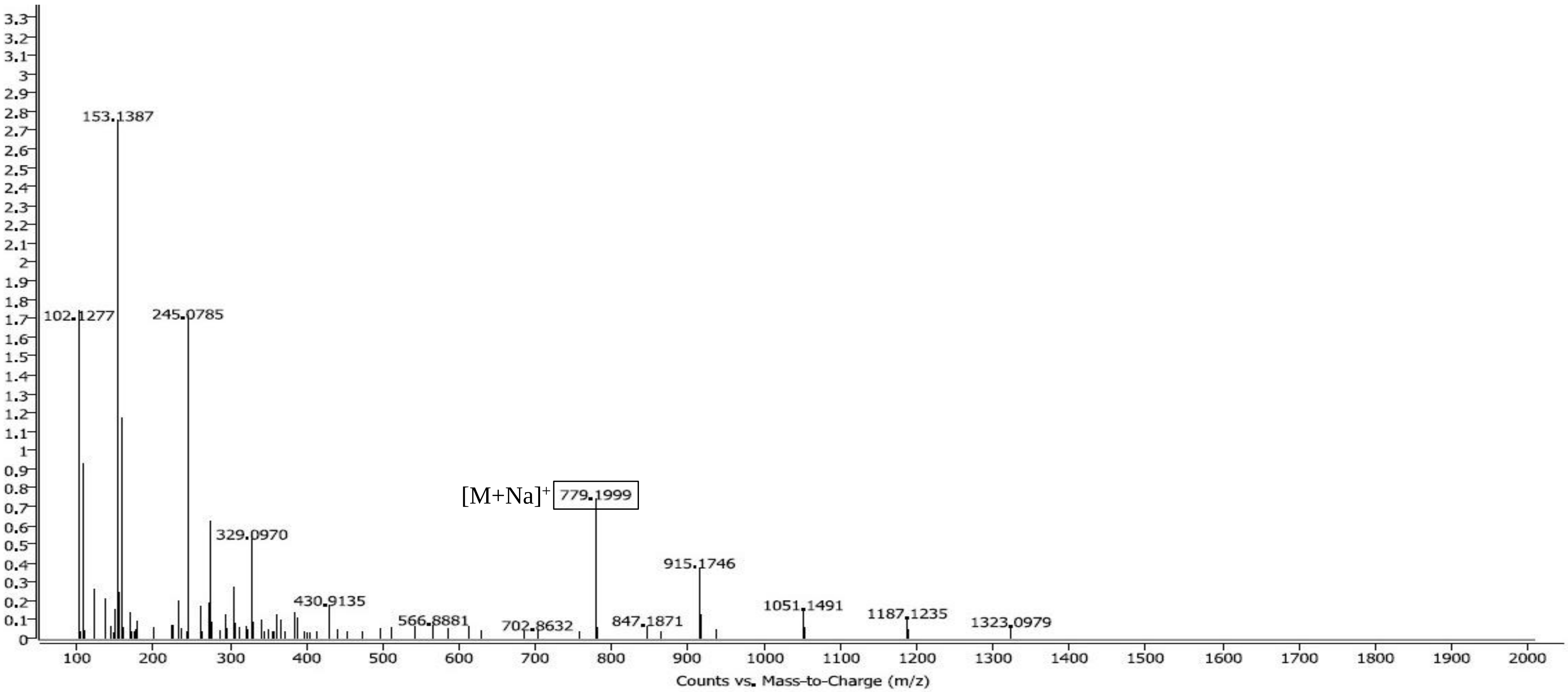

**Figure S54. High resolution mass spectrum of quercetin 3-*O*-(2”-*O*-*β*-glucopyranosyl-(2’”-*O*-*α*-rhamnopyranosyl))-*β*-glucopyranoside (3)**

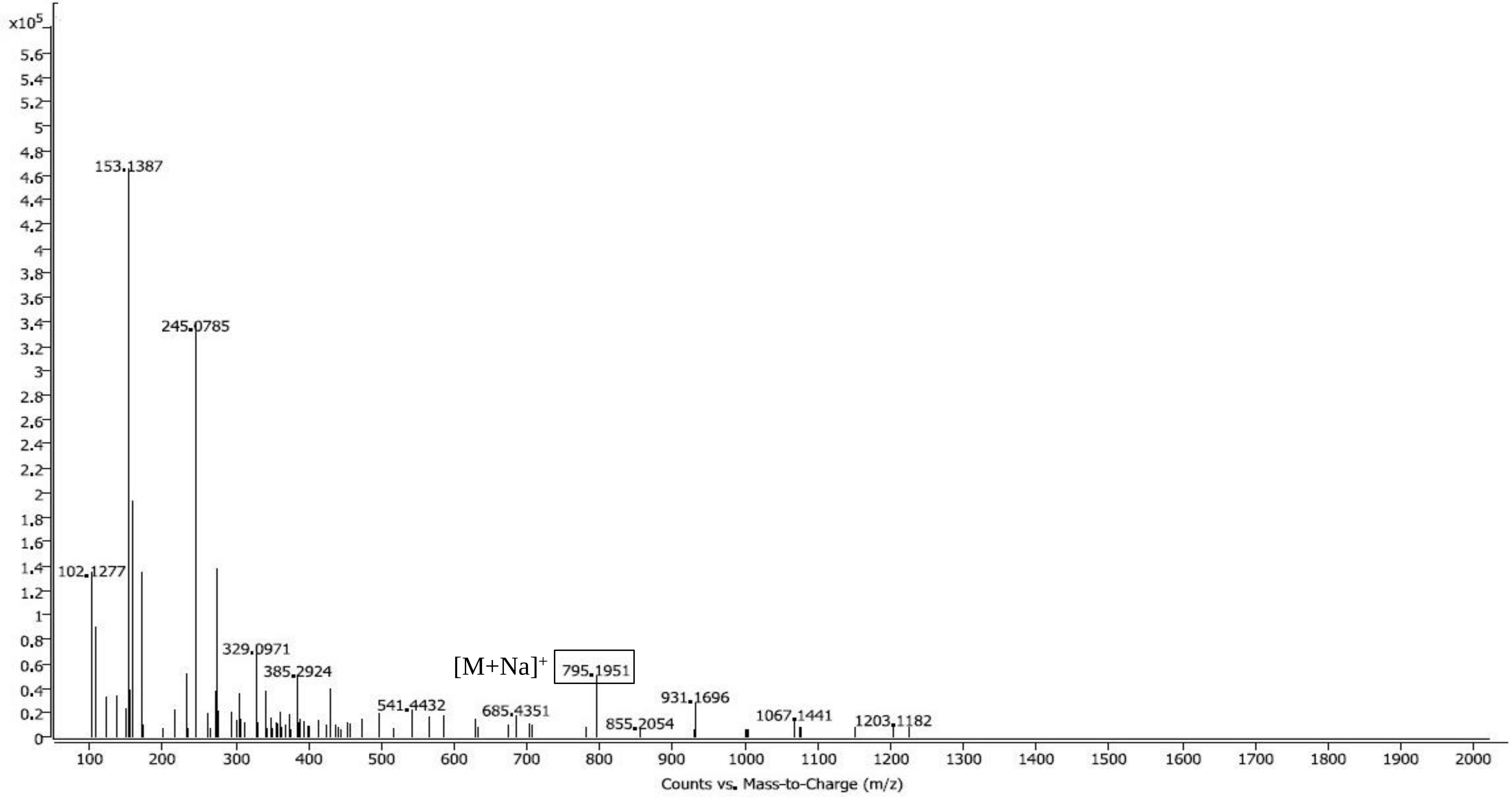

**Figure S55. High resolution mass spectrum of kaempferol 3-*O*-(2”-*O*-*β*-glucopyranosyl-(2’”-*O*-*α*-rhamnopyranosyl-6’”-*O*-(*E*)-caffeoyl))-*β*-glucopyranoside (6)**

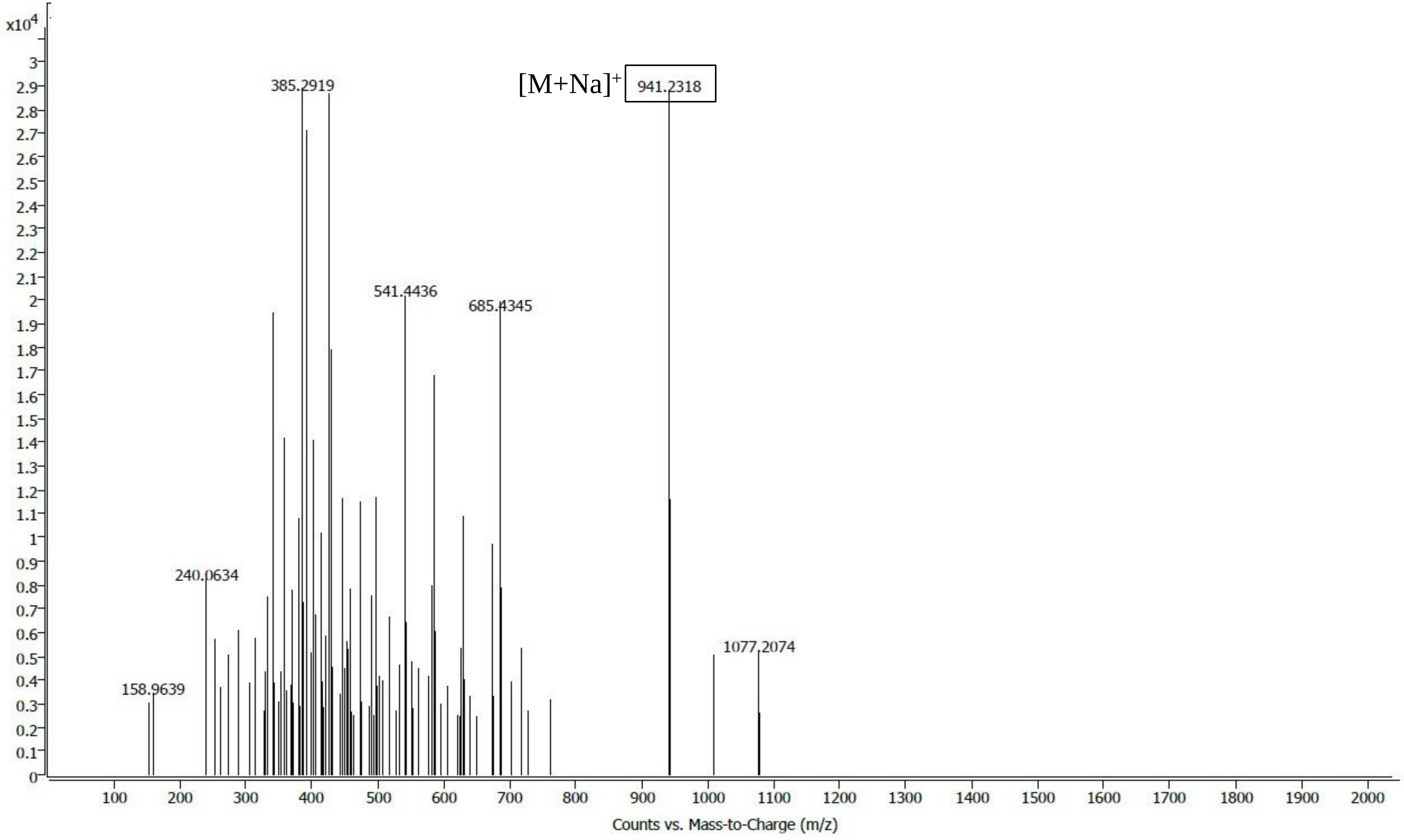

# Figure S56. High resolution mass spectrum of 3-methoxy-5-hydroxy-4-olide (12)

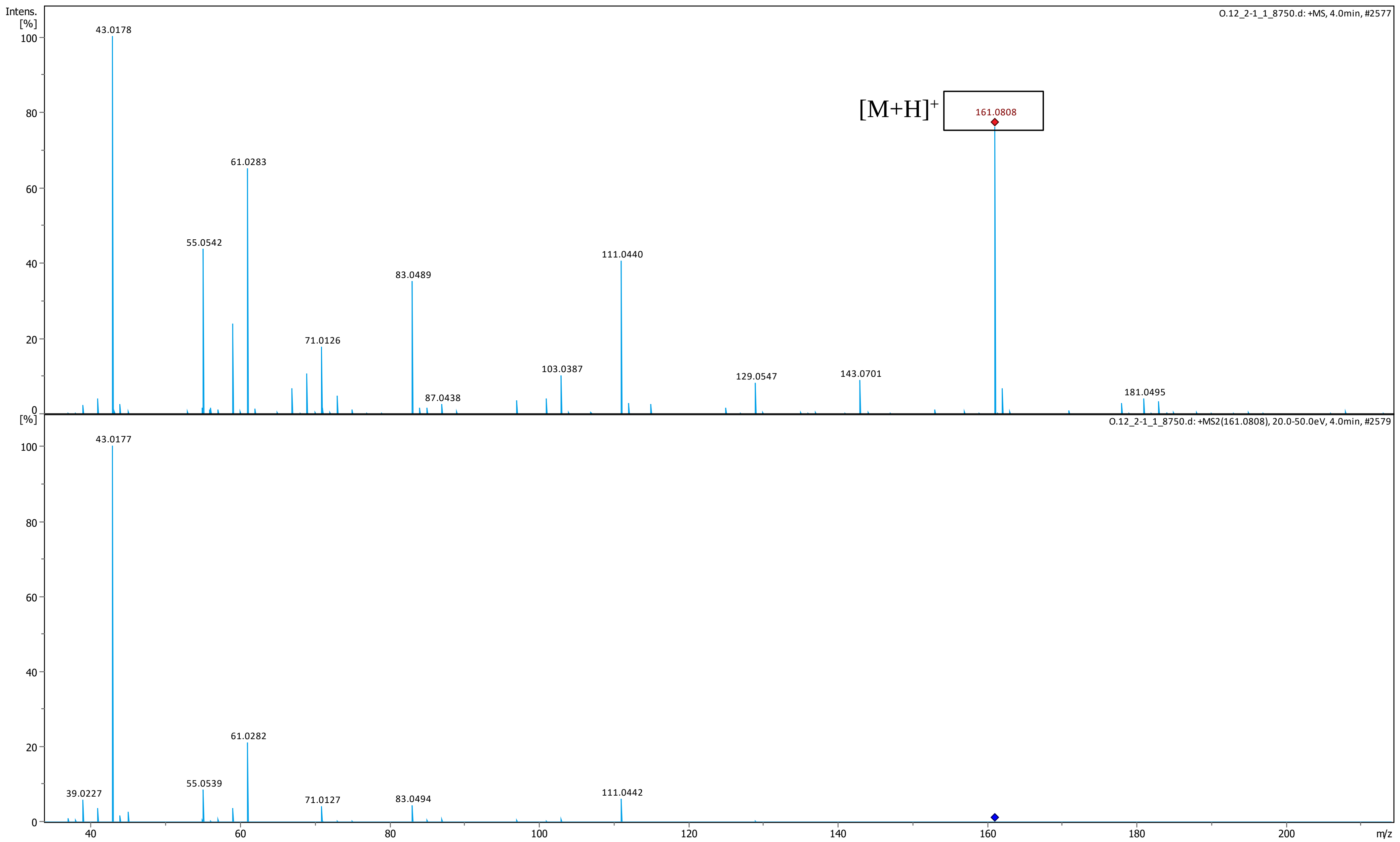

**Figure S57. High resolution mass spectrum of 4-hydroxy-3(3'-hydroxy-4'(hydroxyethyl)-oxotetrafuranone-5-methyl tetrahydropyranone (13)**

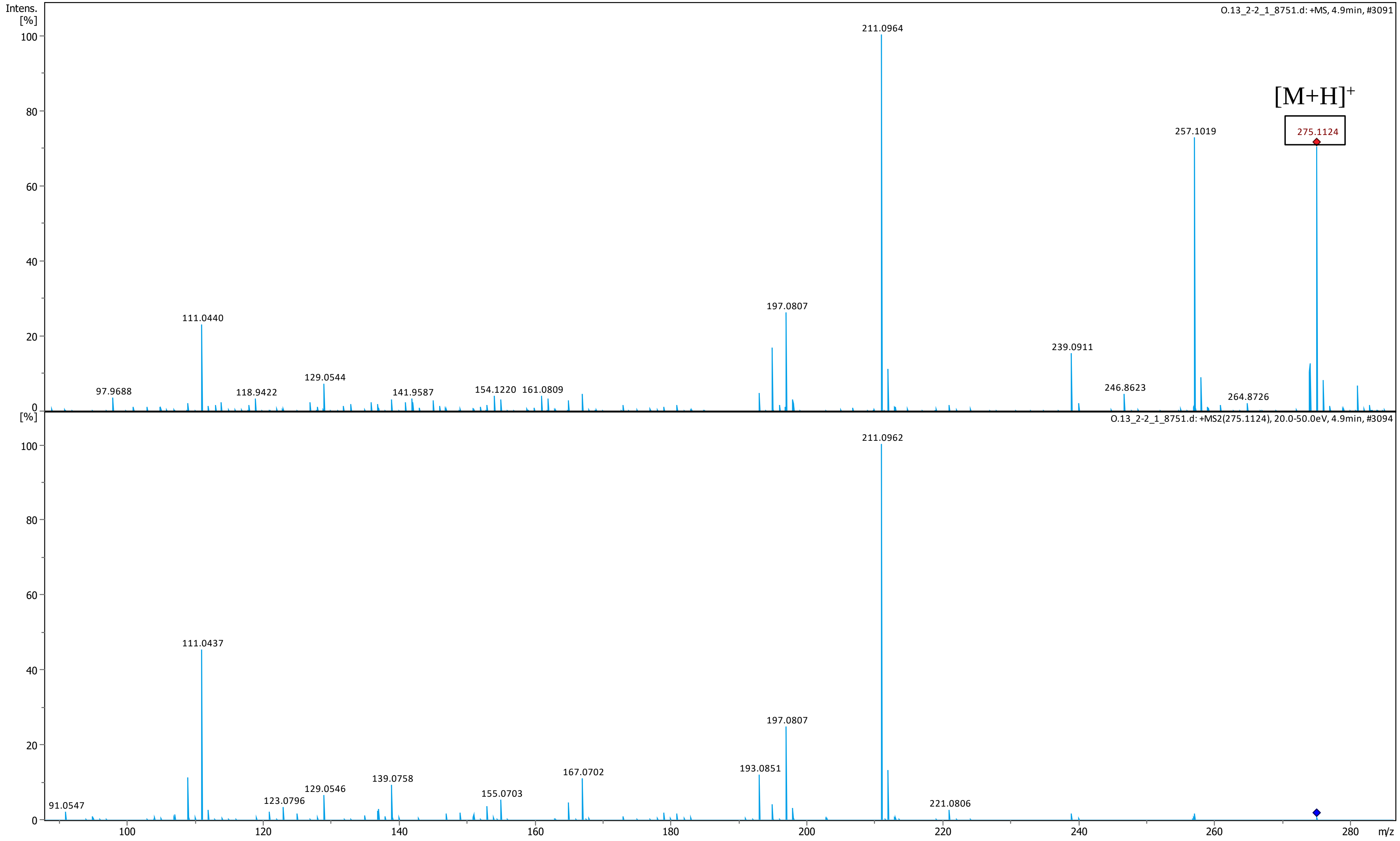

# Figure S58. High resolution mass spectrum of osmunda lactone 4-*O*-(5-hydroxy-4-ketohexanoyl) (15)

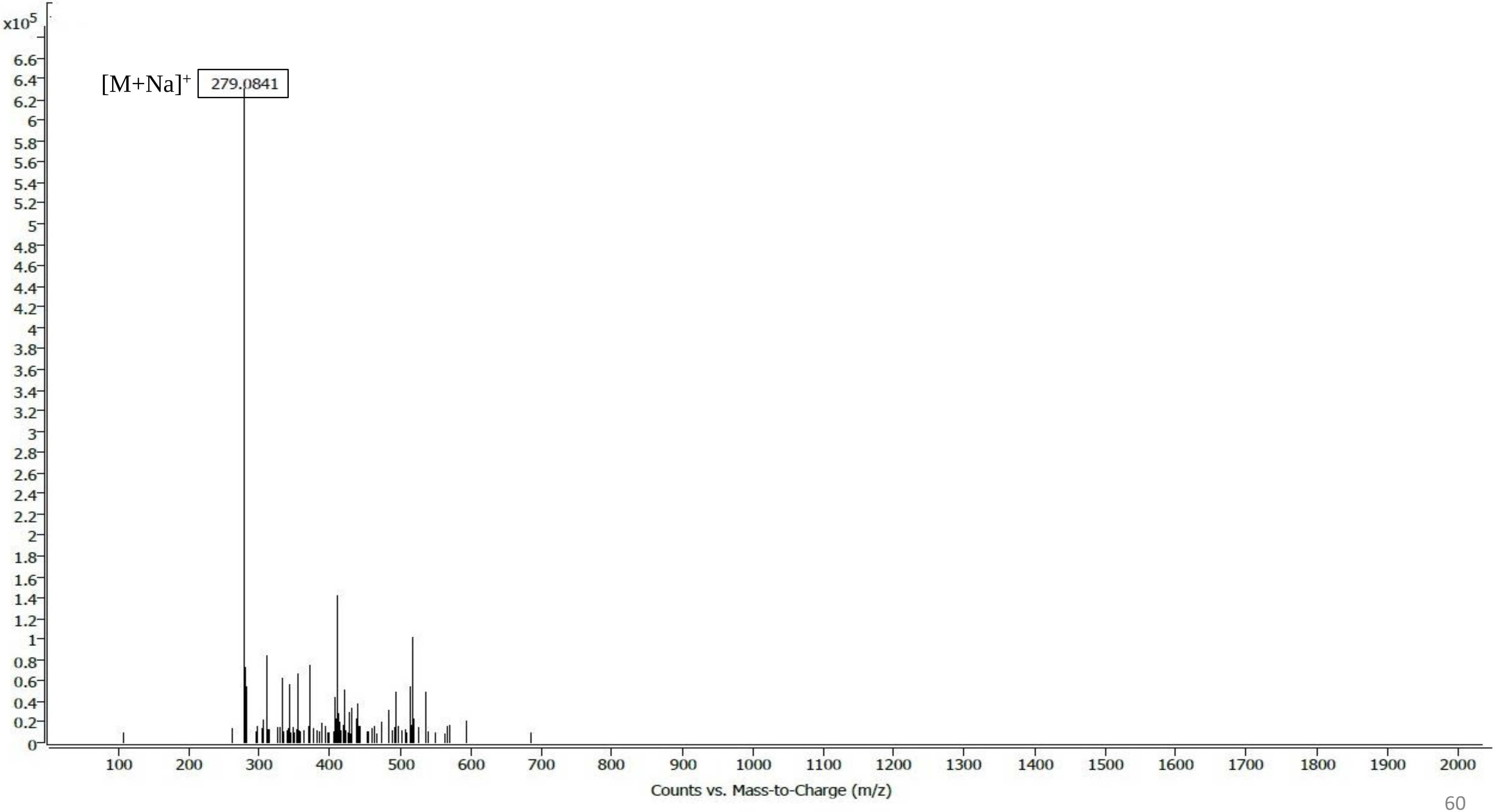

**Figure S59. Circular dichroism (CD) spectra of dihydrodehydrodiconiferyl alcohol 4-*O*-α-rhamnopyranoside (8)**

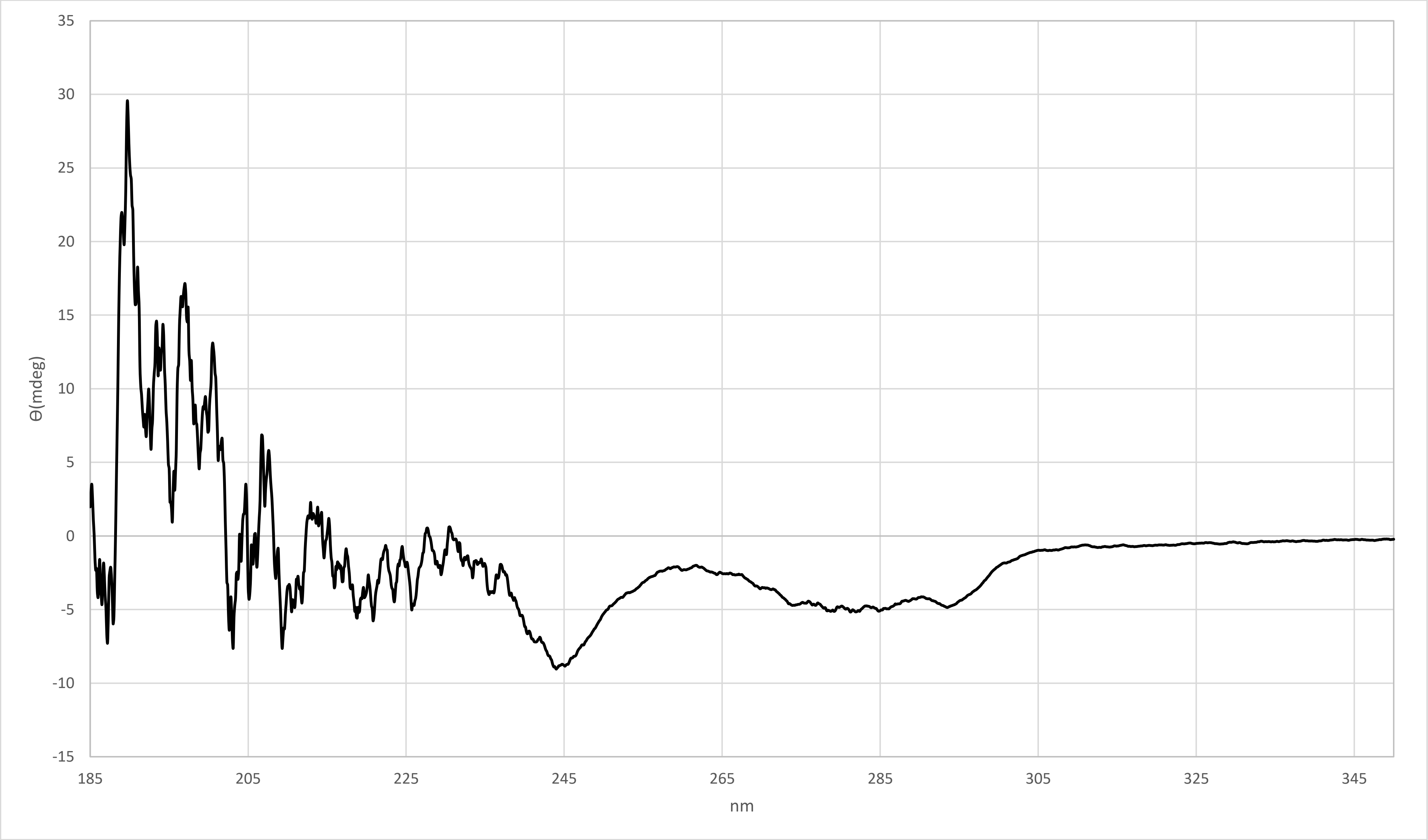

# Figure S60. Circular dichroism (CD) spectra of 3-methoxy-5-hydroxy-4-olide (12)

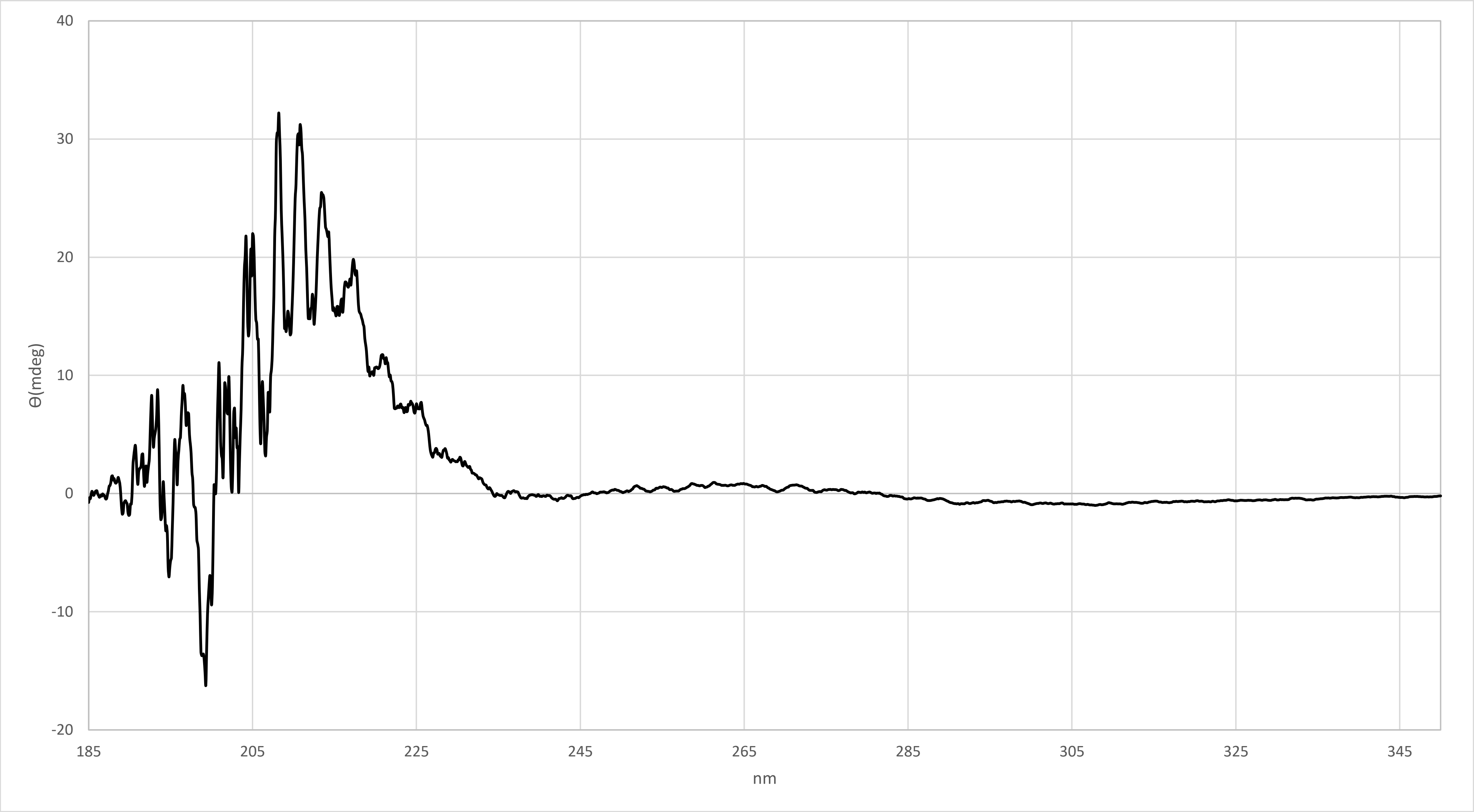

**Figure S61. Circular dichroism (CD) spectra of 4-hydroxy-3(3'-hydroxy-4'(hydroxymethyl)-oxotetrafuranone-5-methyl tetrahydropyranone (13)**

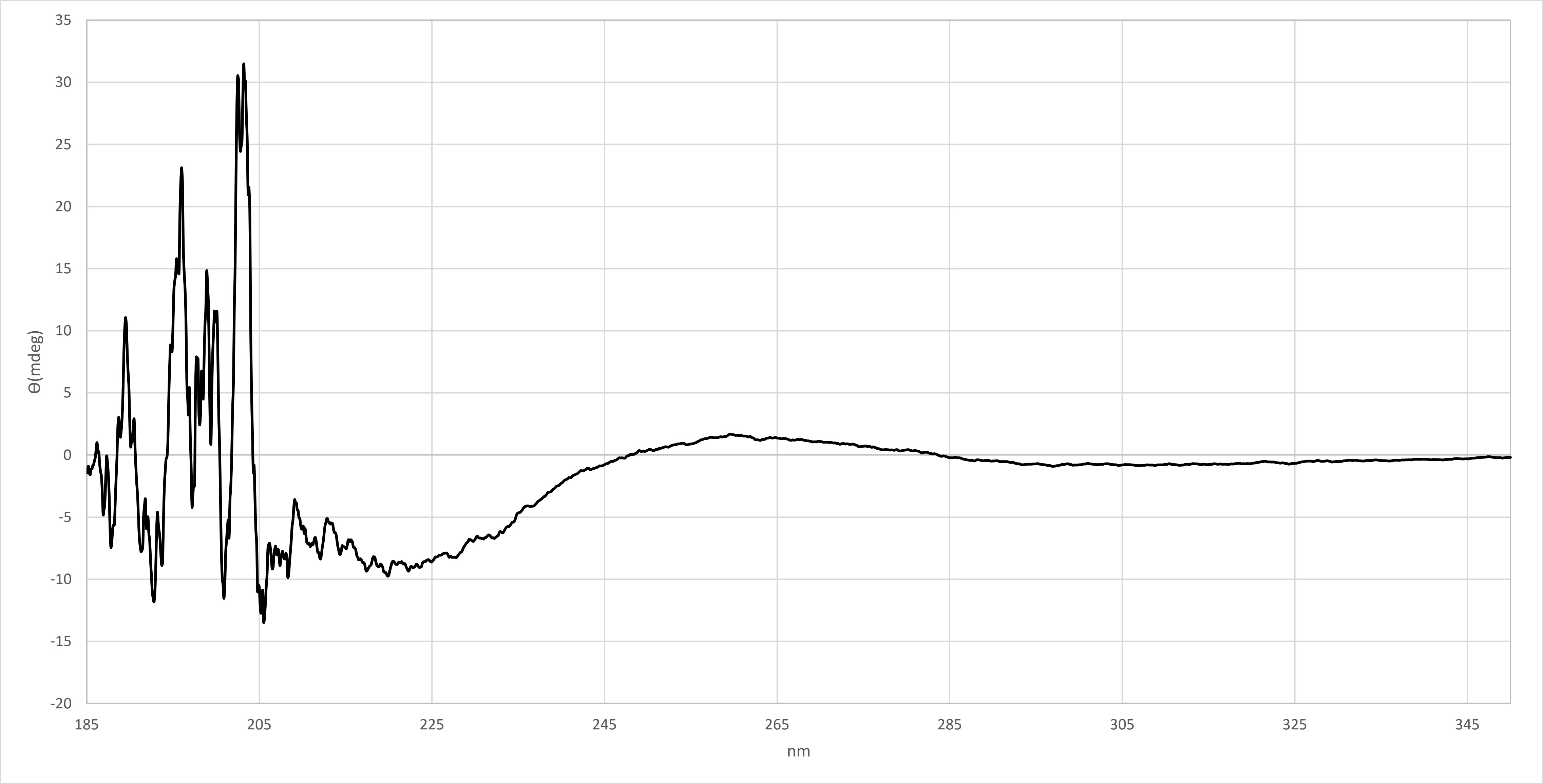

# Figure S62. Circular dichroism (CD) spectra of osmunda lactone 4-O-(5-hydroxy-4-ketohexanoyl) (15)

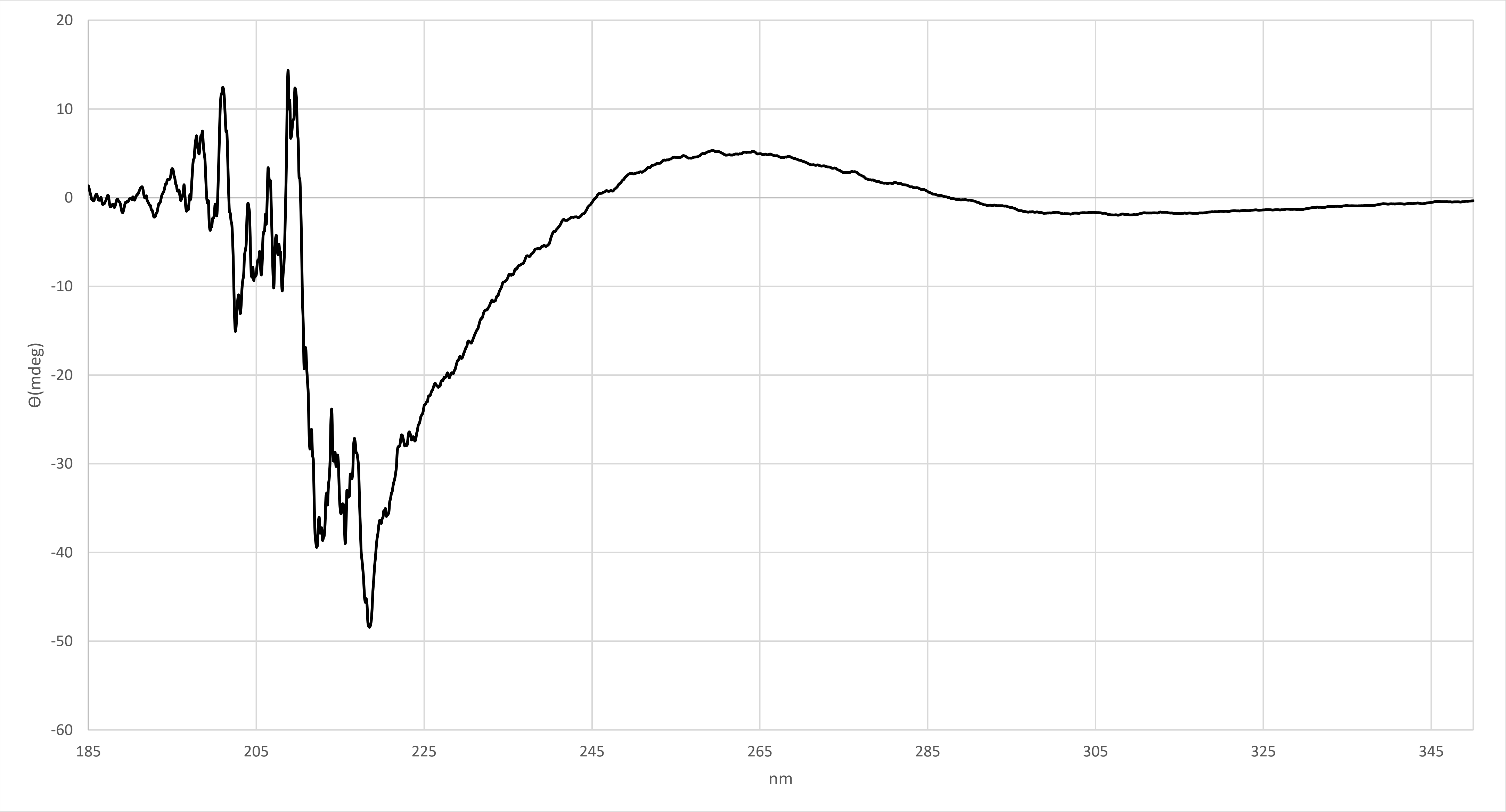

**Figure S63. UV spectrum of kaempferol 3-*O*-(2''-*O*-*β*-glucopyranosyl-(2'''-*O*-*α*-rhamnopyranosyl))-*β*-glucopyranoside (2)**

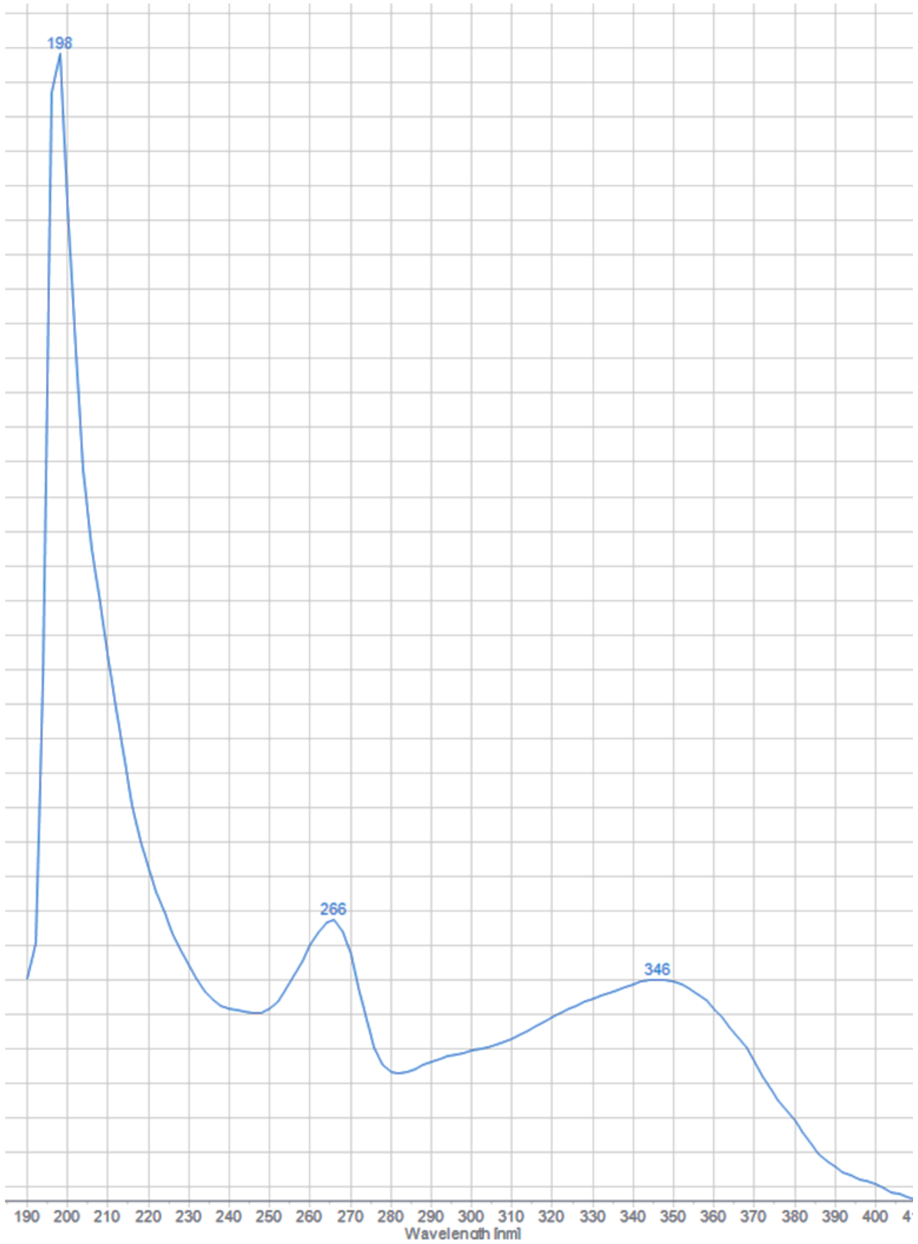

**Figure S64. UV spectrum of quercetin 3-*O*-(2”-*O*-*β*-glucopyranosyl-(2’”-*O*-*α*-rhamnopyranosyl))-*β*-glucopyranoside (3)**

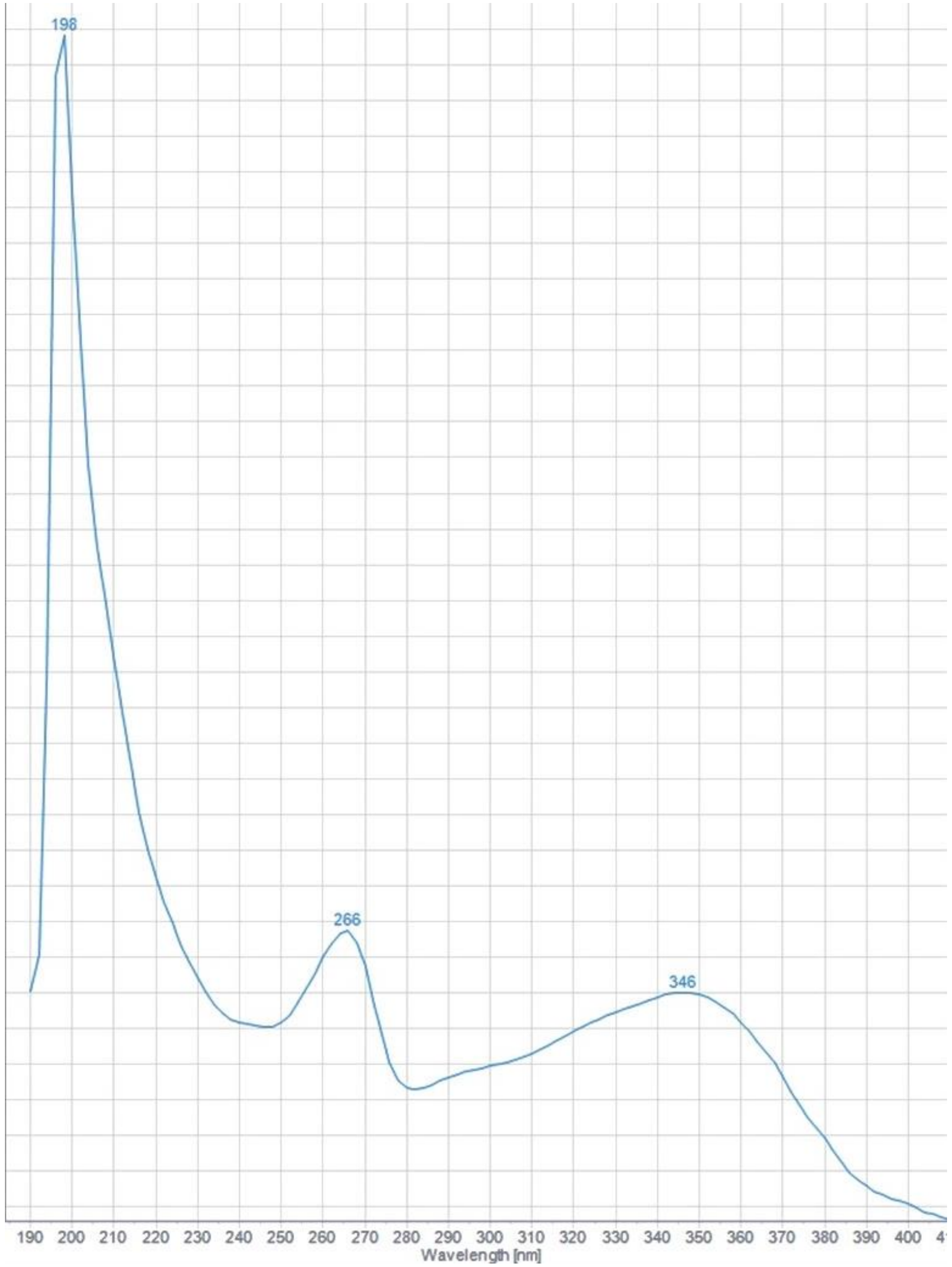

**Figure S65. UV spectrum of kaempferol 3-*O*-(2”-*O*-*β*-glucopyranosyl-(2’”-*O*-*α*-rhamnopyranosyl-6’”-*O*-(*E*)-caffeoyl))-*β*-glucopyranoside (6)**

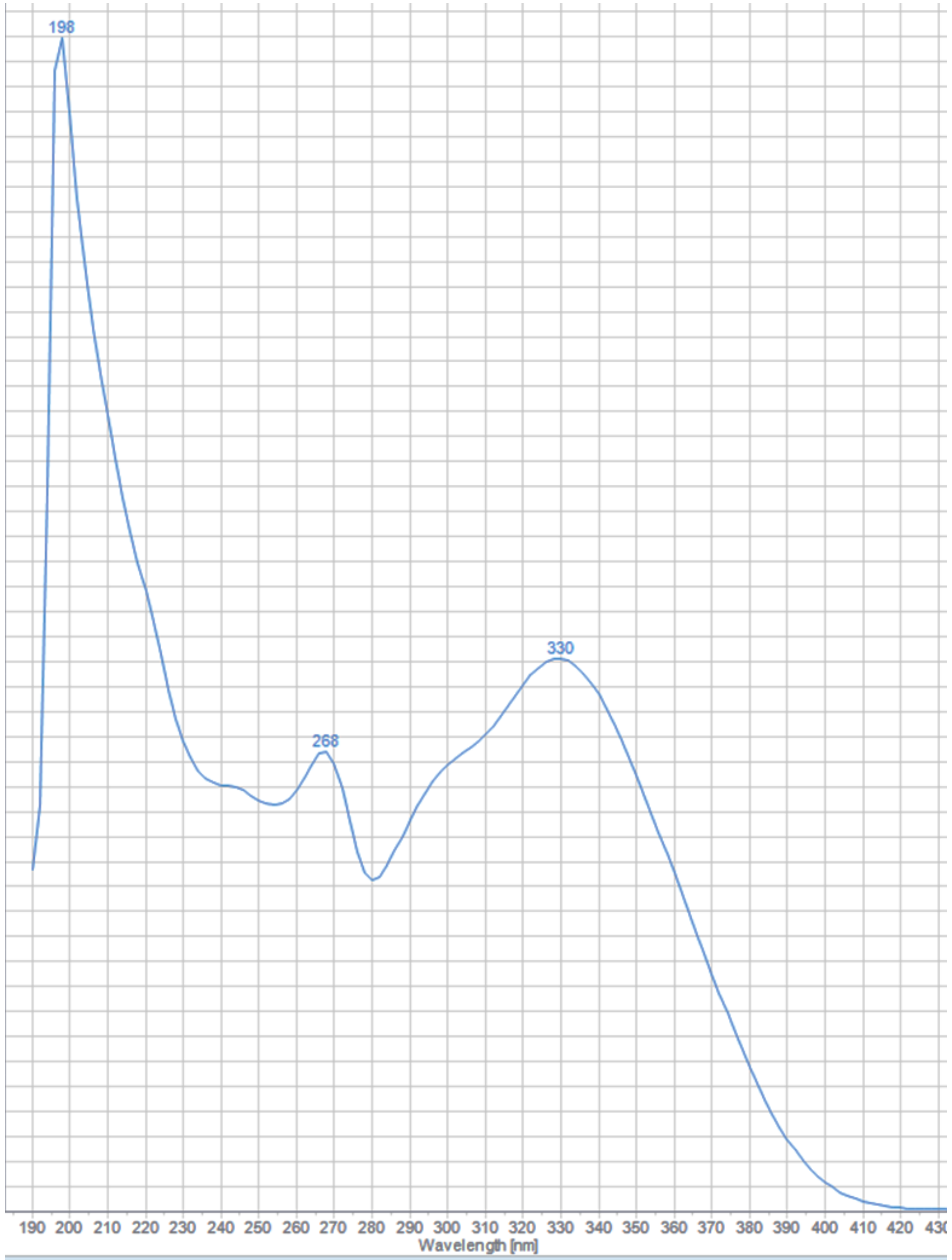

**Figure S66. UV spectrum of 3-methoxy-5-hydroxy-4-olide (12)**

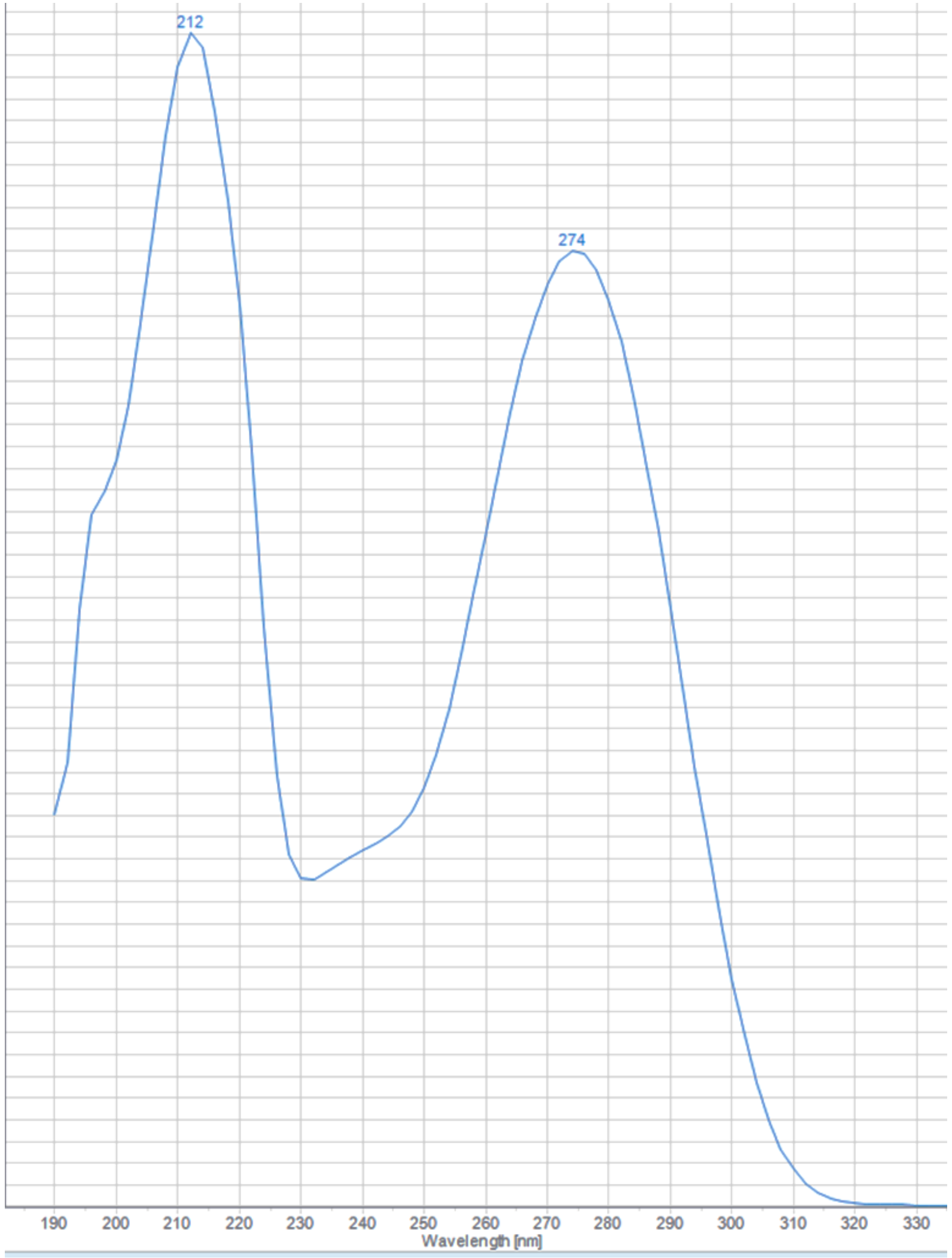

**Figure S67. UV spectrum of 4-hydroxy-3(3’-hydroxy-4’(hydroxyethyl)-oxotetrafuranone-5-methyl tetrahydropyranone (13)**

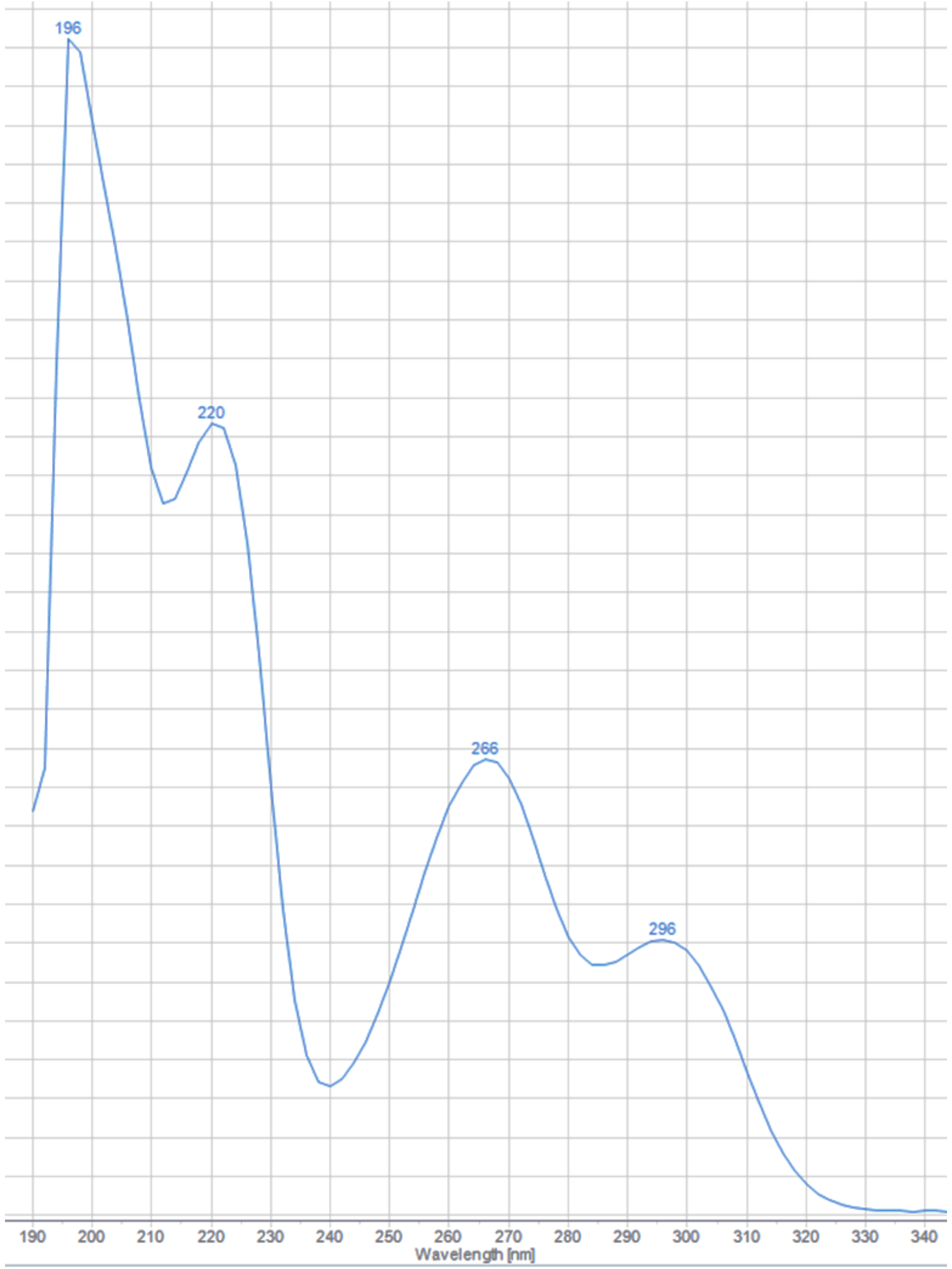

**Figure S68. UV spectrum of osmundalactone 4-*O*-(5-hydroxy-4-oxohexanoyl) (15)**

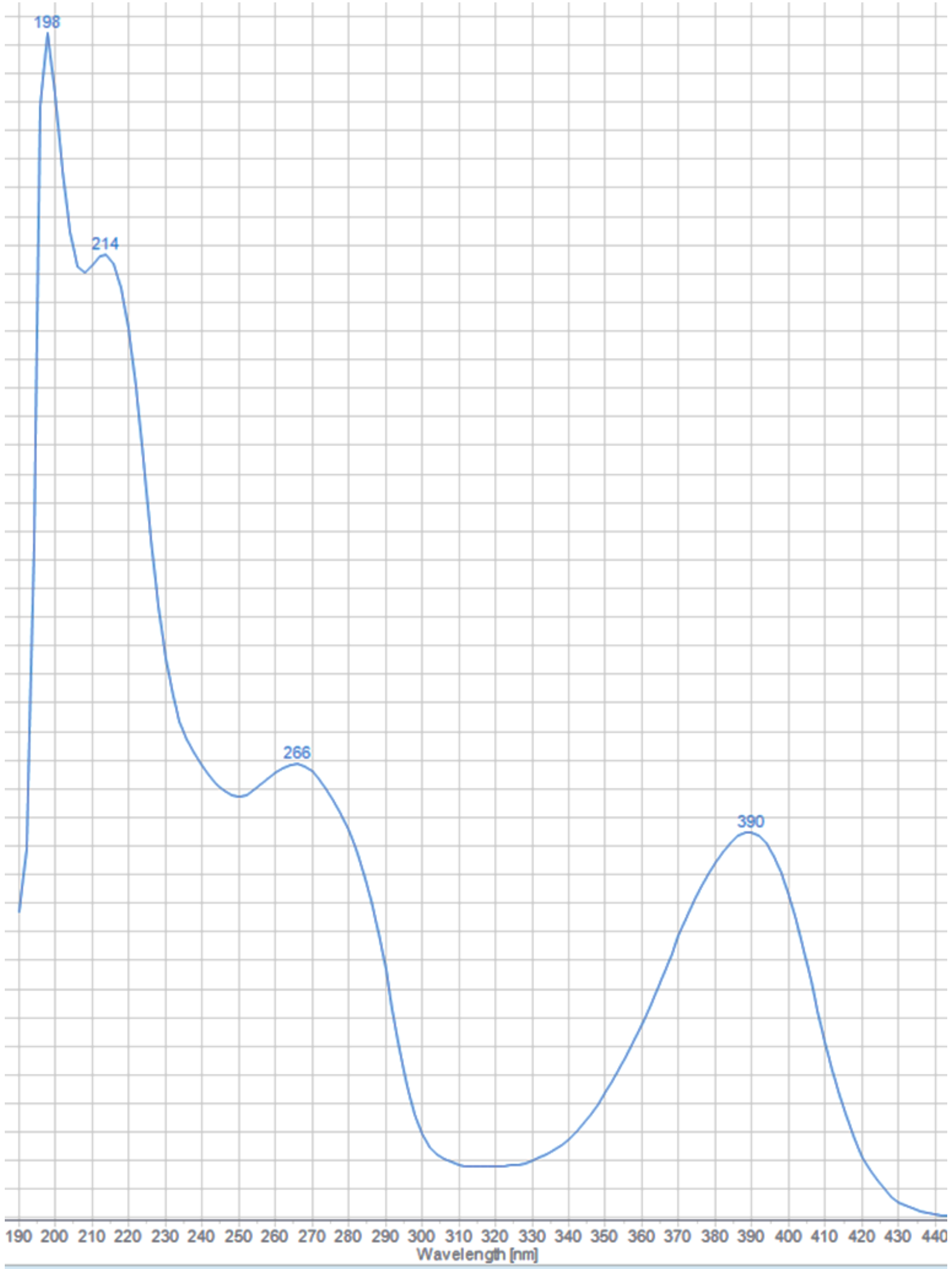

**Supupplemantary T able S1**. $^{1}H$ and $^{13}C$ chemical shift values (ppm) and coupling constants (Hz) of chalconaringenin 2'-*O*-β-glucopyranoside (**1**) in DMSO-$D_6$ at 298K.

| | Compound **1** δ $^{1}H$ | Compound **1** δ $^{13}C$ |
|---|---|---|
| 1 | | 130.0 |
| 2/6 | 7.88 'd' 8.8 | 130.5 |
| 3/5 | 6.36 'd' 8.8 | 115.3 |
| 4 | | 161.3 |
| 1' | | 104.2 |
| 2' | | 159.1 |
| 3' | 6.12 d 2.3 | 94.3 |
| 4' | | 160.9 |
| 5' | 6.10 d 2.3 | 96.5 |
| 6' | | 159.9 |
| α | 7.97 d 15.7 | 120.0 |
| β | 8.01 d 15.7 | 135.1 |
| C=O | | 188.5 |
| 4-OH | 10.22 *s* | |
| 4'-OH | 9.90 *s* | |
| 6'-OH | 10.20 *s* | |
| **2'-O-β-glc** | | |
| 1'' | 4.93 d 7.7 | 100.2 |
| 2'' | 3.34 m | 73.6 |
| 3'' | 3.31 m | 77.0 |
| 4'' | 3.22 dd 9.8; 9.0 | 69.6 |
| 5'' | 3.24 ddd 2.2; 5.4; 9.7 | 60.6 |
| 6A'' | 3.69 dd 2.2; 11.9 | 60.6 |
| 6B'' | 3.49 dd 5.4; 11.9 | |

**Supupplemantary Table S2**. $^{1}H$ and $^{13}C$ chemical shift values (ppm) and coupling constants (Hz) of vanillic acid (**4**) in DMSO-$D_6$ at 298K.

| | Compound **4** δ $^{1}H$ | Compound **4** δ $^{13}C$ |
|---|---|---|
| 1 | | 121.66 |
| 2 | 7.42 d 1.9 | 112.78 |
| 3 | | 147.28 |
| 4 | | 151.16 |
| 5 | 6.82 d 8.1 | 115.08 |
| 6 | 7.42 dd 8.1; 1.9 | 123.52 |
| 7 | | 167.25 |
| 7-COOH | 12.46 s (br) | |
| 4-OH | 9.81 s | |
| 3-$OCH_3$ | 3.79 s | 55.60 |

**Supplementary Table S3**. $^{1}H$ and $^{13}C$ chemical shift values (ppm) and coupling constants (Hz) of p-hydroxy-benzoic acid (**5**) and p-hydroxy-benzoic acid methyl ester (**7**) in DMSO-$D_6$ at 298K.

| | Compound **5** δ $^{1}H$ | Compound **7** δ $^{1}H$ | Compound **5** δ $^{13}C$ | Compound **7** δ $^{13}C$ |
|---|---|---|---|---|
| 1 | | | 121.41 | 120.32 |
| 2/6 | 7.77 ‘d’ 8.6 | 7.80 ‘d’ 8.9 | 131.58 | 131.47 |
| 3/5 | 6.80 ‘d’ 8.6 | 6.83 ‘d’ 8.9 | 115.17 | 115.40 |
| 4 | | | 161.60 | 162.02 |
| 7 | | | 167.21 | 166.11 |
| 7-COOH | 12.39 s (br) | 3.77 s | | 51.68 |
| 4-OH | 10.19 s | 10.31 s (br) | | |

**Supplementary Table S4**. $^{1}H$ and $^{13}C$ chemical shift values (ppm) and coupling constants (Hz) of apigenin 7-(2’ ’-O-α-rhamopyranosyl-β-glucopyranoside) (**9**) in DMSO-D6 at 298K.

| | Compound **9** δ $^{1}H$ | Compound **9** δ $^{13}C$ |
|---|---|---|
| 2 | | 164.33 |
| 3 | 6.87 s | 103.26 |
| 4 | | 182.05 |
| 5 | | 161.19 |
| 6 | 6.37 d 2.2 | 99.39 |
| 7 | | 162.61 |
| 8 | 6.78 d 2.2 | 94.57 |
| 9 | | 157.05 |
| 10 | | 105.49 |
| 1’ | | 121.08 |
| 2’/6’ | 7.93 ‘d’ 8.8 | 128.65 |
| 3’/5’ | 6.93 ‘d’ 8.8 | 116.09 |
| 4’ | | 161.45 |
| **7-*O*-β-glc** | | |
| 1” | 5.22 d 7.7 | 97.88 |
| 2” | 3.50 dd 9.2, 7.7 | 76.32 |
| 3” | 3.46 t 9.2 | 77.28 |
| 4” | 3.19 t 9.2 | 69.71 |
| 5” | 3.48 ddd 9.2, 5.8, 1.9 | 77.09 |
| 6A” | 3.70 dd 11.8, 1.9 | 60.54 |
| 6B” | 3.46 m | |
| **2”-*O*-α-rha** | | |
| 1”’ | 5.12 d 1.9 | 100.52 |
| 2”’ | 3.68 dd 3.3, 1.9 | 70.46 |
| 3”’ | 3.32 dd 9.3, 3.3 | 70.54 |
| 4”’ | 3.20 t 9.3 | 71.92 |
| 5”’ | 3.74 dd 9.3, 6.2 | 68.40 |
| 6”’ | 1.19 d 6.2 | 18.14 |

**Supplementary T able S5**. $^{1}H$ and $^{13}C$ chemical shift values (ppm) and coupling constants (Hz) of epoxyconiferyl alcohol (**10**) in DMSO-$D_6$ at 298K.

| | Compound **10** δ $^{1}H$ | Compound **10** δ $^{13}C$ |
|---|---|---|
| 1 | | 132.30 |
| 2 | 6.88 d 2.0 | 110.48 |
| 3 | | 147.59 |
| 4 | | 145.98 |
| 5 | 6.71 d 8.1 | 115.20 |
| 6 | 6.74 ddd 8.1, 2.0, 0.6 | 118.69 |
| 7 | 4.59 dd 4.6, 0.6 | 85.22 |
| 8 | 3.02 m | 53.65 |
| 9A | 4.11 dd 9.1, 7.1 | 70.96 |
| 9B | 3.71 dd 9.1, 3.9 | |
| 3-$OCH_3$ | 3.75 s | 55.67 |
| 4-OH | 8.88 s (br) | |

**Supplementary Table S6**. $^{1}H$ and $^{13}C$ chemical shift values (ppm) and coupling constants (Hz) of 5-hydroxy-2-hexen-4-olide (**11**) in DMSO-$D_6$ at 298K.

| | **Isomer 1** | | **Isomer 2** | |
|---|---|---|---|---|
| | Compound **11** δ $^{1}H$ | Compound **11** δ $^{13}C$ | Compound **11** δ $^{1}H$ | Compound **11** δ $^{13}C$ |
| 1 | - | 173.33 | - | 173.40 |
| 2 | 6.20 dd 5.8, 2.0 | 121.58 | 6.20 dd 5.8, 2.0 | 121.57 |
| 3 | 7.78 dd 5.8, 1.5 | 156.16 | 7.69 dd 5.8, 1.6 | 156.50 |
| 4 | 4.94 m | 87.30 | 4.98 m | 87.12 |
| 5 | 3.79 dd 6.4, 4.8 | 66.60 | 3.84 dd 6.4, 4.5 | 66.16 |
| 6 | 1.11 d 6.4 | 19.17 | 1.08 dd 6.4, 0.6 | 19.15 |

**T able S7**. $^{1}H$ and $^{13}C$ chemical shift values (ppm) and coupling constants (Hz) of Blumenol C glucoside (**14**) in DMSO-$D_6$ at 298K.

| | Compound **14** δ $^{1}H$ | Compound **14** δ $^{13}C$ |
|---|---|---|
| 1 | | 35.99 |
| 2A | 2.33 | 47.04 |
| 2B | 1.87 | |
| 3 | | 198.09 |
| 4 | 5.71 | 124.26 |
| 5 | | 166.04 |
| 6 | 1.90 t 5.4 | 50.20 |
| 7A | 1.78 | 25.10 |
| 7B | 1.37 | |
| 8A | 1.52 | 36.23 |
| 8B | 1.49 | |
| 9 | 3.74 q 6.0 | 73.30 |
| 10 | 1.08 d 6.1 | 19.57 |
| 11 | 0.99 s | 26.82 |
| 12 | 0.92 s | 28.53 |
| 13 | 1.96 d 1.2 | 24.15 |
| **9-*O*-β-glc** | | |
| 1’ | 4.16 d 7.8 | 100.76 |
| 2’ | 2.88 dd 9.0, 7.8 | 73.59 |
| 3’ | 3.11 t 9.0 | 76.93 |
| 4’ | 3.01 dd 9.7, 9.0 | 70.32 |
| 5’ | 3.06 ddd 9.7, 6.0, 2.2 | 76.82 |
| 6A’ | 3.64 dd 11.6, 2.2 | 61.34 |
| 6B’ | 3.40 dd 11.6, 6.0 | |

**Table S8.** $^{1}H$ and $^{13}C$ chemical shift values (ppm) and coupling constants (Hz) of hexyl-β-glucopyranoside (**16**) in DMSO-$D_6$ at 298K.

| | Compound **16** δ $^{1}H$ | Compound **16** δ $^{13}C$ |
|---|---|---|
| 1A | 3.73 m | 68.62 |
| 1B | 3.39 m | |
| 2 | 1.50 m | 29.31 |
| 3 | 1.30 m | 25.27 |
| 4 | 1.24 m | 31.23 |
| 5 | 1.26 m | 22.15 |
| 6 | 0.85 t 7.0 | 14.01 |
| 1’ | 4.08 d 7.8 | 102.91 |
| 2’ | 2.91 dd 9.0, 7.8 | 73.51 |
| 3’ | 3.10 dd 9.0, 8.6 | 76.89 |
| 4’ | 3.02 dd 9.8, 8.6 | 70.16 |
| 5’ | 3.05 ddd 9.8, 5.8, 2.1 | 76.86 |
| 6A’ | 3.64 dd 11.8, 2.1 | 61.16 |
| 6B’ | 3.41 dd 11.8, 5.8 | |

**Table S9.** $^{1}H$ and $^{13}C$ chemical shift values (ppm) and coupling constants (Hz) of 2-hexenoic acid (**17**) in DMSO-$D_6$ at 298K.

| | Compound **17** δ $^{1}H$ | Compound **17** δ $^{13}C$ |
|---|---|---|
| 1 | | 167.17 |
| 2 | 5.75 dt 15.7, 1.6 | 122.15 |
| 3 | 6.80 dt 15.7, 6.9 | 148.68 |
| 4 | 2.14 m | 33.43 |
| 5 | 1.42 p 7.4 | 20.87 |
| 6 | 0.87 t 7.4 | 13.56 |
